# Mobile Crowdsensing in Ecological Momentary Assessment mHealth Studies: A Systematic Review and Analysis

**DOI:** 10.3390/s24020472

**Published:** 2024-01-12

**Authors:** Robin Kraft, Manfred Reichert, Rüdiger Pryss

**Affiliations:** 1 Institute of Databases and Information Systems, Ulm University, 89081 Ulm, Germany; 2 Department of Clinical Psychology and Psychotherapy, Ulm University, 89081 Ulm, Germany; 3 Institute of Clinical Epidemiology and Biometry, University of Würzburg, 97070 Würzburg, Germany

**Keywords:** ecological momentary assessment, mobile crowdsensing, health data acquisition, mobile health, context awareness

## Abstract

As mobile devices have become a central part of our daily lives, they are also becoming increasingly important in research. In the medical context, for example, smartphones are used to collect ecologically valid and longitudinal data using Ecological Momentary Assessment (EMA), which is mostly implemented through questionnaires delivered via smart notifications. This type of data collection is intended to capture a patient’s condition on a moment-to-moment and longer-term basis. To collect more objective and contextual data and to understand patients even better, researchers can not only use patients’ input via EMA, but also use sensors as part of the Mobile Crowdsensing (MCS) approach. In this paper, we examine how researchers have embraced the topic of MCS in the context of EMA through a systematic literature review. This PRISMA-guided review is based on the databases PubMed, Web of Science, and EBSCOhost. It is shown through the results that both EMA research in general and the use of sensors in EMA research are steadily increasing. In addition, most of the studies reviewed used mobile apps to deliver EMA to participants, used a fixed-time prompting strategy, and used signal-contingent or interval-contingent self-assessment as sampling/assessment strategies. The most commonly used sensors in EMA studies are the accelerometer and GPS. In most studies, these sensors are used for simple data collection, but sensor data are also commonly used to verify study participant responses and, less commonly, to trigger EMA prompts. Security and privacy aspects are addressed in only a subset of mHealth EMA publications. Moreover, we found that EMA adherence was negatively correlated with the total number of prompts and was higher in studies using a microinteraction-based EMA (μEMA) approach as well as in studies utilizing sensors. Overall, we envision that the potential of the technological capabilities of smartphones and sensors could be better exploited in future, more automated approaches.

## 1. Introduction

Studies in medicine and psychology are making increasing use of implementing the Daily Life Research [1] paradigm. The idea is to collect data in everyday life, which often reflects circumstances much more faithfully than when collected in environments that are more artificial, such as the clinic or a controlled study environment. In detail, data from everyday life have many advantages, such as that they are collected in real time and participants do not have to think back and can collect data directly at the place where the phenomenon occurred [1]. This mitigates many types of bias, such as recall bias, but also increases others, as these measurements are obtained in uncontrolled environments. To bring Daily Life Research into practice, there are many possibilities from different disciplines—unfortunately, these are so far without standards or broadly known and used guidelines. However, two concepts are often brought into use, Ecological Momentary Assessment (EMA) and Mobile Crowdsensing (MCS). In the medical context, many published solutions use one of these two strategies, although they are often not explicitly categorized as such. When used in combination, EMA and MCS can significantly increase the potential for Daily Life Research by capturing both subjective and objective, ecologically valid, longitudinal data, as well as the context in which these data are collected [2]. Unfortunately, this combination is still exploited too rarely, and when it is, it is often used in an unstructured and non-standardized way, particularly from a technological perspective. For this reason, we reviewed the literature on EMA in the context of mHealth studies.

The main objective of this review was to investigate the use of MCS in mHealth EMA studies. A particular focus was placed on EMA and MCS strategies, sensor usage, and adherence rates in these studies. The criteria we applied to the publications included can be roughly divided into four categories. First, criteria intended to provide a general overview, such as clusters of topics for which EMA and MCS were used, the EMA/MCS use over time, and general descriptive statistics on the publications analyzed. The second major category relates to EMA and MCS strategies, such as assessment, sampling, prompting, and transmission strategies. The third category of criteria addresses *which* sensors are used in EMA-MCS studies and *how* these sensors are used. Fourth, we conducted a meta-analysis to determine whether there are any study conditions and EMA characteristics that influence EMA study adherence. In this context, adherence rates were hypothesized to be influenced by the incorporation of feedback, guidance, monetary compensation, the use of user-owned smartphones, the prompting strategy used, the number of prompts per day, the study duration, the total number of prompts, the number of questions per prompt, and the total number of questions. Our investigations of the literature follow the PRISMA guidelines [3] and are based on the academic databases PubMed, Web of Science, and EBSCOhost.

In this paper, we present details of the search and analysis conducted and discuss major aspects of the results found. This article is structured as follows: Section 2 discusses relevant background information on EMA and MCS, whereas Section 3 discusses related work in this context. Section 4 presents the materials and methods used for the review and analysis. In Section 5, we present the results, while in Section 6 these results and their implications are discussed. Section 7 summarizes the article and provides an outlook on topics that should be investigated further.

## 2. Background

EMA is an approach, originating in behavioral medicine, to assess the current behavior and experiences of a patient or subject in the context in which it occurs. By applying the methods of EMA, researchers and healthcare providers can gain insights through more direct and timely information collected in the natural environment of the patient or subject [4]. An EMA study design typically involves the subject completing reports several times a day over a period of several days or weeks. According to Smyth and Stone [4], the signal for the subject to begin filling out reports is (1) sent at a frequency most appropriate for the study design and (2) typically sent via an electronic device (e.g., smartphone). The content of the report may focus on experiences that are imminent (e.g., what is the current status) or further in the past and require the subject’s recollection (e.g., the time since the last report). Questions may then address affective, cognitive, health, or behavioral aspects of the subject’s experience [4]. In this way, EMA aims to minimize recall bias, maximize ecological validity, and provide detailed insight into influences on behavior in a natural setting. The combination of EMA and MCS offers advantages beyond the realm of public health and psychology, and can be used not only to collect data that originate from EMA participants, but also to provide (environmental) context through the use of sensors in MCS devices [5,6,7]. While the EMA methods offer advantages in their application, they also have their limitations. Moskowitz and Young [8] note that completing reports as part of EMA may take more time from subjects than meeting with a healthcare professional at regular intervals. In addition, the reports completed by subjects are difficult to verify independently because the data were collected without supervision by a health professional or external control [8].

The term MCS refers to a sufficiently large population or crowd of people contributing data to achieve a common goal, i.e., a *phenomenon of common interest* [9]. Note that, in the context of mHealth, we consider the potential insights gained from both personal and community sensing data as phenomena of common interest [2]. According to Guo et al. [6], this goal is achieved by using mobile devices as a source for data collection and sharing these data with other devices within the crowd. Mobile devices that can be used for MCS are ubiquitous in our daily lives and include smartphones and wearable devices. These devices are usually equipped with sensors (e.g., GPS, accelerometer) that can be used as part of the MCS system [6]. MCS systems rely on active user participation for certain use cases to make data available. The involvement is also one aspect that can be used for classification [9]: in (1) *participatory* crowdsensing [10], the user is actively participating and contributing to the data collection process, whereas in (2) *opportunistic* crowdsensing [11,12], the data sensing, collection, and transmission are happening automatically without any active user participation. Therefore, the user might not be aware of their involvement in the crowdsensing process.

However, the combination of both concepts can be challenging, not only in their technical implementation, but also in their legal implications [2]. Several aspects need to be considered when designing the architecture of an EMA-MCS system. These range from decisions about data collection to the processing of the collected data in a central entity (e.g., a cloud environment). We will not address these aspects within the scope of this work. For a detailed overview, please refer to Capponi et al. [7]. Relevant for the context of this work is the combination of specific strategies as presented by Reuschenbach and Funke [13]. The authors propose a scheme that can be used to classify different studies based on the used assessment and sampling strategies. These sampling strategies include the following. (1) *Interval-contingent*: the measurement takes place at specific intervals (e.g., every hour or every day at the same time). (2) *Signal-contingent*: the measurement starts with a signal that is sent (e.g., an app notification). (3) *Event-contingent*: the starting point for recording a measurement is dependent on the occurrence of an event (e.g., an asthma attack). (4) *Context-sensitive*: the measurement is triggered by a specific external context (e.g., at a specific location). (5) *Continuous*: the measurement takes place continuously. Assessment strategies, in turn, include the following. (A) *Self-assessment*: this participatory assessment strategy refers to the participants performing the measurement themselves. (B) *Surveillance*: an external party (e.g., healthcare provider) conducts the assessment and collects the measurement. (C) *Automated*: a technical device (e.g., a smartphone) is used to automatically collect the measurement.

## 3. Related Work

Related work that also uses the PRISMA guidelines includes the following: De Vries et al. [14] conducted a systematic literature review on well-being. The authors note that the use of smartphones is feasible in the context of an EMA study. The paper also notes that finding relevant literature is a problem because the topic area is relatively new. Dao et al. [15] investigated the use of EMA in the context of health and well-being. The authors conclude that the use of EMA can help promote behavior change. They suggest the use of sensors to optimize data collection while minimizing participant burden. The review of Zapata-Lamana et al. [16] focused on physical activity. The review process also focused on the use of sensors in combination with EMA and their use in the context of physical activity. There are other reviews with similar structures for various other research areas.

There are also recent PRISMA-based reviews in the literature that focus on EMA as a general research method across application areas and also specifically on the study of adherence. Vachon et al. [17] examined studies investigating severe mental disorders. The authors included 79 studies and extracted their adherence and retention rates, as well as a set of study characteristics, to examine the relationship between these variables. The results suggest that adherence and retention rates are lower for studies with a higher proportion of male participants and for participants diagnosed with a psychotic disorder. Conversely, adherence rates were found to be positively related to the use of a fixed sampling strategy, higher incentives, greater time intervals between consecutive assessments, and fewer assessments per day. Similar to the work at hand, a meta-analysis by Wrzus et al. [18] analyzed 477 EMA publications in various fields of psychology and related disciplines to examine how study designs and samples predict study adherence and dropout. The authors reported descriptive statistics on study and sample characteristics across the studies reviewed, including sampling strategies and various incentives. In addition, they found that adherence was significantly higher in studies that offered financial incentives, whereas study design or sample characteristics otherwise had little effect on adherence or dropout rates.

In general, we found that EMA research papers tend to cover specific diseases or topics and review the literature for that specific area. In addition, while the reviews by Vachon et al. and Wrzus et al. are structured similarly as the present work, they only include literature from quite narrow research fields (i.e., severe mental disorders) or academic databases (i.e., EBSCOhost). Moreover, the use of sensors in EMA studies is less well researched. Overall, we are not aware of any work that conducts a comprehensive, cross-disciplinary literature review, as this work does, that also focuses specifically on the use of sensors in EMA studies.

Previous works by the authors [2,19], but not in the context of a literature review, have investigated how EMA and MCS can be combined from the conceptual and architectural side. Another work that should be considered is the WHO’s mERA checklist [20], which provides specifications so that mHealth applications become more standardized. However, it does not directly address survey strategies such as EMA or MCS. On a more general note, there is unfortunately still too little interdisciplinary research into how the individual data collection strategies and concepts such as EMA or MCS can be combined to produce even better study results. Therefore, this work shall make further contribution in this context.

## 4. Materials and Methods

To produce results that are transparent, traceable, and reproducible by other researchers, we established a review protocol guided by the *Preferred Reporting Items for Systematic Reviews and Meta-Analyses* (PRISMA) statement. PRISMA is a collection of items designed to promote a systematic approach for systematic reviews and meta-analyses [3]. We used the PRISMA-P extension, which is intended to be used for developing and creating protocols for systematic reviews [21].

### 4.1. Eligibility Criteria

The *eligibility criteria* that were used to select the literature to address these objectives are outlined in the following. First, the inclusion criteria (IC) were as follows:

IC1 The study uses an EMA approach.
IC2 The study utilizes MCS in combination with an EMA approach.
IC3 The type of the specific publication is primary research.

On the other hand, the exclusion criteria (EC) were as follows:

EC1 The study presents no clear EMA approach.
EC2 The quality of the paper is insufficient (e.g., unclear specification of population, treatment, or treatment effect).
EC3 The language of the publication is not English.
EC4 The type of publication is a review, systematic review, meta-analysis, meeting abstract, note, or is not peer-reviewed.

### 4.2. Information Sources

The primary *information sources* we used were databases containing research literature in the field of healthcare. For this purpose, we focused our search on the following three databases: PubMed, Web of Science, and PsycINFO via EBSCOhost Research Databases. These databases provided sufficient coverage in our research area [22]. Google Scholar was not added because the focus was on the medical study context and not on the technical operationalization of EMA and MCS.

### 4.3. Search Strategy

For the *search strategy*, we took into account that each database uses its own language for search queries. Based on the primary search terms, we also included related terms used in that research area. For example, one of our primary search terms was “Ecological Momentary Assessment” and a related term we used was “Electronic Diary”. Based on this approach, we issued search queries on 13 July 2022 that were adapted to the requirements of the three databases PubMed (see Listing 1), Web of Science (see Listing 2), and EBSCOhost (see Listing 3).

**Listing 1.** Search query for the PubMed database.
( ( Ecological Momentary Assessment [ Title/Abstract ]) OR (

  Ecological momentary intervention [ Title/Abstract ]) OR (

  experience sampling method [ Title/Abstract ]) OR ( diary

  assessment [ Title/Abstract ]) OR ( electronic diary [ Title/

  Abstract ]) OR ( Ambulatory Assessment [ Title/Abstract ]) OR

  (Electronic Interview [ Title/Abstract ]) OR ( Real Time

  Assessment [ Title/Abstract ]) )

AND

( smartphone [ Title/Abstract ] OR mobile [ Title/Abstract ] OR

  crowdsensing [ Title/Abstract ] OR MCS [ Title/Abstract ] OR

  sensor [ Title/Abstract ] OR sensing [ Title/Abstract ] )


**Listing 2.** Search query for the Web of Science database.AB = ( "Ecological Momentary Assessment" OR "Ecological   momentary intervention" OR "Experience sampling method"   OR "Diary Assessment" OR "electronic diary" OR "   Ambulatory Assessment" OR "Electronic Interview" OR "Real    Time Assessment" ) AND AB = ( smartphone OR mobile OR crowdsensing OR MCS OR sensor   OR sensing )

**Listing 3.** Search query for the EBSCOhost research database.
( (AB Ecological Momentary Assessment ) OR (AB Ecological 

  momentary intervention ) OR (AB experience sampling method

  ) OR (AB diary assessment ) OR (AB electronic diary ) OR (

  AB Ambulatory Assessment ) OR (AB Electronic Interview ) OR 

   (AB Real Time Assessment ) )

AND

( AB smartphone OR AB mobile OR AB crowdsensing OR AB MCS OR

   AB sensor OR AB sensing )


### 4.4. Study Records

**Figure 1:**
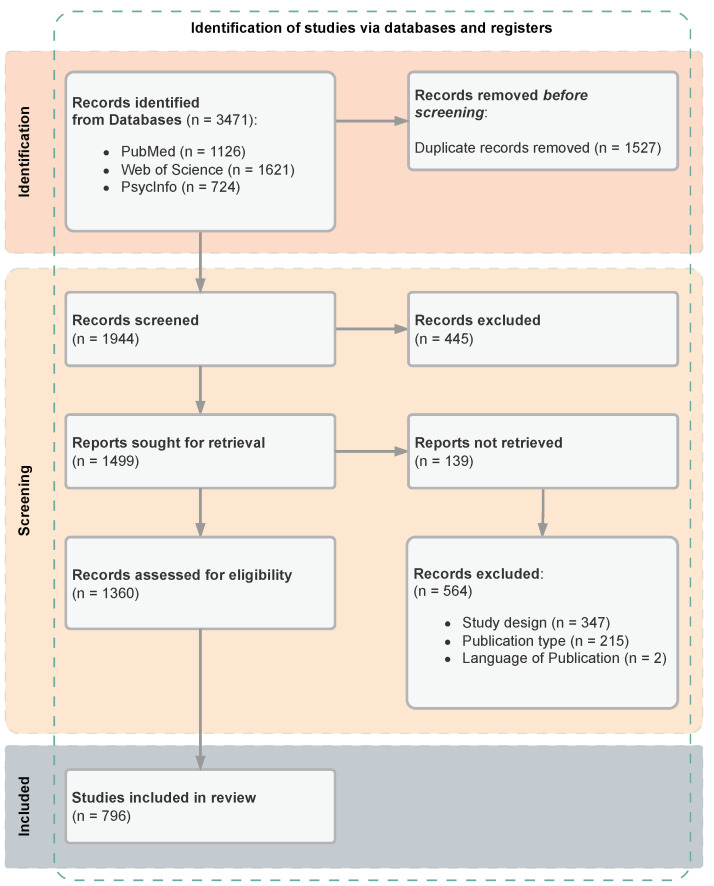
PRISMA 2020 [3] flow diagram of the publication selection and screening process.

In terms of *data management*, we used the Rayyan (2022) platform (https://www.rayyan.ai/, accessed on 31 January 2023) for duplicate filtering and assisting the review process. Rayyan records which publications were excluded from the further review process, along with a reason for the exclusion of any single publication. After we removed duplicates from our literature list, we conducted the screening of the remaining literature. For the *selection process*, in the first round of screening, we decided whether to include or exclude the records for further processing based on the title, abstract, and research area of each publication. Second, we sought to retrieve the remaining records and excluded records that could not be retrieved. Third, in the second round of screening, the titles and abstracts of the remaining publications were screened in more detail, and publications were included or excluded based on the eligibility criteria defined. In the cases where we excluded an entry, we provided a short reasoning (e.g., wrong research field, no EMA was conducted). To make this process transparent, we used a flowchart based on the template provided by PRISMA for visualization (see Figure 1). For the *data collection process*, we extracted the information from the full texts of the included papers into a spreadsheet.

To minimize errors and reduce introduction of potential biases by data extractors [23], two independent reviewers from complementary disciplines (i.e., computer science and psychology) extracted the data from the eligible reports. In this process, the first reviewer collected all data, and the second reviewer confirmed and potentially corrected the information through random sampling of the reports in the spreadsheet (i.e., every 25th record in a list ordered by the authors). Any discrepancies were resolved through discussion with the authors, who then reviewed the corresponding reports in order to decide on the further action to be taken and any necessary adjustments to the extraction process. In addition, suspicious values and outliers detected during the analysis were double-checked and systematically corrected by a third reviewer from the authors. The data extraction form was structured as outlined in the following subsections.

#### 4.4.1. General Characteristics

We assigned each publication analyzed to an overarching research topic and grouped the publications based on these topics. Furthermore, we extracted title, keywords, and the year of publication. In addition, the number of participants, their gender, and their age were recorded.

#### 4.4.2. Quality

Although we did not directly assess the quality of the literature reviewed, we examined the literature with respect to statements about a risk of bias. In the analysis, we assessed the proportion of studies that take into account such a risk of bias. Also of interest was what type of bias was taken into account by the authors. A publication was considered to take into account a specific risk of bias if that risk was mentioned at least once in the report.

Similar to the review of risk of bias, we also assessed whether the authors of the reviewed literature take into account safety and privacy issues for the EMA study design. Given the use of sensors for data collection, this may impact participant trust. A publication was considered to take into account security or privacy if it was mentioned at least once in the report.

#### 4.4.3. EMA Characteristics

One major aspect that can be influenced by a suitable EMA study is the adherence of individual participants to follow the study design. We therefore collected the relative number of prompts answered by participants for each publication. As one aspect expected to affect adherence [18], it was recorded whether feedback was provided to participants. The feedback can thereby be provided either manually (e.g., by a healthcare provider) or automatically by the system. In the same context, it was recorded whether participants received guidance by a healthcare provider during data collection. Another aspect associated with higher adherence rates in the literature is the provision of monetary compensation to participants [17,18]. Thus, we recorded whether or not such compensation was provided. In addition, it was recorded whether participants were provided with a smartphone by the investigators to complete the EMA prompts or were instructed to use their own (i.e., *user-owned*) smartphone. Adherence was assumed to increase when participants were allowed to use their own smartphone and therefore only had to carry a single device.

In Section 2, we defined archetypes based on the sampling strategy (e.g., event-contingent) and the concrete assessment procedure (e.g., self-assessment). Based on this information, we extracted the primary EMA/MCS strategies. With respect to the prompting strategy and EMA transmission, EMA surveys can be communicated to participants in a variety of ways. We extracted information about whether a fixed-time, random, or semi-random prompting strategy was used and whether a smartphone application, text message, or website was used as communication channel. In addition, information on EMA study design characteristics was extracted, such as the number of EMA prompts per day, the number of total days of study duration, and the number of questions per EMA prompt. In this process, we explicitly labeled studies that used only a single question per prompt, which are referred to as *microinteraction-based Ecological Momentary Assessments* (micro-EMAs or μEMAs) [24].

#### 4.4.4. Sensor Usage

In reviewing the literature, we distinguished between publications that use EMA only and publications that additionally use sensors. In addition, we obtained information on which sensors were used in the EMA study. Here, we extracted which sensors are used in these EMA studies. Because we analyzed which sensors were used in the EMA studies, we were also interested in the use of these sensors. In this process, we extracted whether the sensors were reported in the study report, were used as triggers for an EMA survey, or were used to verify the responses of the EMA study participants.

### 4.5. Data Analysis

All data were analyzed using *Python 3.9.12*, with libraries *pandas 1.4.2*, *NumPy 1.21.5*, *SciPy 1.7.3*, *statsmodels 0.13.2*, and *pingouin 0.5.2*. The libraries *Matplotlib 3.5.1* and *seaborn 0.11.2* were used for data visualizations.

With respect to descriptive statistics, for categorical variables, counts and proportions in relation to the total sample size (i.e., percentages) were determined. For continuous variables, means, standard deviations (SDs), medians, minimums, and maximums were calculated.

To analyze the distribution of different combinations of EMA and MCS strategies, contingency tables of counts and percentages were created for both the entire dataset and separately for publications without sensor usage (EMA only) and publications with sensor usage (EMA and MCS). Multiple $\chi^{2}$ tests were used to examine differences between “EMA only” and “EMA and MCS” studies with respect to the distributions of assessment types, sampling strategies, prompting strategies, and EMA transmission.

Regarding sensor usage, the number of publications in which each sensor was used and the distribution of this usage were calculated. In addition, the distribution of publications using sensor values as data points (i.e., reported the sensor data), for verification, and as a trigger was determined.

In terms of adherence to the study design, we analyzed the publications in two parts. First, we examined the dataset using hypotheses based on the EMA literature and general assumptions about EMA studies. Second, we conducted an exploratory analysis to determine whether there are other study conditions that influence adherence. Regarding the latter, we investigated the influence of the sampling strategy used, the EMA transmission channel, the use of sensors in the study design, and the use of a μEMA approach. The hypotheses for the hypotheses-based part are described in the following:

**Hypothesis 1** (H1)**.** *Studies that incorporate feedback achieve higher adherence than studies that do not.*

**Hypothesis 2** (H2)**.** *Studies that incorporate guidance by a healthcare provider achieve higher adherence than studies that do not.*

**Hypothesis 3** (H3)**.** *Studies that provide a monetary compensation achieve higher adherence than studies that do not [17,18].*

**Hypothesis 4** (H4)**.** *Studies that let participants use their own smartphone (i.e., user-owned) to contribute data achieve higher adherence than studies in which a provided smartphone has to be used.*

**Hypothesis 5** (H5)**.** *Studies that incorporate a fixed prompting strategy achieve higher adherence than studies that use a random or semi-random sampling strategy [17].*

**Hypothesis 6** (H6)**.** *The adherence rate decreases with an increasing number of EMA prompts per day [17,18].*

**Hypothesis 7** (H7)**.** *The adherence rate decreases with an increasing study duration.*

**Hypothesis 8** (H8)**.** *Combining H6 and H7, the adherence rate decreases with an increasing total number of prompts (prompts per day × study duration).*

**Hypothesis 9** (H9)**.** *The adherence rate decreases with an increasing number of questions per EMA prompt.*

**Hypothesis 10** (H10)**.** *Combining H8 and H9, the adherence rate decreases with an increasing total number of questions (total number of prompts × number of questions per EMA prompt).*

In this process, for categorical variables, a one-way ANOVA was performed, using *Tukey’s HSD* test as a post hoc test. For continuous variables, a Pearson correlation coefficient was computed. Due to the exploratory nature of this analysis, both raw *p*-values and corrected *p*-values according to the method of Benjamini and Hochberg [25] are reported.

## 5. Results

The publication selection and screening process is illustrated in Figure 1. The database search returned a total of 3471 publications. After removing all duplicate results (n=1527), 1944 records remained. The titles and abstracts of these publications were then initially screened, excluding 445 papers from research areas that did not meet our inclusion criteria (e.g., physics). Of the remaining 1499 records, 139 additional records had to be excluded that could not be retrieved (e.g., because they were locked behind a paywall or not yet published). In the second round of screening, the titles and abstracts of the remaining 1360 records were then assessed for eligibility and 564 records were excluded based on the defined inclusion and exclusion criteria. In this process, 347 publications were excluded due to study design (e.g., no EMA approach), 215 due to publication type (e.g., review), and 2 based on the publication language. This process resulted in 796 publication full texts included for the analysis at hand. The complete list of references included can be found in the Supplementary Materials.

### 5.1. Research Topics and EMA Use over Time

**Figure 2:**
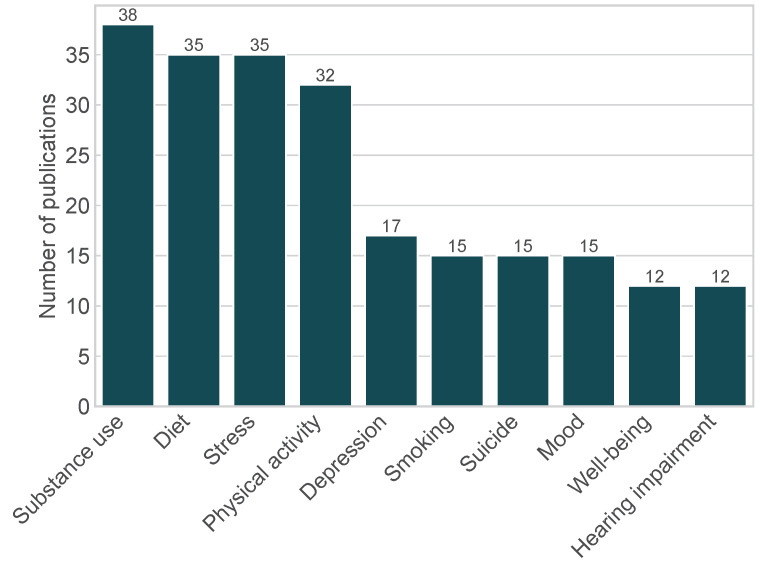
Top 10 research topics in the EMA publications analyzed (n=796).

**Figure 3:**
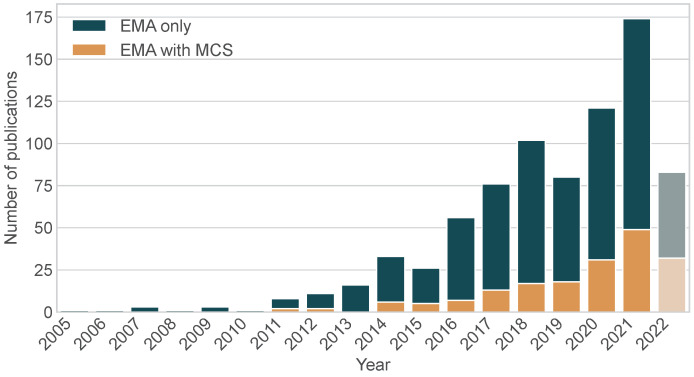
Number of EMA publications utilizing mobile (crowd) sensing published per year between 2005 and 2022. Year 2022 is displayed grayed out, as it was not yet completed. The dark green bar represents the number of EMA studies that relied solely on EMA without any sensor usage. The orange bar represents the number of EMA studies that additionally collected sensor readings as data from MCS devices (n=796).

The predominant research topics among the 796 publications analyzed are shown in Figure 2. Other more frequently investigated research topics include emotions, anxiety, cognition, and HIV. Furthermore, COVID-19 emerged as a topic in EMA mHealth research from 2020. In addition to examining the research topics, we also examined the distribution of EMA use over time (see Figure 3). With the exception of 2019, an overall upward trend in the number of EMA studies can be observed. The number of EMA studies that include EMA only is generally higher than the number of studies that use both EMA and MCS. However, our results show that over time, an increasing number of studies tend to include sensor data in EMA research.

### 5.2. Descriptive Statistics

In the following, more detailed results are presented alongside the aspects presented in Section 4 (see Table 1). The total number of participants combined across all included studies is 2,911,429, with an average of 6221 participants (SD=131,400.1) per study (61.0% female, 38.7% male). The median number of participants is 61, with a lower quartile (Q1) of 29 participants and an upper quartile (Q3) of 121.25 participants per study. Note that one of the studies reported a particularly large number of 2,842,732 participants [26], which skews the mean and SD because this number is so far from the median. The mean age across all studies was 35.7 years (SD=16.4).

We further examined to what extent any risk of bias was considered by the authors. Of all studies reviewed, more than half, 55.8%, considered any form of risk of bias in the course of the individual EMA study and mentioned these risks in the report. Recall bias was mentioned most frequently, in 45.1% of the cases. Selection bias was considered in 13.9% of the literature reviewed and reporting bias was mentioned in 5.0% of the cases. In the literature, we found that only 17.3% of all studies considered security aspects in their study design. Slightly fewer studies considered privacy aspects (16.8%). Because MCS sensors can have privacy implications and EMA participants may disclose intrusive private information, it may be important to ensure that this information remains secure and private, as it is possibly a critical factor in motivating potential participants to participate in a study.

With respect to EMA characteristics, sensors were used in a in total of 23.2% of the publications analyzed. Sensor usage is examined in more detail in Section 5.4. The mean adherence rate across all studies examined is 72.0% (SD=17.0%). Adherence in relation to various EMA characteristics is examined in more detail in Section 5.5. A total of 123 (15.5%) studies provided feedback to their participants as part of the EMA approach. Thereby, automated feedback was used considerably more frequently (12.4%), in contrast to manual feedback (3.0%). Most of the studies analyzed (84.0%) were unguided, with only 122 (15.3%) of the studies providing guidance by a healthcare provided during data collection for participants. Monetary compensation was provided to participants in 54.5% of the studies in the publications analyzed. In 69.5% of the studies, participants were instructed to use their own (i.e., *user-owned*) smartphone, whereas in 28.6% of the studies, participants were provided with a smartphone by the investigators to complete the EMA prompts. The mean number of EMA prompts presented to participants across all studies was 5.0 (SD=4.8), with a mean study duration of 27.3 (SD=50.6) days and a mean number of 9.8 (SD=8.7) questions per prompt. In 12 (1.5%) studies of the publications analyzed, a micro-EMA (μEMA) approach with only a single question was used.

### 5.3. EMA and MCS Strategies

Regarding the different strategies used for assessment, sampling, and prompting in EMA and MCS studies, we found the results shown in Table 2, Table 3, Table 4 and Table 5. We first focus on the participatory assessment strategy in self-assessments, which are used in 81.4% of the studies, as shown in Table 2. In this category, we observed this assessment strategy most frequently combined with the signal-contingent sampling strategy in 47.1%. This is followed by the combination with the interval-contingent sampling strategy in 25.6% of the reviewed cases. The event-contingent sampling strategy is used only in 8.7%. The remaining sampling strategies were not used in combination with participatory self-assessment. For the surveillance assessment strategy, there are two options for creating an EMA design. In the literature evaluated, where the surveillance strategy was selected, four studies (0.5%) combined it with event-contingent sampling. The signal, interval, and continuous sampling strategies were combined with the surveillance strategy each in one (0.1%) of all cases examined. The context-sensitive sampling strategy was not used in combination with the surveillance strategy. Finally, we examined combinations with the automated opportunistic assessment strategy, which was used in 17.7% of the cases. In the entire group of literature reviewed, the signal-contingent sampling strategy was used in 8.5% of automated assessments. This is followed by the interval-contingent sampling strategy in 7.8% of the cases. The event-contingent sampling strategy was used in 1.3% of all cases studied and the context-sensitive sampling strategy in 0.1%. The continuous sampling strategy was not used in combination with the automatic opportunistic assessment strategy.

When only considering the EMA publications utilizing MCS (see Table 3), it can be seen that the automated assessment type is the most frequently used primary type with 138 (74.6%) publications. Interestingly, these 138 publications constitute 97.9% of the 141 publications with the automated assessment type in the overall dataset. Conversely, self-assessment is used in only 22.2% and surveillance assessment in only 3.2% of the studies analyzed. When comparing EMA publications with sensor usage with publications without sensor usage (see Table 4), a $\chi^{2}$ test confirmed that the distribution of assessment types is significantly different ($\chi^{2}=559.5$, p<0.001). With respect to sampling strategies, generally the distribution is similar to that in the overall dataset. However, the interval-contingent sampling strategy is used about 5% more and the signal-contingent strategy about as much less than in the overall dataset. When comparing EMA publications with sensor usage with publications without sensor usage (see Table 4), a $\chi^{2}$ test confirmed that the distribution of sampling strategies is significantly different ($\chi^{2}=10.4$, p=0.035).

Regarding the prompting strategy, as shown in Table 5, most prompts were delivered using fixed-time notifications (39.9%), followed by random (30.5%) and semi-random notifications (27.1%). When comparing EMA publications with sensor usage with publications without sensor usage, no significant differences in prompting strategies can be observed. With respect to the transmission of EMA surveys to study participants, a smartphone application was used in 88.1%, followed by short message service (SMS) as a communication channel in 9.7% of the publications studied. The remaining studies used a website (0.9%) or phone call (0.1%) to communicate with participants. Once again, no significant differences in terms of the communication channel used to transmit EMA can be observed when comparing EMA publications with sensor usage with publications without sensor usage.

### 5.4. Sensor Usage

**Figure 4:**
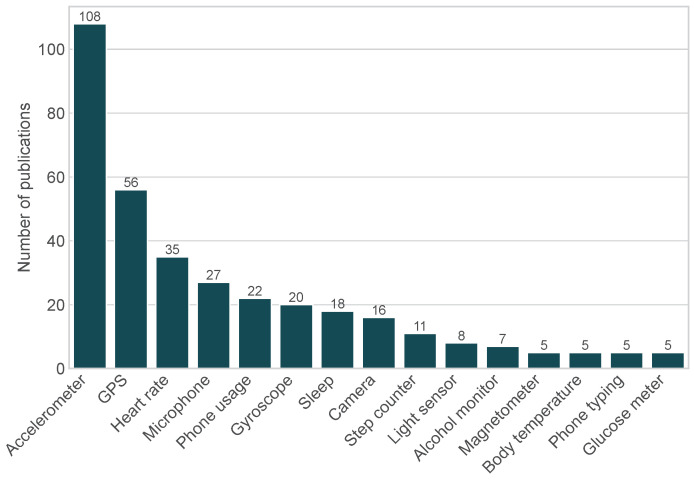
Sensors used in EMA-MCS publications analyzed. Only the usage of each individual sensor is displayed, regardless of whether it has been combined with another sensor (n=185).

Overall, 185 (23.2%) of the publications analyzed used EMA with sensor readings (see Table 1). For these EMA-MCS studies, we analyzed the distribution of sensors and the use of these sensor data. First, the distribution of sensors predominantly used is shown in Figure 4. The most commonly used sensor is the accelerometer in 108 (58.4%) individual studies. Next, the GPS sensor is used in 56 (30.3%) studies. This is followed in descending number of articles of reviewed literature by the heart rate monitor and microphone, which were used in 35 (18.9%) and 27 (14.6%) studies, respectively.

**Figure 5:**
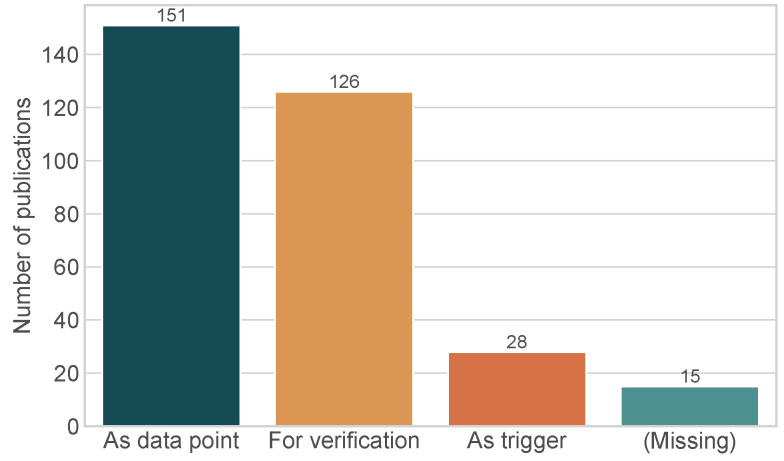
Use of sensors in EMA-MCS publications analyzed (n=185).

To better understand the role of sensors, we divided our analysis of sensor use in EMA studies into three categories: The first category of EMA studies presents sensor data in their study report and uses the data as data points alongside the EMA data. The next two categories are (1) the sensor data are used as triggers (e.g., to transmit an EMA survey) or (2) the sensor data are used to verify the answers participants provided in their EMA surveys. Details about the distribution of categories are shown in Figure 5. We found that 170 (91.9%) of all EMA studies that incorporated a sensor into their study design reported how the recorded sensor data were used. The remaining 15 (8.1%) of EMA-MCS studies used sensor data as part of the study, but did not report any numbers in the study report or indicate how the sensor data were further used. Of the EMA studies that provided details on the use of sensor values, only 28 (15.1%) used sensor data as triggers. In contrast, 151 (81.6%) reported the recorded sensor data in the publication and 126 (68.1%) of studies that utilized sensors, used these sensor readings to verify study participant responses.

### 5.5. Adherence

**Figure 6:**
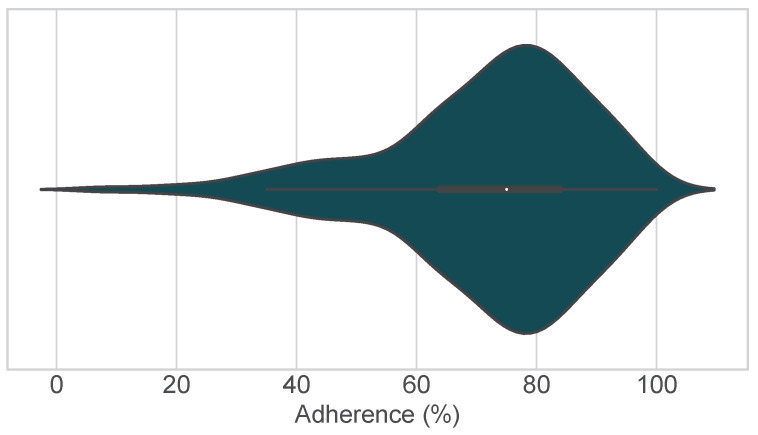
Violin plot of the distribution of adherence rates in the publications analyzed (n=558).

The adherence rate was available for 558 (70.1%) of the publications analyzed. The distribution of the adherence rates is shown in Figure 6. It can be seen that most studies reported an adherence rate between 64.0% (Q1) and 83.7% (Q3), with a median adherence rate (Q2) of 75.0%. Only 61 (10.9%) of the publications reported adherence rates below 50%.

Significant differences with respect to the total number of prompts (H8), studies with sensor usage, and studies using a micro-EMA (μEMA) approach were found in the analysis of adherence rates comparing the different study conditions. As shown in Table 6, in the hypotheses-based part, neither feedback (H1), guidance (H2), monetary compensation (H3), smartphone owned/provided (H4), prompting strategy (H5), prompts per day (H6), study duration (H7), number of questions per prompt (H9), or total number of questions (H10) had a significant effect on the adherence rate. However, the adherence rate was significantly negatively correlated with the total number of prompts (r=−0.115,p=0.009), confirming our hypothesis H8.

**Figure 7:**
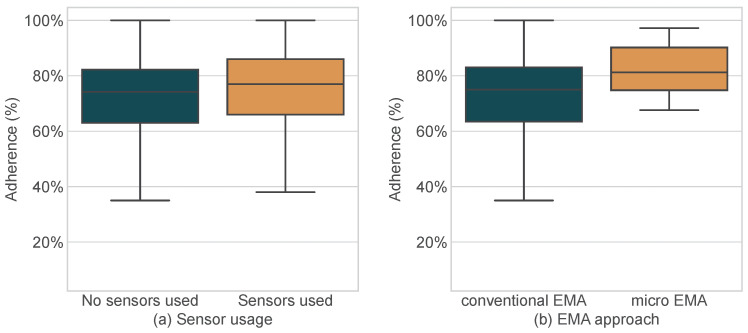
Adherence rate distributes compared between (**a**) publications without and with sensor usage and (**b**) publications with a conventional EMA approach and a micro-EMA approach.

In the exploratory part, the EMA transmission channel had no significant effect on the adherence rate. The sampling strategy initially showed a small significant effect on the adherence rate ($F=2.733,\eta^{2}=0.01,p=0.043$). However, no significant pairwise differences were found using Tukey’s HSD test. Importantly, significantly higher adherence rates were observed for studies with sensor usage compared to EMA studies without any sensors used ($F=4.046,\eta^{2}=0.01,p=0.045$) and studies using a μEMA approach ($F=4.019,\eta^{2}=0.01,p=0.045$). The corresponding different distributions of adherence rates are shown in Figure 7. Note that, when correcting for multiple comparisons using the Benjamini–Hochberg method, none of the differences between study conditions remained significant. For this reason, the results should be interpreted with appropriate caution.

## 6. Discussion

### 6.1. Research Topics and EMA/MCS Use over Time

We have revealed in our analysis that mHealth EMA research has mainly focused on areas that are universally applicable (e.g., diet, stress management, and physical activity). However, we also saw research in more pressing areas such as using EMA and MCS to examine substance use (e.g., alcohol or drug addiction), depression, and anxiety.

In general, our analysis showed that the amount of mHealth EMA research is continuously growing, while it also becomes evident that the combination of EMA with MCS provides substance for further research enabling a variety of study designs. In Figure 3, the graph drops around 2019. While the number of studies using only EMA with prompts declined that year, the number of studies that also used MCS kept growing. The reason for this sudden decline could be linked to global causes (e.g., the COVID-19 pandemic), but further investigation would be necessary in order to investigate the cause of the decrease.

### 6.2. EMA and MCS Strategies

Mobile apps have become the predominant (88.1%) communication channel for transmitting EMA to participants, both for conventional EMA studies and studies combining EMA and MCS. It is therefore not surprising that researchers are increasingly taking advantage of the additional capabilities of smart mobile devices (e.g., smartphones), including advanced processing capabilities and built-in as well as locally connected sensors [2]. With respect to the prompting strategy, fixed-time prompts are the the most prominent strategy. This might be the case because this prompting strategy is associated with less disruption and therefore higher adherence rates [17], as well as better technical stability and reliability [2]. However, a fixed prompting strategy allows participants to integrate answering the questionnaire into their daily routine, which could reduce ecological validity and increase bias [2,17,27].

It has been shown that the primary assessment strategy in mHealth EMA studies in general (81.4%) and particularly in conventional EMA research (99.3%) is self-assessment. In contrast, for EMA studies utilizing MCS, the main primary assessment strategy is automated assessment (74.6%). These more recent studies appear to make extensive use of the technical capabilities of smart mobile devices, which have already been widely used to transmit EMA. However, for both conventional EMA studies and EMA-MCS studies, the predominant sampling strategies are signal-contingent (57.8% and 49.2%) and interval-contingent (32.1% and 38.4%) sampling. We argue that the technological capabilities of smart mobile devices enable the use of even more automated, event-based and context-aware sampling and assessment strategies that are still rather rarely used.

### 6.3. Sensors, Security, and Privacy

Overall, one in five analyzed publications (23.2%) used EMA with sensor readings in its study design. As shown in Figure 3, this number has continued to increase over the past decade, indicating a further increase in the coming years and highlighting the importance of this combination for future EMA research. This percentage is also higher than the 12% found in the review by Wrzus et al. [18] in 2020. This trend of increasing sensor usage could also be linked to the emergence of other related advances such as the Artificial Intelligence of Things (AIoT), which combines the Internet of Things (IoT) with advanced Artificial Intelligence (AI) technologies [28]. In healthcare, AIoT is considered to drive the paradigm shift towards Healthcare 5.0 by leveraging the capabilities of smart sensors [29,30].

The most commonly used sensors in the identified EMA-MCS studies are sensors that are frequently used for general activity recognition (e.g., accelerometer) and localization (e.g., GPS). However, more directly health-related sensors such as heart rate, alcohol, glucose, and body temperature monitors can be increasingly found in the literature.

In the context of EMA studies that utilized sensors, we revealed that 68.1% of studies that applied sensors also used the data for verification of the EMA responses. In contrast, we saw that only 15.1% of studies used sensors as triggers to automate EMA prompts in a context-aware manner. Research has already shown that with the help of three sensors (accelerometer, light sensor, and phone usage/locked state), it is fairly accurate to assess whether the participant is sleeping [31]. Further research in the use of a more intelligent prompting with the help of machine learning algorithms can have positive effects on adherence and engagement of participants by prompting in convenient moments [32]. Today’s sensors, especially wearable sensors, show good classification results of behavior features. With the help of these sensors, it is fairly accurate to identify, among others, stressful situations and sleep state [33].

While the use of sensors can provide benefits in research, additional factors need to be considered. These factors include the consideration of security and privacy aspects in the implementation and deployment of EMA studies and MCS devices/applications. As mHealth involves the collection and processing of potentially highly sensitive health-related and personal data, the protection of these data is of the utmost importance. Legal regulations such as the General Data Protection Regulation (GDPR) in the EU and the Health Insurance Portability and Accountability Act (HIPAA) in the USA even require this protection by law. However, as we saw in our analysis, these aspects are currently not always considered, as only about 17% of the publications analyzed stated any consideration of these aspects. The lack of this consideration may also have an impact on the adherence and dropout rates in EMA-MCS studies, since more extensive use of MCS technology requires corresponding acceptance and trust of study participants [34]. These concerns are appropriate under the circumstance that the accelerometer is already sufficient to infer intimate information on well-being [35]. Interestingly, this is also the most commonly used sensor in the studies examined. Approaches such as privacy-preserving computing [36] should be utilized to minimize privacy threats and maximize participant trust in the context of mHealth and eHealth in general [37].

Finally, 8.1% of the publications examined that utilized sensors did not provide any information how these sensor data were further used. In general, the reporting on sensor usage varied substantially across the publications analyzed. We propose that researchers should follow and establish standardized protocols for reporting EMA-MCS studies, similar to the WHO mERA checklist [20] or the CONSORT statement [38].

### 6.4. Adherence

The average adherence rate to the study protocol across all publications included in this analysis was 72.0%. This number is comparable but slightly lower than that found in the analyses by Vachon et al. (78.7% [17]) and Wrzus et al. (79.2% [18]). In addition, the variation in the adherence rates observed was higher than in the review of Wrzus et al. (SD: 17.0% vs. 13.6% [18]). It remains unclear whether this lower average value and higher variation are due to the more recent literature, the larger number or broader scope of publications included, the specific databases and search terms used, or other characteristics.

Contrary to our hypotheses H1–H7, H9, and H10, no significant differences in terms of the adherence rate were observed for feedback, guidance, monetary compensation, smartphone owned/provided, prompting strategy, prompts per day, study duration, number of questions per prompt, and total number of questions. This is partially in line with prior findings of Wrzus et al. regarding feedback, guidance, and study duration [18], and with prior findings of Vachon et al. regarding study duration and number of questions per prompt [17]. However, these analyses did observe effects for monetary compensation [17,18], prompting strategy [17], and prompts per day [17,18] that could not be confirmed in the present study. We were also unable to confirm the findings of van Berkel et al. [39], who found an effect of the sampling strategy on adherence rates in a within-subjects study. In contrast, we found a significant negative relationship between adherence rates and the total number of scheduled prompts (H9). This is in contradiction to the results of Wrzus et al. [18]. Overall, however, these studies agree that there appears to be *some* effect of the number of scheduled prompts on adherence rates. The interaction between prompts per day and study duration should be further investigated. In this context, special consideration should be given to an EMA approach coined by Intille et al., which is referred to as a microinteraction-based Ecological Momentary Assessment (micro-EMA or μEMA) [24]. This approach reduces EMA to quick, at-a-glance interactions with single questions that can be answered within seconds, and can be implemented well with, for example, smartwatches [24]. The authors have shown in comparative studies that significantly higher adherence rates can be achieved with μEMA than with conventional EMA [24,40]. Although we observed only a relatively small number of μEMA studies (1.5%), in our meta-analysis we found significantly higher adherence rates in these studies compared to the remaining studies, confirming the findings of Intille et al. Thus, even μEMA studies with up to 72 prompts per day [41] or 36 weeks study duration [42] achieved high adherence rates of 74–97%. In consequence, μEMA may be used to collect self-report measurements with high temporal density while reducing the burden on participants [40].

Interestingly, we found significantly higher adherence rates for studies with sensor usage compared to conventional EMA studies without any sensors used. Since this effect is small, it may be purely a consequence of the predominantly more recent studies and an overall more mature research field of EMA and its design (e.g., researchers have recognized the importance of adherence, participant burden, and incentives) [43,44,45]. However, the effect may also be explained by the participants’ preference for technologically more advanced mHealth apps that use modern technologies such as sensors. Further research is needed to investigate the underlying causes of this difference.

### 6.5. Limitations

The large number of studies included and the broad focus is both a strength and a weakness of this systematic literature review. While we were able to gain a good overview of the mHealth EMA research field and various aspects (e.g., sensors and adherence), the sheer volume of publications leaves much room for error in the search and extraction process. First, the selection of information sources and keywords in the search strategy has a strong influence on the final dataset of included literature. Other databases and other keywords could lead to a different set of publications, and consequently, different results. Second, in the selection process, we included publications based on the titles and abstracts of each record. Publications that did not include all information regarding our eligibility criteria in their abstract may have been incorrectly excluded. Another limitation is that some publications were not accessible and had to be excluded from further analysis. These additional sources could have potentially provided further insights. Third, although we have implemented procedures to minimize errors and reduce the introduction of potential biases by data extractors (see Section 4.4), it still cannot be ruled out that some fields were not correctly extracted from the publications. In addition, our review protocol allowed only a single entry for some extracted fields, whereas a single study design, for example, may use multiple sampling strategies (in these cases, we extracted only the primary strategy).

Moreover, we were still only be able to focus on very specific and narrow areas for extraction and analysis. This type of review may provide useful insights, but may also leave questions unanswered due to the broad focus (e.g., the specific usage of sensors in each study or the interactions of specific study design characteristics on adherence). These types of questions would require more detailed analyses and either an even larger body of literature or a focus on a very specific subset of it.

## 7. Conclusions

In this work, we reviewed a large number of literature sources dealing with EMA, MCS, and their combination in mHealth. Extracting relevant information from the reviewed publications gave us the opportunity to analyze the current status and trends in this research area. Our analysis showed that both EMA research in general and the use of sensors in EMA research are steadily increasing. We have shown that mobile apps have become the predominant method of delivering EMA to mHealth study participants. However, traditional signal-contingent or interval-contingent self-assessment remain the most common strategies in these EMA studies, although technological capabilities would allow for more automated approaches. Furthermore, although more and more sensors are being used in EMA studies, it appears that their potential is rarely exploited beyond mere data collection. For example, very few studies have used sensors as triggers to automate EMA prompts in a context-aware manner. Overall, reporting of sensor usage in EMA studies lacks transparency and standardization. In addition, security and privacy aspects are addressed in only a subset of mHealth EMA publications, although their importance is increasing, especially as the use of sensors continues to grow. Moreover, we found factors that impact the adherence rates of EMA studies. These characteristics include the total number of prompts over the course of a study, which is influenced by the study duration and the number of prompts per day. Novel approaches such as μEMA already take these aspects into account and indicate results with higher adherence rates. We also found indications of higher adherence rates in EMA studies with sensor usage, which is an area that needs further research. Given the ongoing research and further publications on this topic, there is a need to update the data extracted in this work. We envision that this will provide further insight into developments and serve as a basis for future EMA/MCS studies.

## Figures and Tables

**Table 1:** Descriptive statistics of publications analyzed (n=796).

Category	Characteristic	Value
General		
Number of participants	Total	2,911,429 (100%)
	Mean (SD)	6221.0 (131,400.1)
	Median [Min, Max]	61.0 [2, 2,842,732]
Gender	Female	1,775,054 (61.0%)
	Male	1,127,585 (38.7%)
	(Missing)	8790 (0.3%)
Age	Mean age (SD)	35.7 (16.4)
Quality		
Risk of bias considered	Total	444 (55.8%)
	Recall bias	359 (45.1%)
	Selection bias	111 (13.9%)
	Reporting bias	40 (5.0%)
Security and Privacy	Security considered	138 (17.3%)
	Privacy considered	134 (16.8%)
EMA characteristics		
Sensor usage	No sensors used	611 (76.8%)
	Sensors used	185 (23.2%)
Adherence	Mean adherence rate (SD)	72.0 (17.0%)
Feedback	Total	123 (15.5%)
	Automated	99 (12.4%)
	Manual	24 (3.0%)
Guidance	Guided	122 (15.3%)
	Unguided	669 (84.0%)
	(Missing)	5 (0.6%)
Monetary compensation	Provided	434 (54.5%)
Smartphone	User-owned	553 (69.5%)
	Provided	228 (28.6%)
	(Missing/No smartphone)	15 (1.9%)
Prompts per day	Mean (SD)	5.0 (4.8)
	Median [Min; Max]	4.0 [0.1; 72.0]
Days of study duration	Mean (SD)	27.3 (50.6)
	Median [Min; Max]	14.0 [0.5; 435.0]
Number of questions per prompt	Mean (SD)	9.8 (8.7)
	Median [Min; Max]	7.0 [1.0; 56.0]
	Micro-EMA (μEMA)	12 (1.5%)

**Table 2:** Combinations of primary assessment types and sampling strategies for all analyzed publications (n=796).

Assessment	Self-Assessment	Surveillance	Automated	All
**Sampling**				
Interval	204 (25.6%)	1 (0.1%)	62 (7.8%)	267 (33.5%)
Signal	375 (47.1%)	1 (0.1%)	68 (8.5%)	444 (55.8%)
Event	69 (8.7%)	4 (0.5%)	10 (1.3%)	83 (10.4%)
Context	0	0	1 (0.1%)	1 (0.1%)
Continuous	0	1 (0.1%)	0	1 (0.1%)
All	648 (81.4%)	7 (0.9%)	141 (17.7%)	796 (100%)

**Table 3:** Combinations of primary assessment types and sampling strategies for EMA publications utilizing MCS (n=185).

Assessment	Self-Assessment	Surveillance	Automated	All
**Sampling**				
Interval	10 (5.4%)	1 (0.5%)	60 (32.4%)	71 (38.4%)
Signal	24 (13.0%)	0	67 (36.2%)	91 (49.2%)
Event	7 (3.8%)	4 (2.2%)	10 (5.4%)	21 (11.4%)
Context	0	0	1 (0.5%)	1 (0.5%)
Continuous	0	1 (0.5%)	0	1 (0.5%)
All	41 (22.2%)	6 (3.2%)	138 (74.6%)	185 (100%)

**Table 4:** Primary assessment types and sampling strategies in a comparison between EMA publications without (EMA only) and publications with (EMA and MCS) sensor usage (n=796).

Characteristic	All (n=796)	EMA Only (n=611)	EMA and MCS (n=185)	Test Statistic (DoF)	*p*-Value
**Assessment types**				$\chi^{2}=559.5$(2)	<0.001
Self-assessment	648 (81.4%)	607 (99.3%)	41 (22.2%)		
Surveillance	7 (0.9%)	1 (0.2%)	6 (3.2%)		
Automated	141 (17.7%)	3 (0.5%)	138 (74.6%)		
**Sampling strategy**				$\chi^{2}=10.4$(4)	0.035
Interval	267 (33.5%)	196 (32.1%)	71 (38.4%)		
Signal	444 (55.8%)	353 (57.8%)	91 (49.2%)		
Event	83 (10.4%)	62 (10.1%)	21 (11.4%)		
Context	1 (0.1%)	0 (0.0%)	1 (0.5%)		
Continuous	1 (0.1%)	0 (0.0%)	1 (0.5%)		

**Table 5:** Prompting strategies and EMA transmission in a comparison between EMA publications without (EMA only) and publications with (EMA and MCS) sensor usage (n=796).

Characteristic	All (n=796)	EMA Only (n=611)	EMA and MCS (n=185)	Test Statistic (DoF)	*p*-Value
**Prompting strategy**				$\chi^{2}=5.5$(2)	0.063
Fixed	318 (39.9%)	234 (39.5%)	84 (45.4%)		
Random	243 (30.5%)	181 (30.6%)	62 (33.5%)		
Semi-random	216 (27.1%)	177 (29.9%)	39 (21.1%)		
(Missing)	19 (2.4%)	19 (3.1%)	0		
**EMA transmission**				$\chi^{2}=2.6$(4)	0.620
App	701 (88.1%)	538 (88.1%)	163 (88.1%)		
SMS	77 (9.7%)	62 (10.1%)	15 (8.1%)		
Website	7 (0.9%)	4 (0.7%)	3 (1.6%)		
Phone call	1 (0.1%)	1 (0.2%)	0		
(Missing)	11 (1.4%)	7 (1.1%)	4 (2.2%)		

Note that in one study, both app and SMS were used to transmit EMAs.

**Table 6:** Mean adherence rate compared between different study conditions (n=558).

Characteristic	Mean Adherence Rate (SD)	Test Statistic (DF)	*p*	$p_{\mathrm{adjusted}}$
Hypothesis-based part				
**Feedback**		F = 0.107 (1)	0.743	0.813
Yes	71.4% (17.9%)			
No	72.1% (16.9%)			
**Guidance**		F = 0.274 (1)	0.601	0.813
Guided	71.1% (18.0%)			
Unguided	72.1% (16.8%)			
**Monetary compensation**		F = 1.181 (1)	0.278	0.432
Yes	71.3% (17.8%)			
No	72.9% (15.9%)			
**Smartphone**		F = 1.429 (1)	0.233	0.407
User-owned	71.4% (17.2%)			
Provided	73.2% (16.8%)			
**Prompting strategy**		F = 0.281 (2)	0.755	0.813
Fixed	72.6% (17.9%)			
Random	71.3% (17.2%)			
Semi-random	71.6% (15.9%)			
**Prompts per day**		r = −0.016 (518)	0.712	0.813
2.0 (Q1) or less	71.8% (18.0%)			
6.0 (Q3) or more	71.7% (16.6%)			
**Study duration (days)**		r = −0.074 (542) *	0.085	0.238
7 (Q1) or less	72.7% (17.8%)			
28 (Q3) or more	71.4% (17.3%)			
**Total number of prompts**		r = −0.115 (511) ***	0.009	0.129
28 (Q1) or less	72.8% (18.1%)			
90 (Q3) or more	70.0% (16.8%)			
**Number of questions per prompt**		r = 0.015 (123)	0.864	0.864
4 (Q1) or less	77.0% (13.1%)			
13 (Q3) or more	73.8% (13.9%)			
**Total number of questions**		r = −0.134 (112)	0.155	0.310
180 (Q1) or less	77.6% (10.8%)			
840 (Q3) or more	70.3% (16.2%)			
Exploratory part				
**Sampling strategy**		F = 2.733 (3) **	0.043	0.159
Interval	74.0% (16.1%)			
Signal	71.0% (17.1%)			
Event	70.3% (20.0%)			
**EMA transmission**		F = 1.772 (3)	0.151	0.310
App	72.0% (16.9%)			
SMS	71.9% (17.1%)			
Website	56.9% (23.7%)			
**Sensors usage**		F = 4.046 (1) **	0.045	0.159
No sensors used	71.2% (17.0%)			
Sensors used	74.7% (16.5%)			
**EMA approach**		F = 4.019 (1) **	0.045	0.159
conventional EMA	71.8% (17.0%)			
micro-EMA (μEMA)	82.1% (10.5%)			

SD: standard deviation, DF: degrees of freedom, * p<0.1, ** p<0.05, *** p<0.01, $p_{adjusted}$: *p*-values corrected for multiple comparisons using the Benjamini–Hochberg method [25].

## Data Availability

The complete list of references used for this review is available in the Supplementary Materials.

## Supplementary Materials

The following supporting information can be downloaded at https://www.mdpi.com/article/10.3390/s24020472/s1.

## Abbreviations

The following abbreviations are used in this manuscript:

ANOVA	Analysis of Variance
EMA	Ecological Momentary Assessment
GPS	Global Positioning System
EC	Exclusion Criterion
IC	Inclusion Criterion
MCS	Mobile Crowdsensing
μEMA	Microinteraction-based Ecological Momentary Assessment
PRISMA	Preferred Reporting Items for Systematic Reviews and Meta-Analyses
SD	Standard Deviation
WHO	World Health Organization
