# Supplementary material for: Mobile Crowdsensing in Ecological Momentary Assessment mHealth Studies: A Systematic Review and Analysis
Source: Sensors (Basel). 2024 Jan 12;24(2):472. doi: 10.3390/s24020472 (PMC10820952; doi:10.3390/s24020472)
Supplement: Supplementary file 1 [file sensors-24-00472-s001.zip › sensors-2727552-supplementary.pdf]

Supplementary Materials: Mobile Crowdsensing in Ecological Momentary Assessment mHealth Studies: A Systematic Review and Analysis

Robin Kraft [1,2,3], Manfred Reichert [1] and Rüdiger Pryss [3,*]

Complete List of References

1. Pryss, R.; Schlee, W.; Reichert, M.; Kurthen, I.; Giroud, N.; Jagoda, L.; Neuschwander, P.; Meyer, M.; Neff, P.; Schobel, J.; et al. Ecological Momentary Assessment based Differences between Android and iOS Users of the TrackYourHearing mHealth Crowdsensing Platform. In Proceedings of the 2019 41st Annual International Conference of the IEEE Engineering in Medicine and Biology Society (EMBC), 2019, pp. 3951–3955. https://doi.org/10.1109/EMBC.2019.8857854.
2. Noël, C.; Armiento, C.; Péfoyo, A.K.; Klein, R.; Bédard, M.; Scharf, D. Adolescent exposure to cannabis marketing following recreational cannabis legalization in Canada: A pilot study using ecological momentary assessment. *Addictive behaviors reports* **2021**, *14*, 100383.
3. Bernstein, E.E.; Bentley, K.H.; Nock, M.K.; Stein, M.B.; Beck, S.; Kleiman, E.M. An Ecological Momentary Intervention Study of Emotional Responses to Smartphone-Prompted CBT Skills Practice and the Relationship to Clinical Outcomes. *Behavior therapy* **2022**, *53*, 267–280.
4. Schlee, W.; Simoes, J.; Pryss, R. Auricular Acupressure Combined with Self-Help Intervention for Treating Chronic Tinnitus: A Longitudinal Observational Study. *Journal of clinical medicine* **2021**, *10*.
5. Janssens, M.; Janssens, E.; Eshuis, J.; Lataster, J.; Simons, M.; Reijnders, J.; Jacobs, N. Companion Animals as Buffer against the Impact of Stress on Affect: An Experience Sampling Study. *Animals : an open access journal from MDPI* **2021**, *11*.
6. Stoffel, M.; Abbruzzese, E.; Rahn, S.; Bossmann, U.; Moessner, M.; Ditzen, B. Covariation of psychobiological stress regulation with valence and quantity of social interactions in everyday life: disentangling intra- and interindividual sources of variation. *Journal of neural transmission (Vienna, Austria : 1996)* **2021**, *128*, 1381–1395.
7. Schwaninger, P.; Berli, C.; Lüscher, J.; Scholz, U. Cultivation or enabling? Day-to-day associations between self-efficacy and received support in couples. *Social science & medicine (1982)* **2021**, *287*, 114330.
8. daSilva, A.W.; Huckins, J.F.; Wang, W.; Wang, R.; Campbell, A.T.; Meyer, M.L. Daily perceived stress predicts less next day social interaction: Evidence from a naturalistic mobile sensing study. *Emotion (Washington, D.C.)* **2021**, *21*, 1760–1770.
9. Hébert, E.T.; Bhushan, T.; Ra, C.K.; Frank-Pearce, S.; Alexander, A.C.; Cole, A.B.; Kendzor, D.E.; Businelle, M.S. Daily use of nicotine replacement medications is related to daily smoking status: An ecological momentary assessment study. *Drug and alcohol dependence* **2021**, *229*, 109161.
10. Feneberg, A.C.; Forbes, P.A.G.; Piperno, G.; Pronizius, E.; Stijovic, A.; Skoluda, N.; Lamm, C.; Nater, U.M.; Silani, G. Diurnal dynamics of stress and mood during COVID-19 lockdown: a large multinational ecological momentary assessment study. *Proceedings. Biological sciences* **2022**, *289*, 20212480.
11. Rabinowitz, A.; Hart, T.; Wilson, J. Ecological momentary assessment of affect in context after traumatic brain injury. *Rehabilitation psychology* **2021**, *66*, 442–449.
12. Grégoire, S.; Chénier, C.; Doucerain, M.; Lachance, L.; Shankland, R. Ecological Momentary Assessment of Stress, Well-Being, and Psychological Flexibility Among College and University Students During Acceptance and Commitment Therapy. *Canadian Journal of Behavioural Science* **2020**, *52*, 231–243.
13. Nguyen-Feng, V.N.; Romano, F.N.; Frazier, P. Emotional Abuse Moderates Efficacy of an Ecological Momentary Stress Management Intervention for College Students. *Journal of Counseling Psychology* **2019**, *66*, 461–472.
14. Bennett, B.L.; Whisenhunt, B.L.; Hudson, D.L.; Wagner, A.F.; Latner, J.D.; Stefano, E.C.; Beauchamp, M.T. Examining the Impact of Social Media on Mood and Body Dissatisfaction Using Ecological Momentary Assessment. *Journal of American College Health* **2020**, *68*, 502–508.
15. Hancock, K.E.; Downward, P.; Sherar, L.B. Exploring Feelings of Pleasure and Purpose Associated With Older People's Activities Using Ecological Momentary Analysis: An Observational Study. *Journal of aging and physical activity* **2021**, *29*, 670–677.
16. Pokhrel, P.; Phillips, K.T.; Kawamoto, C.T.; Taketa, R.; Tabangcura, K.J.; Yoshioka-Maxwell, A.; Pagano, I. Exposure to e-cigarette content on social media and e-cigarette use: An ecological momentary assessment study. *Addictive behaviors reports* **2021**, *14*, 100368.
17. Anderson Goodell, E.M.; Nordeck, C.; Finan, P.H.; Vandrey, R.; Dunn, K.E.; Thrul, J. Feasibility and acceptability of using smartphone-based EMA to assess patterns of prescription opioid and medical cannabis use among individuals with chronic pain. *Internet interventions* **2021**, *26*, 100460.
18. Goldstein, S.P.; Thomas, J.G.; Brick, L.A.; Zhang, F.; Forman, E.M. Identifying behavioral types of dietary lapse from a mobile weight loss program: Preliminary investigation from a secondary data analysis. *Appetite* **2021**, *166*, 105440.

19. Dabit, S.; Quraishi, S.; Jordan, J.; Biagianti, B. Improving social functioning in people with schizophrenia-spectrum disorders via mobile experimental interventions: Results from the CLIMB pilot trial. *Schizophrenia research. Cognition* **2021**, *26*, 100211.
20. O'Neill, E.R.; Basile, J.D.; Nelson, P. Individual Hearing Outcomes in Cochlear Implant Users Influence Social Engagement and Listening Behavior in Everyday Life. *Journal of speech, language, and hearing research : JSLHR* **2021**, *64*, 4982–4999.
21. Boronat, A.; Clivillé-Pérez, J.; Soldevila-Domenech, N.; Forcano, L.; Pizarro, N.; Fitó, M.; Schröder, H.; Fauria, K.; de la Torre, R. Mobile Device-assisted Dietary Ecological Momentary Assessments for the Evaluation of the Adherence to the Mediterranean Diet in a Continuous Manner. *Journal of visualized experiments : JoVE* **2021**.
22. Siddiquee, A.; Sixsmith, J.; Lawthom, R.; Haworth, J. Paid work, life-work and leisure: a study of wellbeing in the context of academic lives in higher education. *Leisure Studies* **2016**, *35*, 36–45.
23. Wu, Y.H.; Stangl, E.; Oleson, J.; Caraher, K.; Dunn, C.C. Personal Characteristics Associated with Ecological Momentary Assessment Compliance in Adult Cochlear Implant Candidates and Users. *Journal of the American Academy of Audiology* **2021**.
24. Porras-Segovia, A.; Díaz-Oliván, I.; Barrigón, M.L.; Moreno, M.; Artés-Rodríguez, A.; Pérez-Rodríguez, M.M.; Baca-García, E. Real-world feasibility and acceptability of real-time suicide risk monitoring via smartphones: A 6-month follow-up cohort. *Journal of psychiatric research* **2022**, *149*, 145–154.
25. Bomyea, J.A.; Parrish, E.M.; Paolillo, E.W.; Filip, T.F.; Eyler, L.T.; Depp, C.A.; Moore, R.C. Relationships between daily mood states and real-time cognitive performance in individuals with bipolar disorder and healthy comparators: A remote ambulatory assessment study. *Journal of clinical and experimental neuropsychology* **2021**, *43*, 813–824.
26. Morris, A.S.; Mackintosh, K.A.; Owen, N.; Dempsey, P.C.; Dunstan, D.W.; McNarry, M.A. Rise and Recharge: Exploring Employee Perceptions of and Contextual Factors Influencing an Individual-Level E-Health Smartphone Intervention to Reduce Office Workers' Sedentary Time at Work. *International journal of environmental research and public health* **2021**, *18*.
27. Draijer, J.; Bakker, A.; Slot, E.; Akkerman, S. The Multidimensional Structure of Interest. *Frontline Learning Research* **2020**, *8*, 18–36.
28. Giessing, L.; Oudejans, R.R.D.; Hutter, V.; Plessner, H.; Strahler, J.; Frenkel, M.O. Acute and Chronic Stress in Daily Police Service: A Three-Week N-of-1 Study. *Psychoneuroendocrinology* **2020**, *122*, 104865.
29. Duif, M.; Thewissen, V.; Wouters, S.; Lechner, L.; Jacobs, N. Affective Instability and Alcohol Consumption: Ecological Momentary Assessment in an Adult Sample. *Journal of studies on alcohol and drugs* **2019**, *80*, 441–447.
30. Câmara-Souza, M.B.; Carvalho, A.G.; Figueredo, O.M.C.; Bracci, A.; Manfredini, D.; Rodrigues Garcia, R.C.M. Awake bruxism frequency and psychosocial factors in college preparatory students. *Cranio : the journal of craniomandibular practice* **2020**, pp. 1–7.
31. Giurgiu, M.; Koch, E.D.; Plotnikoff, R.C.; Ebner-Priemer, U.W.; Reichert, M. Breaking Up Sedentary Behavior Optimally to Enhance Mood. *Medicine and science in sports and exercise* **2020**, *52*, 457–465.
32. Jones, S.E.; Moore, R.C.; Depp, C.A.; Ackerman, R.A.; Pinkham, A.E.; Harvey, P.D. Daily Ecological Momentary Assessments of happy and sad moods in people with schizophrenia and bipolar disorders: What do participants who are never sad think about their activities and abilities? *Schizophrenia research. Cognition* **2021**, *26*, 100202.
33. Liu, H.; Lou, V.W.Q. Developing a smartphone-based ecological momentary assessment protocol to collect biopsychosocial data with community-dwelling late-middle-aged and older adults. *Translational behavioral medicine* **2019**, *9*, 711–719.
34. Hjartarson, K.H.; Snorrason, I.; Bringmann, L.F.; Ögmundsson, B.E.; Ólafsson, R.P. Do daily mood fluctuations activate ruminative thoughts as a mental habit? Results from an ecological momentary assessment study. *Behaviour research and therapy* **2021**, *140*, 103832.
35. McIntyre, R.S.; Lee, Y.; Rong, C.; Rosenblat, J.D.; Brietzke, E.; Pan, Z.; Park, C.; Subramaniapillai, M.; Ragguett, R.M.; Mansur, R.B.; et al. Ecological momentary assessment of depressive symptoms using the mind.me application: Convergence with the Patient Health Questionnaire-9 (PHQ-9). *Journal of psychiatric research* **2021**, *135*, 311–317.
36. Hensler, I.; Sveen, J.; Cernvall, M.; Arnberg, F.K. Ecological momentary assessment of self-rated health, daily strategies and self-management app use among trauma-exposed adults. *European journal of psychotraumatology* **2021**, *12*, 1920204.
37. Hawker, C.O.; Merkouris, S.S.; Youssef, G.J.; Dowling, N.A. Exploring the associations between gambling cravings, self-efficacy, and gambling episodes: An Ecological Momentary Assessment study. *Addictive behaviors* **2021**, *112*, 106574.
38. Loeffler, S.N.; Stumpp, J.; Grund, S.; Limberger, M.F.; Ebner-Priemer, U.W. Fostering self-regulation to overcome academic procrastination using interactive ambulatory assessment. *Learning & Individual Differences* **2019**, *75*, N.PAG–N.PAG.
39. Nap-van der Vlist, M.M.; Houtveen, J.; Dalmeijer, G.W.; Grootenhuis, M.A.; van der Ent, C.K.; van Grotel, M.; Swart, J.F.; van Montfrans, J.M.; van de Putte, E.M.; Nijhof, S.L. Internet and smartphone-based ecological momentary assessment and personalized advice (PROfeel) in adolescents with chronic conditions: A feasibility study. *Internet interventions* **2021**, *25*, 100395.
40. Golan, D.; Sagiv, S.; Glass-Marmor, L.; Miller, A. Mobile-phone-based e-diary derived patient reported outcomes: Association with clinical disease activity, psychological status and quality of life of patients with multiple sclerosis. *PloS one* **2021**, *16*, e0250647.
41. Juarascio, A.S.; Crochiere, R.J.; Tapera, T.M.; Palermo, M.; Zhang, F. Momentary changes in heart rate variability can detect risk for emotional eating episodes. *Appetite* **2020**, *152*, 104698.
42. Fernie, B.A.; Spada, M.M.; Brown, R.G. Motor fluctuations and psychological distress in Parkinson's disease. *Health psychology : official journal of the Division of Health Psychology, American Psychological Association* **2019**, *38*, 518–526.
43. Lenaert, B.; Neijmeijer, M.; van Kampen, N.; van Heugten, C.; Ponds, R. Poststroke Fatigue and Daily Activity Patterns During Outpatient Rehabilitation: An Experience Sampling Method Study. *Archives of physical medicine and rehabilitation* **2020**, *101*, 1001–1008.

44. Le, T.P.; Moscardini, E.; Cowan, T.; Elvevåg, B.; Holmlund, T.B.; Foltz, P.W.; Tucker, R.P.; Schwartz, E.K.; Cohen, A.S. Predicting self-injurious thoughts in daily life using ambulatory assessment of state cognition. *Journal of psychiatric research* **2021**, *138*, 335–341.
45. Porras-Segovia, A.; Molina-Madueño, R.M.; Berrouiguet, S.; López-Castroman, J.; Barrigón, M.L.; Pérez-Rodríguez, M.S.; Marco, J.H.; Díaz-Oliván, I.; de León, S.; Courtet, P.; et al. Smartphone-based ecological momentary assessment (EMA) in psychiatric patients and student controls: A real-world feasibility study. *Journal of affective disorders* **2020**, *274*, 733–741.
46. Kenyon, C.C.; Sundar, K.G.; Gruschow, S.M.; Quarshie, W.O.; Feudtner, C.; Bryant-Stephens, T.C.; Miller, V.A. Tailored medication adherence incentives for high-risk children with asthma: a pilot study. *The Journal of asthma : official journal of the Association for the Care of Asthma* **2020**, *57*, 1372–1378.
47. Krug, I.; Selvaraja, P.; Fuller-Tyszkiewicz, M.; Hughes, E.K.; Slater, A.; Griffiths, S.; Yee, Z.W.; Richardson, B.; Blake, K. The effects of fitspiration images on body attributes, mood and eating behaviors: An experimental Ecological Momentary Assessment study in females. *Body image* **2020**, *35*, 279–287.
48. Schmid, R.F.; Thomas, J. The interactive effects of heart rate variability and mindfulness on indicators of well-being in healthcare professionals' daily working life. *International journal of psychophysiology : official journal of the International Organization of Psychophysiology* **2021**, *164*, 130–138.
49. Brys, A.D.H.; Stifft, F.; Van Heugten, C.M.; Bossola, M.; Gambaro, G.; Lenaert, B. Unraveling Fatigue in Hemodialysis Patients: Comparing Retrospective Reports to Real-Time Assessments With an mHealth Experienced Sampling Method. *Journal of pain and symptom management* **2020**, *60*, 1100.
50. Lin, W.C.; Burke, L.; Schlenk, E.A.; Yeh, C.H. Use of an Ecological Momentary Assessment Application to Assess the Effects of Auricular Point Acupressure for Chronic Low Back Pain. *Computers, informatics, nursing : CIN* **2019**, *37*, 276–282.
51. Courtney, J.B.; Lipsey, T.; Braun, B.; Henry, K.; Nelson, T.L.; Li, K. Using Ecological Momentary Assessment to Examine the Effects of Duty Status on Acute Stress and Tiredness in Firefighters: A Pilot Study. *Journal of occupational and environmental medicine* **2020**, *62*, 859–870.
52. Törnros, T.; Dorn, H.; Reichert, M.; Ebner-Priemer, U.; Salize, H.J.; Tost, H.; Meyer-Lindenberg, A.; Zipf, A. A comparison of temporal and location-based sampling strategies for global positioning system-triggered electronic diaries. *Geospatial health* **2016**, *11*, 473.
53. Greene, T. Do acute dissociation reactions predict subsequent posttraumatic stress and growth? A prospective experience sampling method study. *Journal of anxiety disorders* **2018**, *57*, 1–6.
54. Magallón-Neri, E.; Kirchner, T.; Forns, M.; Calderón, C.; Planellas, I. Ecological momentary assessment of contextual variables, satisfaction, and emotional and behavioral states of adolescents by level of victimization. *Child abuse & neglect* **2018**, *80*, 268–276.
55. Fatseas, M.; Serre, F.; Swendsen, J.; Auriacombe, M. Effects of anxiety and mood disorders on craving and substance use among patients with substance use disorder: An ecological momentary assessment study. *Drug and alcohol dependence* **2018**, *187*, 242–248.
56. Cheong, I.Y.; An, S.Y.; Cha, W.C.; Rha, M.Y.; Kim, S.T.; Chang, D.K.; Hwang, J.H. Efficacy of Mobile Health Care Application and Wearable Device in Improvement of Physical Performance in Colorectal Cancer Patients Undergoing Chemotherapy. *Clinical colorectal cancer* **2018**, *17*, e353–e362.
57. Bakker, D.; Rickard, N. Engagement in mobile phone app for self-monitoring of emotional wellbeing predicts changes in mental health: MoodPrism. *Journal of affective disorders* **2018**, *227*, 432–442.
58. Boesen, V.B.; Feldt-Rasmussen, U.; Bjorner, J.B.; Cramon, P.; Groenvold, M.; Nygaard, B.; Rasmussen, K.; Vilsbøll, T.; Watt, T. How Should Thyroid-Related Quality of Life Be Assessed? Recalled Patient-Reported Outcomes Compared to Here-and-Now Measures. *Thyroid : official journal of the American Thyroid Association* **2018**, *28*, 1561–1570.
59. Geyer, E.C.; Fua, K.C.; Daniel, K.E.; Chow, P.I.; Bonelli, W.; Huang, Y.; Barnes, L.E.; Teachman, B.A. I Did OK, but Did I Like It? Using Ecological Momentary Assessment to Examine Perceptions of Social Interactions Associated With Severity of Social Anxiety and Depression. *Behavior therapy* **2018**, *49*, 866–880.
60. Reichenberger, J.; Richard, A.; Smyth, J.M.; Fischer, D.; Pollatos, O.; Blechert, J. It's craving time: time of day effects on momentary hunger and food craving in daily life. *Nutrition (Burbank, Los Angeles County, Calif.)* **2018**, *55*, 15–20.
61. Hallensleben, N.; Glaesmer, H.; Forkmann, T.; Rath, D.; Strauss, M.; Kersting, A.; Spangenberg, L. Predicting suicidal ideation by interpersonal variables, hopelessness and depression in real-time. An ecological momentary assessment study in psychiatric inpatients with depression. *European psychiatry : the journal of the Association of European Psychiatrists* **2019**, *56*, 43–50.
62. Ciere, Y.; Snippe, E.; Padberg, M.; Jacobs, B.; Visser, A.; Sanderman, R.; Fleer, J. The role of state and trait positive affect and mindfulness in affective reactivity to pain in chronic migraine. *Health psychology : official journal of the Division of Health Psychology, American Psychological Association* **2019**, *38*, 94–102.
63. Bedard, C.; King-Dowling, S.; McDonald, M.; Dunton, G.; Cairney, J.; Kwan, M. Understanding Environmental and Contextual Influences of Physical Activity During First-Year University: The Feasibility of Using Ecological Momentary Assessment in the MovingU Study. *JMIR public health and surveillance* **2017**, *3*, e32.
64. Minor, K.S.; Davis, B.J.; Marggraf, M.P.; Luther, L.; Robbins, M.L. Words matter: Implementing the electronically activated recorder in schizotypy. *Personality disorders* **2018**, *9*, 133–143.
65. Weatherson, K.; Yun, L.; Wunderlich, K.; Puterman, E.; Faulkner, G. Application of an Ecological Momentary Assessment Protocol in a Workplace Intervention: Assessing Compliance, Criterion Validity, and Reactivity. *Journal of physical activity & health* **2019**, *16*, 985–992.

66. O'Donnell, R.; Richardson, B.; Fuller-Tyszkiewicz, M.; Liknaitzky, P.; Arulkadacham, L.; Dvorak, R.; Staiger, P.K. Ecological momentary assessment of drinking in young adults: An investigation into social context, affect and motives. *Addictive behaviors* **2019**, *98*, 106019.
67. Remmerswaal, D.; Jongerling, J.; Jansen, P.J.; Eielts, C.; Franken, I.H.A. Impaired subjective self-control in alcohol use: An ecological momentary assessment study. *Drug and alcohol dependence* **2019**, *204*, 107479.
68. Drott, J.; Fomichov, V.; Starkhammar, H.; Börjeson, S.; Kjellgren, K.; Berterö, C. Oxaliplatin-Induced Neurotoxic Side Effects and Their Impact on Daily Activities: A Longitudinal Study Among Patients With Colorectal Cancer. *Cancer nursing* **2019**, *42*, E40–E48.
69. Beddig, T.; Reinhard, I.; Kuehner, C. Stress, mood, and cortisol during daily life in women with Premenstrual Dysphoric Disorder (PMDD). *Psychoneuroendocrinology* **2019**, *109*, 104372.
70. Parrish EM.; Kamarsu S.; Harvey PD.; Pinkham A.; Depp CA.; Moore RC. Remote Ecological Momentary Testing of Learning and Memory in Adults With Serious Mental Illness. *Schizophrenia bulletin* **2021**, *47*, 740–750.
71. Gansner M.; Nisenson M.; Carson N.; Torous J. A pilot study using ecological momentary assessment via smartphone application to identify adolescent problematic internet use. *Psychiatry research* **2020**, *293*, 113428.
72. Yee ZW.; Griffiths S.; Fuller-Tyszkiewicz M.; Blake K.; Richardson B.; Krug I. The differential impact of viewing fitspiration and thinspiration images on men's body image concerns: An experimental ecological momentary assessment study. *Body image* **2020**, *35*, 96–107.
73. von Stumm S.; Latham RM. Early life experiences: Meaningful differences within and between families. *Infant behavior & development* **2018**, *53*, 56–63.
74. Hennig T.; Krkovic K.; Lincoln TM. What predicts inattention in adolescents? An experience-sampling study comparing chronotype, subjective, and objective sleep parameters. *Sleep medicine* **2017**, *38*, 58–63.
75. Howe LK.; Copeland S.; Fisher L.; Farmer E.; Nemes L.; Finn PR. Mobile assessment of decisions to drink in young adults: Examining the role of incentives and disincentives. *Alcoholism, clinical and experimental research* **2022**, *46*, 152–165.
76. Burnell K.; George MJ.; Jensen M.; Hoyle RH.; Odgers CL. Associations Between Adolescents' Daily Digital Technology Use and Sleep. *The Journal of adolescent health : official publication of the Society for Adolescent Medicine* **2022**, *70*, 450–456.
77. Groefsema M.; Luijten M.; Engels R.; Kuntsche E. Young adults do not catch up missed drinks when starting later at night-An ecological momentary assessment study. *Experimental and clinical psychopharmacology* **2019**, *27*, 160–165.
78. Keating L.; Mills JS.; Rawana JS. Momentary predictors of binge eating: An attachment perspective. *Eating behaviors* **2019**, *32*, 44–52.
79. Fuller-Tyszkiewicz M.; Vuong H.; Linardon J.; Krug I.; Broadbent J.; Rodgers RF. Body image in and out of the lab: Correspondence between lab-based attentional bias data and body shape dissatisfaction experiences in daily life. *Body image* **2020**, *32*, 62–69.
80. Konig, L.; Renner, B. Colourful = healthy? Exploring meal colour variety and its relation to food consumption. *FOOD QUALITY AND PREFERENCE* **2018**, *64*, 66–71.
81. Erickson, B.; Herman, T.; Hahn, A.; Taple, B.; Bass, M.; Lloyd, R.; Sutcliffe, S.; Griffith, J.; Multidisciplinary Approach Study C. A Mobile Phone Application for Assessing Daily Variation in Pain Location and Pain Intensity in Patients with Urologic Chronic Pelvic Pain Syndrome: A MAPP Network Study. *UROLOGY PRACTICE* **2021**, *8*, 189–195.
82. van Berkel, N.; Goncalves, J.; Loven, L.; Ferreira, D.; Hosio, S.; Kostakos, V. Effect of experience sampling schedules on response rate and recall accuracy of objective self-reports. *INTERNATIONAL JOURNAL OF HUMAN-COMPUTER STUDIES* **2019**, *125*, 118–128.
83. Burke, T.; Shao, S.; Jacobucci, R.; Kautz, M.; Alloy, L.; Ammerman, B. Examining momentary associations between behavioral approach system indices and nonsuicidal self-injury urges. *JOURNAL OF AFFECTIVE DISORDERS* **2022**, *296*, 244–249.
84. Targum, S.; Sauder, C.; Evans, M.; Saber, J.; Harvey, P. Ecological momentary assessment as a measurement tool in depression trials. *JOURNAL OF PSYCHIATRIC RESEARCH* **2021**, *136*, 256–264.
85. Zhang, Z.; Xiao, H.; Gou, X.; Li, M.; Zheng, J. "I didn't want to do, but I am happy to now!" Affect, voice and in-role performance. *KYBERNETES* **2021**, *50*, 1467–1482.
86. Zhang, Z.; Zhang, L.; Xiao, H.; Zheng, J. Information quality, media richness, and negative coping: A daily research during the COVID-19 pandemic. *PERSONALITY AND INDIVIDUAL DIFFERENCES* **2021**, *176*.
87. Koudela-Hamila, S.; Santangelo, P.; Ebner-Priemer, U.; Schlotz, W. Under Which Circumstances Does Academic Workload Lead to Stress? Explaining Intraindividual Differences by Using the Cortisol-Awakening Response as a Moderator. *JOURNAL OF PSYCHOPHYSIOLOGY* **2022**, *36*, 188–197.
88. Weijs-Perree, M.; Dane, G.; van den Berg, P. Analyzing the Relationships between Citizens' Emotions and their Momentary Satisfaction in Urban Public Spaces. *SUSTAINABILITY* **2020**, *12*.
89. Goldstein, S.P.; Evans, E.W.; Espel-Huynh, H.M.; Goldstein, C.M.; Karchere-Sun, R.; Thomas, J.G. Dietary lapses are associated with meaningful elevations in daily caloric intake and added sugar consumption during a lifestyle modification intervention. *Obesity Science & Practice* **2022**, *8*, 442–454. https://doi.org/10.1002/osp4.587.
90. Gregersen, T.; Mercer, S.; MacIntyre, P.; Talbot, K.; Banga, C.A. Understanding language teacher wellbeing: An ESM study of daily stressors and uplifts. *Language Teaching Research* **2020**, p. 1362168820965897. https://doi.org/10.1177/1362168820965897.
91. Cook, J.; Kabubo-Mariara, J.; Kimuyu, P. Happy at Work in Africa? Measuring Hedonic Well-Being Among Water Carriers in Rural Kenya Using the Experience Sampling Method. *Journal of Happiness Studies* **2022**, *23*, 2449–2468. https://doi.org/10.1007/s10902-022-00509-2.

92. Zarbo, C.; Agosta, S.; Casiraghi, L.; Novellis, A.; Leuci, E.; Paulillo, G.; Rocchetti, M.; Starace, F.; Zamparini, M.; de Girolamo, G. Assessing adherence to and usability of Experience Sampling Method (ESM) and actigraph in patients with Schizophrenia Spectrum Disorder: A mixed-method study. *Psychiatry research* **2022**, *314*, 114675. https://doi.org/10.1016/j.psychres.2022.114675.
93. van Munster, K.N.; Dijkgraaf, M.G.W.; Oude Elferink, R.P.J.; Beuers, U.; Ponsioen, C.Y. Symptom patterns in the daily life of PSC patients. *Liver International* **2022**, *42*, 1562–1570. https://doi.org/10.1111/liv.15271.
94. O'Rourke, T.; Vogel, C.; John, D.; Pryss, R.; Schobel, J.; Haug, F.; Haug, J.; Pieh, C.; Nater, U.M.; Feneberg, A.C.; et al. The Impact of Coping Styles and Gender on Situational Coping: An Ecological Momentary Assessment Study With the mHealth Application TrackYourStress. *Frontiers in Psychology* **2022**, *13*, 913125. https://doi.org/10.3389/fpsyg.2022.913125.
95. Sandberg, J.; Sundh, J.; Anderberg, P.; Currow, D.C.; Johnson, M.; Lansing, R.; Ekström, M. Comparing recalled versus experienced symptoms of breathlessness ratings: An ecological assessment study using mobile phone technology. *Respirology*, *n/a*. https://doi.org/10.1111/resp.14313.
96. Silva, C.; Cero, I.; Ricci, N.; Pérez, A.; Conwell, Y.; Van Orden, K. The feasibility and acceptability of using smartphones to assess suicide risk among Spanish-speaking adult outpatients. *Suicide and Life-Threatening Behavior*, *n/a*. https://doi.org/10.1111/sltb.12889.
97. Fortuna, K.L.; Wright, A.C.; Mois, G.; Myers, A.L.; Kadakia, A.; Collins-Pisano, C. Feasibility, Acceptability, and Potential Utility of Peer-supported Ecological Momentary Assessment Among People with Serious Mental Illness: a Pilot Study. *The Psychiatric Quarterly* **2022**, pp. 1–19. https://doi.org/10.1007/s11126-022-09986-3.
98. Dramburg, S.; Perna, S.; Di Fraia, M.; Tripodi, S.; Arasi, S.; Castelli, S.; Villalta, D.; Buzzulini, F.; Sfika, I.; Villella, V.; et al. Validation Parameters of Patient-Generated Data for Digitally Recorded Allergic Rhinitis Symptom and Medication Scores in the @IT.2020 Project: Exploratory Study (Preprint). preprint, JMIR mHealth and uHealth, 2021.
99. Hisler, G.C.; Pedersen, S.L.; Hasler, B.P. The 24-hour rhythm in alcohol craving and individual differences in sleep characteristics and alcohol use frequency. *Alcoholism: Clinical and Experimental Research* **2022**, *46*, 1084–1093. https://doi.org/10.1111/acer.14826.
100. Fletcher, K.; Lindblom, K.; Seabrook, E.; Foley, F.; Murray, G. Pilot Testing in the Wild: Feasibility, Acceptability, Usage Patterns, and Efficacy of an Integrated Web and Smartphone Platform for Bipolar II Disorder. *JMIR Formative Research* **2022**, *6*, e32740. https://doi.org/10.2196/32740.
101. Forster, S.D.; Gauggel, S.; Loevenich, R.; Völzke, V.; Petershofer, A.; Zimmermann, P.; Privou, C.; Bonnert, J.; Mainz, V. A Microanalysis of Mood and Self-Reported Functionality in Stroke Patients Using Ecological Momentary Assessment. *Frontiers in Neurology* **2022**, *13*, 854777. https://doi.org/10.3389/fneur.2022.854777.
102. Maes, I.; Mertens, L.; Poppe, L.; Crombez, G.; Vetrovsky, T.; Van Dyck, D. The variability of emotions, physical complaints, intention, and self-efficacy: an ecological momentary assessment study in older adults. *PeerJ* **2022**, *10*, e13234. https://doi.org/10.7717/peerj.13234.
103. Alvarez, E.E.; Hafezi, S.; Bonagura, D.; Kleiman, E.M.; Konova, A.B. A Proof-of-Concept Ecological Momentary Assessment Study of Day-Level Dynamics in Value-Based Decision-Making in Opioid Addiction. *Frontiers in Psychiatry* **2022**, *13*, 817979. https://doi.org/10.3389/fpsyt.2022.817979.
104. Bui, Q.; Kaufman, K.J.; Munsell, E.G.; Lenze, E.J.; Lee, J.M.; Mohr, D.C.; Fong, M.W.; Metts, C.L.; Tomazin, S.E.; Pham, V.; et al. Smartphone assessment uncovers real-time relationships between depressed mood and daily functional behaviors after stroke. *Journal of Telemedicine and Telecare* **2022**, p. 1357633X221100061. https://doi.org/10.1177/1357633X221100061.
105. Nereim, C.; Bickham, D.; Rich, M. Exploring Use Patterns and Racial and Ethnic Differences in Real Time Affective States During Social Media Use Among a Clinical Sample of Adolescents With Depression: Prospective Cohort Study. *JMIR Formative Research* **2022**, *6*, e30900. https://doi.org/10.2196/30900.
106. Scherer, E.A.; Kim, S.J.; Metcalf, S.A.; Sweeney, M.A.; Wu, J.; Xie, H.; Mazza, G.L.; Valente, M.J.; MacKinnon, D.P.; Marsch, L.A. Momentary Self-regulation: Scale Development and Preliminary Validation. *JMIR Mental Health* **2022**, *9*, e35273. https://doi.org/10.2196/35273.
107. Suga, A.; Naruto, Y.; Maulina, V.V.R.; Uraguchi, M.; Ozaki, Y.; Ohira, H. Mothers' interoceptive sensibility mediates affective interaction between mother and infant. *Scientific Reports* **2022**, *12*, 6273. https://doi.org/10.1038/s41598-022-09988-y.
108. Heathcote, L.C.; Cunningham, S.J.; Webster, S.N.; Tanna, V.; Mattke, E.; Loecher, N.; Spunt, S.L.; Simon, P.; Dahl, G.; Walentynowicz, M.; et al. Smartphone-based Ecological Momentary Assessment to study "scanxiety" among Adolescent and Young Adult survivors of childhood cancer: A feasibility study. *Psycho-Oncology* **2022**, *31*, 1322–1330. https://doi.org/10.1002/pon.5935.
109. Bos, F.M.; Schreuder, M.J.; George, S.V.; Doornbos, B.; Bruggeman, R.; van der Krieke, L.; Haarman, B.C.M.; Wichers, M.; Snippe, E. Anticipating manic and depressive transitions in patients with bipolar disorder using early warning signals. *International Journal of Bipolar Disorders* **2022**, *10*, 12. https://doi.org/10.1186/s40345-022-00258-4.
110. Crochiere, R.J.; Abber, S.R.; Taylor, L.C.; Sala, M.; Schumacher, L.M.; Goldstein, S.P.; Forman, E.M. Momentary predictors of dietary lapse from a mobile health weight loss intervention. *Journal of Behavioral Medicine* **2022**, *45*, 324–330. https://doi.org/10.1007/s10865-021-00264-4.
111. Compernolle, E.L.; Finch, L.E.; Hawkley, L.C.; Cagney, K.A. Home alone together: Differential links between momentary contexts and real-time loneliness among older adults from Chicago during versus before the COVID-19 pandemic. *Social Science & Medicine (1982)* **2022**, *299*, 114881. https://doi.org/10.1016/j.socscimed.2022.114881.

112. Dillon, K.H.; Glenn, J.J.; Dennis, P.A.; Mann, A.J.; Deming, C.A.; Aho, N.; Hertzberg, J.S.; DeBeer, B.B.; Meyer, E.C.; Morissette, S.B.; et al. Affective states and nonsuicidal self-injury (NSSI): Results from an ecological momentary assessment study of veterans with NSSI disorder. *Suicide and Life-Threatening Behavior* **2022**, *52*, 256–267. https://doi.org/10.1111/sltb.12818.
113. Layrón Folgado, J.E.; Conchado Peiró, A.; Marco, J.H.; Barrigón, M.L.; Baca-García, E.; Pérez Rodríguez, S. Trajectory Analysis of Suicidal Ideation in Spanish College Students Using Ecological Momentary Assessment. *Frontiers in Psychiatry* **2022**, *13*, 853464. https://doi.org/10.3389/fpsyt.2022.853464.
114. Lehmann, J.; Schreyer, I.; Riedl, D.; Tschuggnall, M.; Giesinger, J.M.; Ninkovic, M.; Huth, M.; Kronberger, I.; Rumpold, G.; Holzner, B. Usability evaluation of the Computer-Based Health Evaluation System (CHES) eDiary for patients with faecal incontinence: a pilot study. *BMC Medical Informatics and Decision Making* **2022**, *22*, 81. https://doi.org/10.1186/s12911-022-01818-5.
115. Wegner, L.; Haucke, M.N.; Heinzel, S.; Liu, S. Smartphone-Based Ecological Momentary Assessment of Coping with Loneliness amid COVID-19 in Germany. *International Journal of Environmental Research and Public Health* **2022**, *19*, 3946. https://doi.org/10.3390/ijerph19073946.
116. Jimenez Rincon, S.; Dou, N.; Murray-Kolb, L.E.; Hudy, K.; Mitchell, D.C.; Li, R.; Na, M. Daily food insecurity is associated with diet quality, but not energy intake, in winter and during COVID-19, among low-income adults. *Nutrition Journal* **2022**, *21*, 19. https://doi.org/10.1186/s12937-022-00768-y.
117. Lee, J.M.; Woon, R.; Ramsum, M.; Halperin, D.S.; Jain, R. User Engagement and Assessment of Treatment Effectiveness in Patients Using a Novel Digital mHealth App During Spinal Cord Stimulation Screening Trials. *JMIR Human Factors* **2022**, *9*, e35134. https://doi.org/10.2196/35134.
118. Militello, L.; Sobolev, M.; Okeke, F.; Adler, D.A.; Nahum-Shani, I. Digital Prompts to Increase Engagement With the Headspace App and for Stress Regulation Among Parents: Feasibility Study. *JMIR Formative Research* **2022**, *6*, e30606. https://doi.org/10.2196/30606.
119. Scherer, E.A.; Metcalf, S.A.; Whicker, C.L.; Bartels, S.M.; Grabinski, M.; Kim, S.J.; Sweeney, M.A.; Lemley, S.M.; Lavoie, H.; Xie, H.; et al. Momentary Influences on Self-Regulation in Two Populations With Health Risk Behaviors: Adults Who Smoke and Adults Who Are Overweight and Have Binge-Eating Disorder. *Frontiers in Digital Health* **2022**, *4*, 798895. https://doi.org/10.3389/fdgth.2022.798895.
120. Elavsky, S.; Blahošová, J.; Lebedíková, M.; Tkaczyk, M.; Tancos, M.; Plhák, J.; Sotolář, O.; Smahel, D. Researching the Links Between Smartphone Behavior and Adolescent Well-being With the FUTURE-WP4 (Modeling the Future: Understanding the Impact of Technology on Adolescent's Well-being Work Package 4) Project: Protocol for an Ecological Momentary Assessment Study. *JMIR Research Protocols* **2022**, *11*, e35984. https://doi.org/10.2196/35984.
121. Burke, L.; Naylor, G. Smartphone App–Based Noncontact Ecological Momentary Assessment With Experienced and Naïve Older Participants: Feasibility Study. *JMIR Formative Research* **2022**, *6*, e27677. https://doi.org/10.2196/27677.
122. van den Berg, L.; Brouwer, P.; Panda, N.; Hoogbergen, M.M.; Solsky, I.; Onnela, J.P.; Haynes, A.B.; Sidey-Gibbons, C.J. Feasibility and performance of smartphone-based daily micro-surveys among patients recovering from cancer surgery. *Quality of Life Research* **2022**, *31*, 579–587. https://doi.org/10.1007/s11136-021-02934-x.
123. Lam, K.; van Oirschot, P.; den Teuling, B.; Hulst, H.; de Jong, B.; Uitdehaag, B.; de Groot, V.; Killestein, J. Reliability, construct and concurrent validity of a smartphone-based cognition test in multiple sclerosis. *Multiple Sclerosis (Houndmills, Basingstoke, England)* **2022**, *28*, 300–308. https://doi.org/10.1177/13524585211018103.
124. Brüdern, J.; Hallensleben, N.; Höller, I.; Spangenberg, L.; Forkmann, T.; Rath, D.; Strauß, M.; Kersting, A.; Glaesmer, H. Sleep disturbances predict active suicidal ideation the next day: an ecological momentary assessment study. *BMC Psychiatry* **2022**, *22*, 65. https://doi.org/10.1186/s12888-022-03716-6.
125. Semborski, S.; Madden, D.R.; Dzubur, E.; Redline, B.; Rhoades, H.; Henwood, B.F. The Effect of Momentary Affect on Substance Use among Young Adults Who Experience Homelessness. *Substance use & misuse* **2022**, *57*, 329–336. https://doi.org/10.1080/10826084.2021.1995756.
126. Peters, E.M.; Dong, L.Y.; Thomas, T.; Khalaj, S.; Balbuena, L.; Baetz, M.; Osgood, N.; Bowen, R. Instability of Suicidal Ideation in Patients Hospitalized for Depression: An Exploratory Study Using Smartphone Ecological Momentary Assessment. *Archives of Suicide Research* **2022**, *26*, 56–69. https://doi.org/10.1080/13811118.2020.1783410.
127. Shin, K.E.; Newman, M.G.; Jacobson, N.C. Emotion network density is a potential clinical marker for anxiety and depression: Comparison of ecological momentary assessment and daily diary. *British Journal of Clinical Psychology* **2022**, *61*, 31–50. https://doi.org/10.1111/bjc.12295.
128. Arbel, R.; Mason, T.B.; Dunton, G.F. Transactional links between children daily emotions and internalizing symptoms: a six-wave ecological momentary assessment study. *Journal of Child Psychology and Psychiatry* **2022**, *63*, 68–77. https://doi.org/10.1111/jcpp.13432.
129. Beuchat, H.; Grandjean, L.; Despland, J.N.; Pascual-Leone, A.; Gholam, M.; Swendsen, J.; Kramer, U. Ecological momentary assessment of emotional processing: An exploratory analysis comparing daily life and a psychotherapy analogue session. *Counselling and Psychotherapy Research* **2022**, *22*, 345–356. https://doi.org/10.1002/capr.12455.
130. van Genugten, C.R.; Schuurmans, J.; Hoogendoorn, A.W.; Araya, R.; Andersson, G.; Baños, R.M.; Berger, T.; Botella, C.; Cerga Pashoja, A.; Cieslak, R.; et al. A Data-Driven Clustering Method for Discovering Profiles in the Dynamics of Major Depressive Disorder Using a Smartphone-Based Ecological Momentary Assessment of Mood. *Frontiers in Psychiatry* **2022**, *13*, 755809. https://doi.org/10.3389/fpsyt.2022.755809.

131. Fortea, L.; Tortella-Feliu, M.; Juaneda-Seguí, A.; De la Peña-Arteaga, V.; Chavarría-Elizondo, P.; Prat-Torres, L.; Soriano-Mas, C.; Lane, S.P.; Radua, J.; Fullana, M.A. Development and Validation of a Smartphone-Based App for the Longitudinal Assessment of Anxiety in Daily Life. *Assessment* **2021**, p. 10731911211065166. https://doi.org/10.1177/10731911211065166.
132. Kennedy-Malone, L.; Hevel, D.J.; Sappenfield, K.B.; Scheer, H.; Zecca, C.; Maher, J.P. Low-Income, Older African Americans' Engagement in and Perceptions of a Smartphone-Based Ecological Momentary Assessment Study of Physical Activity and Sedentary Behavior. *Innovation in Aging* **2021**, *6*, igab056. https://doi.org/10.1093/geroni/igab056.
133. Hammoud, R.; Tognin, S.; Bakolis, I.; Ivanova, D.; Fitzpatrick, N.; Burgess, L.; Smythe, M.; Gibbons, J.; Davidson, N.; Mechelli, A. Lonely in a crowd: investigating the association between overcrowding and loneliness using smartphone technologies. *Scientific Reports* **2021**, *11*, 24134. https://doi.org/10.1038/s41598-021-03398-2.
134. Czyz, E.K.; Koo, H.J.; Al-Dajani, N.; King, C.A.; Nahum-Shani, I. Predicting short-term suicidal thoughts in adolescents using machine learning: developing decision tools to identify daily level risk after hospitalization. *Psychological Medicine* **2021**, pp. 1–10. https://doi.org/10.1017/S0033291721005006.
135. van Genugten, C.R.; Schuurmans, J.; Hoogendoorn, A.W.; Araya, R.; Andersson, G.; Baños, R.; Botella, C.; Cerga Pashoja, A.; Cieslak, R.; Ebert, D.D.; et al. Examining the Theoretical Framework of Behavioral Activation for Major Depressive Disorder: Smartphone-Based Ecological Momentary Assessment Study. *JMIR Mental Health* **2021**, *8*, e32007. https://doi.org/10.2196/32007.
136. Muñoz-Ramírez, S.; Escribano-López, B.; Rodrigo-Casares, V.; Vergara-Hernández, C.; Gil-Mary, D.; Sorribes-Monrabal, I.; Garcés-Sánchez, M.; Muñoz-Del-Barrio, M.J.; Albors-Fernández, A.M.; Úbeda Sansano, M.I.; et al. Feasibility of a hybrid clinical trial for respiratory virus detection in toddlers during the influenza season. *BMC Medical Research Methodology* **2021**, *21*, 273. https://doi.org/10.1186/s12874-021-01474-9.
137. van Barneveld, E.; Lim, A.; van Hanegem, N.; Vork, L.; Herrewegh, A.; van Poll, M.; Manders, J.; van Osch, F.; Spaans, W.; van Koeveringe, G.; et al. Patient-Reported Outcome Measure for Real-time Symptom Assessment in Women With Endometriosis: Focus Group Study. *JMIR Formative Research* **2021**, *5*, e28782. https://doi.org/10.2196/28782.
138. Cerino, E.S.; Katz, M.J.; Wang, C.; Qin, J.; Gao, Q.; Hyun, J.; Hakun, J.G.; Roque, N.A.; Derby, C.A.; Lipton, R.B.; et al. Variability in Cognitive Performance on Mobile Devices Is Sensitive to Mild Cognitive Impairment: Results From the Einstein Aging Study. *Frontiers in Digital Health* **2021**, *3*, 758031. https://doi.org/10.3389/fdgth.2021.758031.
139. Trofholz, A.; Tate, A.; Janowiec, M.; Fertig, A.; Loth, K.; de Brito, J.N.; Berge, J. Ecological Momentary Assessment of Weight-Related Behaviors in the Home Environment of Children From Low-Income and Racially and Ethnically Diverse Households: Development and Usability Study. *JMIR Research Protocols* **2021**, *10*, e30525. https://doi.org/10.2196/30525.
140. Dias, R.; Vaz, R.; Rodrigues, M.J.; Serra-Negra, J.M.; Bracci, A.; Manfredini, D. Utility of Smartphone-based real-time report (Ecological Momentary Assessment) in the assessment and monitoring of awake bruxism: A multiple-week interval study in a Portuguese population of university students. *Journal of Oral Rehabilitation* **2021**, *48*, 1307–1313. https://doi.org/10.1111/joor.13259.
141. Czyz, E.K.; Yap, J.R.; King, C.A.; Nahum-Shani, I. Using Intensive Longitudinal Data to Identify Early Predictors of Suicide-Related Outcomes in High-Risk Adolescents: Practical and Conceptual Considerations. *Assessment* **2021**, *28*, 1949–1959. https://doi.org/10.1177/1073191120939168.
142. Hannah Lee, J. Perceptions towards an interaction partner predict social anxiety: an ecological momentary assessment study. *Cognition and Emotion* **2021**, *35*, 1479–1498. https://doi.org/10.1080/02699931.2021.1969339.
143. Goodridge, D.; Reis, N.; Neiser, J.; Haubrich, T.; Westberg, B.; Erickson-Lumb, L.; Storozinski, J.; Gonzales, C.; Michael, J.; Cammer, A.; et al. An App-Based Mindfulness-Based Self-compassion Program to Support Caregivers of People With Dementia: Participatory Feasibility Study. *JMIR Aging* **2021**, *4*, e28652. https://doi.org/10.2196/28652.
144. Rabbani, M.; Haque, M.M.; Das Dipal, D.; Zarif, M.I.I.; Iqbal, A.; Schwichtenberg, A.; Bansal, N.; Soron, T.R.; Ahmed, S.I.; Ahamed, S.I. An mCARE study on patterns of risk and resilience for children with ASD in Bangladesh. *Scientific Reports* **2021**, *11*, 21342. https://doi.org/10.1038/s41598-021-00793-7.
145. Schulz, P.J.; Andersson, E.M.; Bizzotto, N.; Norberg, M. Using Ecological Momentary Assessment to Study the Development of COVID-19 Worries in Sweden: Longitudinal Study. *Journal of Medical Internet Research* **2021**, *23*, e26743. https://doi.org/10.2196/26743.
146. Arigo, D.; Mogle, J.A.; Brown, M.M.; Gupta, A. A multi-study approach to refining ecological momentary assessment measures for use among midlife women with elevated risk for cardiovascular disease. *mHealth* **2021**, *7*, 53. https://doi.org/10.21037/mhealth-20-143.
147. Adams, L.; Igbinedion, G.; DeVinney, A.; Azasu, E.; Nestadt, P.; Thrul, J.; Joe, S. Assessing the Real-time Influence of Racism-Related Stress and Suicidality Among Black Men: Protocol for an Ecological Momentary Assessment Study. *JMIR Research Protocols* **2021**, *10*, e31241. https://doi.org/10.2196/31241.
148. Vîslă, A.; Zinbarg, R.; Hilpert, P.; Allemand, M.; Flückiger, C. Worry and Positive Episodes in the Daily Lives of Individuals With Generalized Anxiety Disorder: An Ecological Momentary Assessment Study. *Frontiers in Psychology* **2021**, *12*, 722881. https://doi.org/10.3389/fpsyg.2021.722881.
149. Maisto, S.A.; Simons, J.S.; Palfai, T.P.; Moskal, D.; Luehring-Jones, P. Daily Associations Among Alcohol Intoxication, Partner Familiarity, Participant Effortful Control, Urgency, and PrEP Uptake on Sexual Behavior in Men Who Have Sex with Men. *Archives of Sexual Behavior* **2021**, *50*, 2843–2860. https://doi.org/10.1007/s10508-020-01852-2.

150. Wessel, J.; Bradley, G.L.; Hood, M. A Low-Intensity, High-Frequency Intervention to Reduce Procrastination. *Applied Psychology* **2021**, *70*, 1669–1690. https://doi.org/10.1111/apps.12293.
151. Lim, V.H.T.; Chen, Y.W.R.; Tseng, M.H.; Bundy, A.; Cordier, R. The impact of caregiver stigma on real-life social experience of Taiwanese adolescents with autism spectrum disorder. *Autism* **2021**, *25*, 1859–1871. https://doi.org/10.1177/13623613211004329.
152. Webb, C.A.; Swords, C.M.; Murray, L.; Hilt, L.M. App-Based Mindfulness Training for Adolescent Rumination: Predictors of Immediate and Cumulative Benefit. *Mindfulness* **2021**, *12*, 2498–2509. https://doi.org/10.1007/s12671-021-01719-0.
153. Lin, W.; Farella, M.; Antoun, J.S.; Topless, R.K.; Merriman, T.R.; Michelotti, A. Factors associated with orthodontic pain. *Journal of Oral Rehabilitation* **2021**, *48*, 1135–1143. https://doi.org/10.1111/joor.13227.
154. Markowski, K.L.; Smith, J.A.; Gauthier, G.R.; Harcey, S.R. Patterns of Missing Data With Ecological Momentary Assessment Among People Who Use Drugs: Feasibility Study Using Pilot Study Data. *JMIR Formative Research* **2021**, *5*, e31421. https://doi.org/10.2196/31421.
155. Holliday, A.; Johnson, K.O.; Kaiseler, M.; Crabtree, D.R. APPetite: Validation of a smartphone app-based tool for the remote measure of free-living subjective appetite. *British Journal of Nutrition* **2021**, pp. 1–30. https://doi.org/10.1017/S0007114521003512.
156. Franja, S.; Wahl, D.R.; Elliston, K.G.; Ferguson, S.G. Comfort eating: An observational study of affect in the hours immediately before, and after, snacking. *British Journal of Health Psychology* **2021**, *26*, 825–838. https://doi.org/10.1111/bjhp.12505.
157. Do, B.; Wang, S.D.; Courtney, J.B.; Dunton, G.F. Examining the day-level impact of physical activity on affect during the early months of the COVID-19 pandemic: An ecological momentary assessment study. *Psychology of Sport and Exercise* **2021**, *56*, 102010. https://doi.org/10.1016/j.psychsport.2021.102010.
158. Zani, A.; Lobbezoo, F.; Bracci, A.; Djukic, G.; Guarda-Nardini, L.; Favero, R.; Ferrari, M.; Aarab, G.; Manfredini, D. Smartphone-based evaluation of awake bruxism behaviours in a sample of healthy young adults: findings from two University centres. *Journal of Oral Rehabilitation* **2021**, *48*, 989–995. https://doi.org/10.1111/joor.13212.
159. Jensen, M.; George, M.J.; Russell, M.A.; Lippold, M.A.; Odgers, C.L. Daily Parent-Adolescent Digital Exchanges. *Research on Child and Adolescent Psychopathology* **2021**, *49*, 1125–1138. https://doi.org/10.1007/s10802-020-00765-x.
160. Zhaoyang, R.; Scott, S.B.; Martire, L.M.; Sliwinski, M.J. Daily social interactions related to daily performance on mobile cognitive tests among older adults. *PLoS ONE* **2021**, *16*, e0256583. https://doi.org/10.1371/journal.pone.0256583.
161. Xu, Y.; Towe, S.L.; Causey, S.T.; Meade, C.S. Using mobile health technologies to test the association of cocaine use with sexual desire and risky sexual behaviors among people with and without HIV who use illicit stimulants. *Drug and alcohol dependence* **2021**, *225*, 108744. https://doi.org/10.1016/j.drugalcdep.2021.108744.
162. Parrish, E.M.; Chalker, S.A.; Cano, M.; Moore, R.C.; Pinkham, A.E.; Harvey, P.D.; Joiner, T.; Lieberman, A.; Granholm, E.; Depp, C.A. Ecological momentary assessment of interpersonal theory of suicide constructs in people experiencing psychotic symptoms. *Journal of psychiatric research* **2021**, *140*, 496–503. https://doi.org/10.1016/j.jpsychires.2021.06.022.
163. Knapp, K.S.; Brick, T.R.; Bunce, S.C.; Deneke, E.; Cleveland, H.H. Daily meaningfulness among patients with opioid use disorder: Examining the role of social experiences during residential treatment and links with post-treatment relapse. *Addictive behaviors* **2021**, *119*, 106914. https://doi.org/10.1016/j.addbeh.2021.106914.
164. González-Barato, L.J.; Rubio, V.J.; Hernández, J.M.; Sánchez-Iglesias, I. PSIXPORT: Mobile App for Ecological Momentary Assessment of Psychological Dimensions in Sport Injury. *Frontiers in Psychology* **2021**, *12*, 697293. https://doi.org/10.3389/fpsyg.2021.697293.
165. Beres, L.K.; Mbabali, I.; Anok, A.; Katabalwa, C.; Mulamba, J.; Thomas, A.G.; Bugos, E.; Nakigozi, G.; Grabowski, M.K.; Chang, L.W. Mobile Ecological Momentary Assessment and Intervention and Health Behavior Change Among Adults in Rakai, Uganda: Pilot Randomized Controlled Trial. *JMIR Formative Research* **2021**, *5*, e22693. https://doi.org/10.2196/22693.
166. Blome, C.; Carlton, J.; Heesen, C.; Janssen, M.F.; Lloyd, A.; Otten, M.; Brazier, J. How to measure fluctuating impairments in people with MS: development of an ambulatory assessment version of the EQ-5D-5L in an exploratory study. *Quality of Life Research* **2021**, *30*, 2081–2096. https://doi.org/10.1007/s11136-021-02802-8.
167. Stull, S.W.; Bertz, J.W.; Panlilio, L.V.; Kowalczyk, W.J.; Phillips, K.A.; Moran, L.M.; Lin, J.L.; Vahabzadeh, M.; Finan, P.H.; Preston, K.L.; et al. I Feel Good?: Anhedonia Might Not Mean "Without Pleasure" for People Treated for Opioid Use Disorder. *Journal of abnormal psychology* **2021**, *130*, 537–549. https://doi.org/10.1037/abn0000674.
168. Santa Maria, D.; Padhye, N.; Businelle, M.; Yang, Y.; Jones, J.; Sims, A.; Lightfoot, M. Efficacy of a Just-in-Time Adaptive Intervention to Promote HIV Risk Reduction Behaviors Among Young Adults Experiencing Homelessness: Pilot Randomized Controlled Trial. *Journal of Medical Internet Research* **2021**, *23*, e26704. https://doi.org/10.2196/26704.
169. Sinvani, R.T.; Fogel-Grinvald, H.; Afek, A.; Ben-Avraham, R.; Davidov, A.; Cohen, N.B.; Ben Yehuda, A.; Nahum, M.; Gilboa, Y. Ecological Momentary Mood, Resilience, and Mental Health Status as Predictors of Quality of Life Among Young Adults Under Stress: A Structural Equation Modeling Analysis. *Frontiers in Psychiatry* **2021**, *12*, 672397. https://doi.org/10.3389/fpsyt.2021.672397.
170. Fulford, D.; Gard, D.E.; Mueser, K.T.; Mote, J.; Gill, K.; Leung, L.; Mow, J. Preliminary Outcomes of an Ecological Momentary Intervention for Social Functioning in Schizophrenia: Pre-Post Study of the Motivation and Skills Support App. *JMIR Mental Health* **2021**, *8*, e27475. https://doi.org/10.2196/27475.
171. Villegas, F.; Martínez-Borba, V.; Suso-Ribera, C.; Castilla, D.; Zaragoza, I.; García-Palacios, A.; Ferrer, C. Characterizing Breakthrough Cancer Pain Using Ecological Momentary Assessment with a Smartphone App: Feasibility and Clinical Findings. *International Journal of Environmental Research and Public Health* **2021**, *18*, 5991. https://doi.org/10.3390/ijerph18115991.

172. Fridberg, D.J.; Cao, D.; King, A.C. Alcohol Subjective Responses in Heavy Drinkers: Measuring Acute Effects in the Natural Environment Versus the Controlled Laboratory Setting. *Alcoholism, clinical and experimental research* **2021**, *45*, 1287–1297. https://doi.org/10.1111/acer.14616.
173. Abel, D.B.; Minor, K.S. Social Functioning in Schizophrenia: Comparing Laboratory-based Assessment with Real-world Measures. *Journal of psychiatric research* **2021**, *138*, 500–506. https://doi.org/10.1016/j.jpsychires.2021.04.039.
174. Arjmand, H.A.; Seabrook, E.; Bakker, D.; Rickard, N. Mental Health Consequences of Adversity in Australia: National Bushfires Associated With Increased Depressive Symptoms, While COVID-19 Pandemic Associated With Increased Symptoms of Anxiety. *Frontiers in Psychology* **2021**, *12*, 635158. https://doi.org/10.3389/fpsyg.2021.635158.
175. Yong, J.Y.Y.; Tong, E.M.W.; Liu, J.C.J. Meal-time Smartphone Use in an Obesogenic Environment: Two Longitudinal Observational Studies. *JMIR mHealth and uHealth* **2021**, *9*, e22929. https://doi.org/10.2196/22929.
176. Höller, I.; Stenzel, J.S.; Rath, D.; Forkmann, T. Listen to Your Heart–Ecological Momentary Assessment of Interoceptive Accuracy, Awareness and Sensibility: A Pilot Study. *International Journal of Environmental Research and Public Health* **2021**, *18*, 4893. https://doi.org/10.3390/ijerph18094893.
177. Markowski, K.L.; Smith, J.A.; Gauthier, G.R.; Harcey, S.R. Practical problems and positive experiences with ecological momentary assessment: reflections from people who use drugs. *The American journal of drug and alcohol abuse* **2021**, *47*, 311–318. https://doi.org/10.1080/00952990.2021.1910700.
178. Rose, K.M.; Coop Gordon, K.; Schlegel, E.C.; Mccall, M.; Gao, Y.; Ma, M.; Lenger, K.A.; Ko, E.; Wright, K.D.; Wang, H.; et al. Smarthealth technology study protocol to improve relationships between older adults with dementia and family caregivers. *Journal of Advanced Nursing* **2021**, *77*, 2519–2529. https://doi.org/10.1111/jan.14714.
179. Cobo, A.; Porras-Segovia, A.; Pérez-Rodríguez, M.M.; Artés-Rodríguez, A.; Barrigón, M.L.; Courtet, P.; Baca-García, E. Patients at high risk of suicide before and during a COVID-19 lockdown: ecological momentary assessment study. *BJPsych Open* **2021**, *7*, e82. https://doi.org/10.1192/bjo.2021.43.
180. Dowling, N.A.; Merkouris, S.S.; Spence, K. Ecological Momentary Assessment of the Relationship between Positive Outcome Expectancies and Gambling Behaviour. *Journal of Clinical Medicine* **2021**, *10*, 1709. https://doi.org/10.3390/jcm10081709.
181. Emodi-Perlman, A.; Manfredini, D.; Shalev, T.; Yevdayev, I.; Frideman-Rubin, P.; Bracci, A.; Arnias-Winocur, O.; Eli, I. Awake Bruxism—Single-Point Self-Report versus Ecological Momentary Assessment. *Journal of Clinical Medicine* **2021**, *10*, 1699. https://doi.org/10.3390/jcm10081699.
182. Betthauser, L.M.; Forster, J.E.; Bortz, A.; Penzenik, M.; Hernández, T.D.; Bahraini, N.; Brenner, L.A. Strength and awareness in action: Feasibility of a yoga-based intervention for post-acute mild TBI headaches among veterans. *Contemporary Clinical Trials Communications* **2021**, *22*, 100762. https://doi.org/10.1016/j.conctc.2021.100762.
183. Durand, D.; Strassnig, M.T.; Moore, R.C.; Depp, C.A.; Ackerman, R.A.; Pinkham, A.E.; Harvey, P.D. Self-reported Social Functioning and Social Cognition in Schizophrenia and Bipolar Disorder: Using Ecological Momentary Assessment to Identify the Origin of Bias. *Schizophrenia research* **2021**, *230*, 17–23. https://doi.org/10.1016/j.schres.2021.02.011.
184. Glista, D.; O'Hagan, R.; Van Eeckhoutte, M.; Lai, Y.; Scollie, S. The use of ecological momentary assessment to evaluate real-world aided outcomes with children. *International Journal of Audiology* **2021**, *60*, S68–S78. https://doi.org/10.1080/14992027.2021.1881629.
185. Villinger, K.; Wahl, D.R.; Schupp, H.T.; Renner, B. Memorable meals: The memory-experience gap in day-to-day experiences. *PLoS ONE* **2021**, *16*, e0249190. https://doi.org/10.1371/journal.pone.0249190.
186. Schwarz, S.; Martin, D.D.; Büssing, A.; Kulikova, O.; Krafft, H.; Gwiasda, M.; Hamideh Kerdar, S.; Fingerhut, I.; Jenetzky, E. Sociodemographic Characteristics and Interests of FeverApp Users. *International Journal of Environmental Research and Public Health* **2021**, *18*, 3121. https://doi.org/10.3390/ijerph18063121.
187. Wakaizumi, K.; Wu, B.; Huang, S.; Fan, L.; Shen, B.; Wu, B.; Zhang, J.; Baliki, M.N.; Apkarian, A.V.; Huang, L. Momentary pain assessments reveal benefits of endoscopic discectomy: a prospective cohort study. *Pain Reports* **2021**, *6*, e906. https://doi.org/10.1097/PR9.0000000000000906.
188. Webb, C.A.; Israel, E.S.; Belleau, E.; Appleman, L.; Forbes, E.E.; Pizzagalli, D.A. Mind-Wandering in Adolescents Predicts Worse Affect and Is Linked to Aberrant Default Mode Network-Salience Network Connectivity. *Journal of the American Academy of Child and Adolescent Psychiatry* **2021**, *60*, 377–387. https://doi.org/10.1016/j.jaac.2020.03.010.
189. Hawker, C.O.; Merkouris, S.S.; Youssef, G.J.; Dowling, N.A. A Smartphone-Delivered Ecological Momentary Intervention for Problem Gambling (GamblingLess: Curb Your Urge): Single-Arm Acceptability and Feasibility Trial. *Journal of Medical Internet Research* **2021**, *23*, e25786. https://doi.org/10.2196/25786.
190. Dubov, A.; Fraenkel, L.; Goldstein, Z.; Arroyo, H.; McKellar, D.; Shoptaw, S. Development of a Smartphone App to Predict and Improve the Rates of Suicidal Ideation Among Transgender Persons (TransLife): Qualitative Study. *Journal of Medical Internet Research* **2021**, *23*, e24023. https://doi.org/10.2196/24023.
191. Neff, A.J.; Lee, Y.; Metts, C.L.; Wong, A.W. Ecological Momentary Assessment of Social Interactions: Associations With Depression, Anxiety, Pain, and Fatigue in Individuals With Mild Stroke. *Archives of physical medicine and rehabilitation* **2021**, *102*, 395–405. https://doi.org/10.1016/j.apmr.2020.08.007.
192. Panza, E.; Olson, K.; Selby, E.A.; Wing, R.R. State versus trait weight, shape, and eating concerns: Disentangling influence on eating behaviors among sexual minority women. *Body image* **2021**, *36*, 107–116. https://doi.org/10.1016/j.bodyim.2020.10.010.

193. Bond, D.S.; Thomas, J.G.; Jones, D.B.; Schumacher, L.M.; Webster, J.; Evans, E.W.; Goldschmidt, A.B.; Vithiananthan, S. Ecological momentary assessment of gastrointestinal symptoms and risky eating behaviors in Roux-en-Y gastric bypass and sleeve gastrectomy patients. *Surgery for obesity and related diseases : official journal of the American Society for Bariatric Surgery* **2021**, *17*, 475–483. https://doi.org/10.1016/j.soard.2020.11.017.
194. Wang, S.B.; Coppersmith, D.D.L.; Kleiman, E.M.; Bentley, K.H.; Millner, A.J.; Fortgang, R.; Mair, P.; Dempsey, W.; Huffman, J.C.; Nock, M.K. A Pilot Study Using Frequent Inpatient Assessments of Suicidal Thinking to Predict Short-Term Postdischarge Suicidal Behavior. *JAMA Network Open* **2021**, *4*, e210591. https://doi.org/10.1001/jamanetworkopen.2021.0591.
195. Zhaoyang, R.; Sliwinski, M.J.; Martire, L.M.; Katz, M.J.; Scott, S.B. Features of Daily Social Interactions That Discriminate Between Older Adults With and Without Mild Cognitive Impairment. *The Journals of Gerontology: Series B* **2021**, p. gbab019. https://doi.org/10.1093/geronb/gbab019.
196. Maatoug, R.; Peiffer-Smadja, N.; Delval, G.; Brochu, T.; Pitrat, B.; Millet, B. Ecological Momentary Assessment Using Smartphones in Patients With Depression: Feasibility Study. *JMIR Formative Research* **2021**, *5*, e14179. https://doi.org/10.2196/14179.
197. Burchert, S.; Kerber, A.; Zimmermann, J.; Knaevelsrud, C. Screening accuracy of a 14-day smartphone ambulatory assessment of depression symptoms and mood dynamics in a general population sample: Comparison with the PHQ-9 depression screening. *PLoS ONE* **2021**, *16*, e0244955. https://doi.org/10.1371/journal.pone.0244955.
198. Dubad, M.; Elahi, F.; Marwaha, S. The Clinical Impacts of Mobile Mood-Monitoring in Young People With Mental Health Problems: The MeMO Study. *Frontiers in Psychiatry* **2021**, *12*, 687270. https://doi.org/10.3389/fpsyt.2021.687270.
199. Gratch, I.; Choo, T.H.; Galfalvy, H.; Keilp, J.G.; Itzhaky, L.; Mann, J.J.; Oquendo, M.A.; Stanley, B. Detecting suicidal thoughts: The power of ecological momentary assessment. *Depression and Anxiety* **2021**, *38*, 8–16. https://doi.org/10.1002/da.23043.
200. Dunn, C.C.; Stangl, E.; Oleson, J.; Smith, M.; Chipara, O.; Wu, Y.H. The Influence of Forced Social Isolation on the Auditory Ecology and Psychosocial Functions of Listeners with Cochlear Implants During COVID-19 Mitigation Efforts. *Ear and hearing* **2020**, *42*, 20–28. https://doi.org/10.1097/AUD.0000000000000991.
201. Rauschenberg, C.; Boecking, B.; Paetzold, I.; Schruers, K.; Schick, A.; van Amelsvoort, T.; Reininghaus, U. A Compassion-Focused Ecological Momentary Intervention for Enhancing Resilience in Help-Seeking Youth: Uncontrolled Pilot Study. *JMIR Mental Health* **2021**, *8*, e25650. https://doi.org/10.2196/25650.
202. Mazereel, V.; Vansteelandt, K.; Menne-Lothmann, C.; Decoster, J.; Derom, C.; Thiery, E.; Rutten, B.P.; Jacobs, N.; van Os, J.; Wichers, M.; et al. The complex and dynamic interplay between self-esteem, belongingness and physical activity in daily life: An experience sampling study in adolescence and young adulthood. *Mental Health and Physical Activity* **2021**, *21*, 100413. https://doi.org/10.1016/j.mhpa.2021.100413.
203. Naim, R.; Smith, A.; Chue, A.; Grassie, H.; Linke, J.; Dombek, K.; Shaughnessy, S.; McNeil, C.; Cardinale, E.; Agorsor, C.; et al. Using ecological momentary assessment to enhance irritability phenotyping in a transdiagnostic sample of youth. *Development and Psychopathology* **2021**, *33*, 1734–1746. https://doi.org/10.1017/S0954579421000717.
204. Hébert, E.T.; Suchting, R.; Ra, C.K.; Alexander, A.C.; Kendzor, D.E.; Vidrine, D.J.; Businelle, M.S. Predicting the first smoking lapse during a quit attempt: A machine learning approach. *Drug and alcohol dependence* **2021**, *218*, 108340. https://doi.org/10.1016/j.drugalcdep.2020.108340.
205. Sagui-Henson, S.J.; Radin, R.M.; Jhaveri, K.; Brewer, J.A.; Cohn, M.; Hartogensis, W.; Mason, A.E. Negative Mood and Food Craving Strength Among Women with Overweight: Implications for Targeting Mechanisms Using a Mindful Eating Intervention. *Mindfulness* **2021**, *12*, 2997–3010. https://doi.org/10.1007/s12671-021-01760-z.
206. Varsavsky, T.; Graham, M.S.; Canas, L.S.; Ganesh, S.; Capdevila Pujol, J.; Sudre, C.H.; Murray, B.; Modat, M.; Jorge Cardoso, M.; Astley, C.M.; et al. Detecting COVID-19 infection hotspots in England using large-scale self-reported data from a mobile application: a prospective, observational study. *The Lancet Public Health* **2021**, *6*, e21–e29. https://doi.org/10.1016/S2468-2667(20)30269-3.
207. Sobolev, M.; Vitale, R.; Wen, H.; Kizer, J.; Leeman, R.; Pollak, J.P.; Baumel, A.; Vadhan, N.P.; Estrin, D.; Muench, F. The Digital Marshmallow Test (DMT) Diagnostic and Monitoring Mobile Health App for Impulsive Behavior: Development and Validation Study. *JMIR mHealth and uHealth* **2021**, *9*, e25018. https://doi.org/10.2196/25018.
208. Doorley, J.D.; Goodman, F.R.; Disabato, D.J.; Kashdan, T.B.; Weinstein, J.S.; Shackman, A.J. The momentary benefits of positive events for individuals with elevated social anxiety. *Emotion (Washington, D.C.)* **2021**, *21*, 595–606. https://doi.org/10.1037/emo0000725.
209. Dančík, D.; Kasanova, Z.; Hajdúk, M.; Heretik, A. Attachment, Stress and Emotions in Daily Life: An Experience Sampling Study. *Studia Psychologica* **2021**, *63*, 323–336. https://doi.org/10.31577/sp.2021.04.830.
210. Cleveland, H.H.; Knapp, K.S.; Brick, T.R.; Russell, M.A.; Gajos, J.M.; Bunce, S.C. Effectiveness and utility of mobile device assessment of subjective craving during residential opioid dependence treatment. *Substance use & misuse* **2021**, *56*, 1284–1294. https://doi.org/10.1080/10826084.2021.1921808.
211. van Agteren, J.; Bartholomaeus, J.; Steains, E.; Lo, L.; Gerace, A. Using a Technology-Based Meaning and Purpose Intervention to Improve Well-being: A Randomised Controlled Study. *Journal of Happiness Studies* **2021**, *22*, 3571–3591. https://doi.org/10.1007/s10902-021-00383-4.
212. Brys, A.D.; Stifft, F.; Van Heugten, C.M.; Bossola, M.; Gambaro, G.; Lenaert, B. mHealth-based experience sampling method to identify fatigue in the context of daily life in haemodialysis patients. *Clinical Kidney Journal* **2021**, *14*, 245–254. https://doi.org/10.1093/ckj/sfaa124.

213. Zhang, Z.; Zhang, L.; Zheng, J.; Xiao, H.; Li, Z. COVID-19–Related Disruptions and Increased mHealth Emergency Use Intention: Experience Sampling Method Study. *JMIR mHealth and uHealth* **2020**, *8*, e20642. https://doi.org/10.2196/20642.
214. Kleiman, E.M.; Yeager, A.L.; Grove, J.L.; Kellerman, J.K.; Kim, J.S. Real-time Mental Health Impact of the COVID-19 Pandemic on College Students: Ecological Momentary Assessment Study. *JMIR Mental Health* **2020**, *7*, e24815. https://doi.org/10.2196/24815.
215. Xu, J.; Wu, Y.H.; Stangl, E.; Crukley, J.; Pentony, S.; Galster, J. Using Smartphone-Based Ecological Momentary Assessment in Audiology Research: The Participants' Perspective. *American Journal of Audiology* **2020**, *29*, 935–943. https://doi.org/10.1044/2020_AJA-20-00057.
216. Bartels, S.L.; van Knippenberg, R.J.M.; Köhler, S.; Ponds, R.W.; Myin-Germeys, I.; Verhey, F.R.J.; de Vugt, M.E. The necessity for sustainable intervention effects: lessons-learned from an experience sampling intervention for spousal carers of people with dementia. *Aging & Mental Health* **2020**, *24*, 2082–2093. https://doi.org/10.1080/13607863.2019.1647130.
217. Goldstein, S.P.; Thomas, J.G.; Foster, G.D.; Turner-McGrievy, G.; Butryn, M.L.; Herbert, J.D.; Martin, G.J.; Forman, E.M. Refining an algorithm-powered just-in-time adaptive weight control intervention: A randomized controlled trial evaluating model performance and behavioral outcomes. *Health informatics journal* **2020**, *26*, 2315–2331. https://doi.org/10.1177/1460458220902330.
218. Suffoletto, B.; Field, M.; Chung, T. Attentional and Approach Biases to Alcohol Cues among Young Adult Drinkers: An Ecological Momentary Assessment Study. *Experimental and clinical psychopharmacology* **2019**, p. 10.1037/pha0000343. https://doi.org/10.1037/pha0000343.
219. Mey, L.K.; Chmitorz, A.; Kurth, K.; Wenzel, M.; Kalisch, R.; Tüscher, O.; Kubiak, T. Increases of negative affect following daily hassles are not moderated by neuroticism: An ecological momentary assessment study. *Stress and Health* **2020**, *36*, 615–628. https://doi.org/10.1002/smi.2964.
220. Philip, P.; Dupuy, L.; Morin, C.M.; de Sevin, E.; Bioulac, S.; Taillard, J.; Serre, F.; Auriacombe, M.; Micoulaud-Franchi, J.A. Smartphone-Based Virtual Agents to Help Individuals With Sleep Concerns During COVID-19 Confinement: Feasibility Study. *Journal of Medical Internet Research* **2020**, *22*, e24268. https://doi.org/10.2196/24268.
221. Müller, L.R.F.; Gossmann, K.; Schmid, R.F.; Rosner, R.; Unterhitzenberger, J. A pilot study on ecological momentary assessment in asylum-seeking children and adolescents resettled to Germany: Investigating compliance, post-migration factors, and the relation between daily mood, sleep patterns, and mental health. *PLoS ONE* **2021**, *16*, e0246069. https://doi.org/10.1371/journal.pone.0246069.
222. Meisel, S.N.; Padovano, H.T.; Miranda, R. Combined Pharmacotherapy and Evidence-Based Psychosocial Cannabis Treatment for Youth and Selection of Cannabis-Using Friends. *Drug and alcohol dependence* **2021**, *225*, 108747. https://doi.org/10.1016/j.drugalcdep.2021.108747.
223. Jensen, M.; George, M.; Russell, M.A.; Lippold, M.; Odgers, C.L. Does Adolescent Digital Technology Use Detract from the Parent-Adolescent Relationship? *Journal of research on adolescence : the official journal of the Society for Research on Adolescence* **2021**, *31*, 469–481. https://doi.org/10.1111/jora.12618.
224. Depp, C.; Kamarsu, S.; Filip, T.F.; Parrish, E.M.; Harvey, P.D.; Granholm, E.; Chalker, S.; Moore, R.C.; Pinkham, A. Ecological Momentary Facial Emotion Recognition in Psychotic Disorders. *Psychological medicine* **2021**, pp. 1–9. https://doi.org/10.1017/S0033291720004419.
225. Mchugh, T.; Brown, K.A.; Daniel, S.J.; Balram, S.; Frigon, C. Parental Engagement of a Prototype Electronic Diary in an Ambulatory Setting Following Adenotonsillectomy in Children: A Prospective Cohort Study. *Children* **2021**, *8*, 559. https://doi.org/10.3390/children8070559.
226. Schmid, J.; Imbach, L.; Klaperski, S.; Sudeck, G. The Natural Environment of Physical Activity and Perceived Stress: The Mediating Role of Specific Recovery Experiences. *Frontiers in Sports and Active Living* **2021**, *3*, 706467. https://doi.org/10.3389/fspor.2021.706467.
227. Moitra, E.; Park, H.S.; Ben-Zeev, D.; Gaudiano, B.A. Using ecological momentary assessment for patients with psychosis post-hospitalization: Opportunities for mobilizing measurement-based care. *Psychiatric rehabilitation journal* **2021**, *44*, 43–50. https://doi.org/10.1037/prj0000417.
228. Jean, F.A.M.; Sibon, I.; Husky, M.; Couffinhal, T.; Swendsen, J. Feasibility and validity of Ecological Momentary Assessment in patients with acute coronary syndrome. *BMC Cardiovascular Disorders* **2020**, *20*, 499. https://doi.org/10.1186/s12872-020-01774-w.
229. Ebner-Priemer, U.W.; Mühlbauer, E.; Neubauer, A.B.; Hill, H.; Beier, F.; Santangelo, P.S.; Ritter, P.; Kleindienst, N.; Bauer, M.; Schmiedek, F.; et al. Digital phenotyping: towards replicable findings with comprehensive assessments and integrative models in bipolar disorders. *International Journal of Bipolar Disorders* **2020**, *8*, 35. https://doi.org/10.1186/s40345-020-00210-4.
230. Nicol, G.E.; Ricchio, A.R.; Metts, C.L.; Yingling, M.D.; Ramsey, A.T.; Schweiger, J.A.; Miller, J.P.; Lenze, E.J. A Smartphone-Based Technique to Detect Dynamic User Preferences for Tailoring Behavioral Interventions: Observational Utility Study of Ecological Daily Needs Assessment. *JMIR mHealth and uHealth* **2020**, *8*, e18609. https://doi.org/10.2196/18609.
231. Mackesy-Amiti, M.E.; Boodram, B.; Donenberg, G. Negative affect, affect-related impulsivity, and receptive syringe sharing among people who inject drugs. *Psychology of addictive behaviors : journal of the Society of Psychologists in Addictive Behaviors* **2020**, *34*, 734–744. https://doi.org/10.1037/adb0000590.
232. Wilson, N.J.; Chen, Y.W.; Mahoney, N.; Buchanan, A.; Marks, A.; Cordier, R. Experience sampling method and the everyday experiences of adults with intellectual disability: A feasibility study. *Journal of Applied Research in Intellectual Disabilities* **2020**, *33*, 1328–1339. https://doi.org/10.1111/jar.12753.

233. Toscano, F.; O'Donnell, E.; Broderick, J.E.; May, M.; Tucker, P.; Unruh, M.A.; Messina, G.; Casalino, L.P. How Physicians Spend Their Work Time: an Ecological Momentary Assessment. *Journal of General Internal Medicine* **2020**, *35*, 3166–3172. https://doi.org/10.1007/s11606-020-06087-4.
234. Minen, M.T.; Adhikari, S.; Padikkala, J.; Tasneem, S.; Bagheri, A.; Goldberg, E.; Powers, S.; Lipton, R.B. Smartphone-Delivered Progressive Muscle Relaxation for the Treatment of Migraine in Primary Care: A Randomized Controlled Trial. *Headache: The Journal of Head and Face Pain* **2020**, *60*, 2232–2246. https://doi.org/10.1111/head.14010.
235. Burke, L.A.; Naylor, G. Daily-Life Fatigue in Mild to Moderate Hearing Impairment: An Ecological Momentary Assessment Study. *Ear and Hearing* **2020**, *41*, 1518–1532. https://doi.org/10.1097/AUD.0000000000000888.
236. Galve Villa, M.; S Palsson, T.; Cid Royo, A.; R Bjarkam, C.; Boudreau, S.A. Digital Pain Mapping and Tracking in Patients With Chronic Pain: Longitudinal Study. *Journal of Medical Internet Research* **2020**, *22*, e21475. https://doi.org/10.2196/21475.
237. Gómez-Pérez, M.C.; García-Palacios, A.; Castilla, D.; Zaragozá, I.; Suso-Ribera, C. Brief Acceptance and Commitment Therapy for Fibromyalgia: Feasibility and Effectiveness of a Replicated Single-Case Design. *Pain Research and Management* **2020**, *2020*, 1–11. https://doi.org/10.1155/2020/7897268.
238. Bartels, S.L.; van Knippenberg, R.J.M.; Malinowsky, C.; Verhey, F.R.J.; de Vugt, M.E. Smartphone-Based Experience Sampling in People With Mild Cognitive Impairment: Feasibility and Usability Study. *JMIR Aging* **2020**, *3*, e19852. https://doi.org/10.2196/19852.
239. Scott, C.K.; Dennis, M.L.; Johnson, K.A.; Grella, C.E. A randomized clinical trial of smartphone self-managed recovery support Services. *Journal of substance abuse treatment* **2020**, *117*, 108089. https://doi.org/10.1016/j.jsat.2020.108089.
240. Papp, L.M.; Barringer, A.; Blumenstock, S.M.; Gu, P.; Blaydes, M.; Lam, J.; Kouros, C.D. Development and Acceptability of a Method to Investigate Prescription Drug Misuse in Daily Life: Ecological Momentary Assessment Study. *JMIR mHealth and uHealth* **2020**, *8*, e21676. https://doi.org/10.2196/21676.
241. Campbell, L.M.; Paolillo, E.W.; Heaton, A.; Tang, B.; Depp, C.A.; Granholm, E.; Heaton, R.K.; Swendsen, J.; Moore, D.J.; Moore, R.C. Daily Activities Related to Mobile Cognitive Performance in Middle-Aged and Older Adults: An Ecological Momentary Cognitive Assessment Study. *JMIR mHealth and uHealth* **2020**, *8*, e19579. https://doi.org/10.2196/19579.
242. Suso-Ribera, C.; Castilla, D.; Zaragozá, I.; Mesas, ; Server, A.; Medel, J.; García-Palacios, A. Telemonitoring in Chronic Pain Management Using Smartphone Apps: A Randomized Controlled Trial Comparing Usual Assessment against App-Based Monitoring with and without Clinical Alarms. *International Journal of Environmental Research and Public Health* **2020**, *17*, 6568. https://doi.org/10.3390/ijerph17186568.
243. Schneider, S.; Junghaenel, D.U.; Gutsche, T.; Mak, H.W.; Stone, A.A. Comparability of Emotion Dynamics Derived From Ecological Momentary Assessments, Daily Diaries, and the Day Reconstruction Method: Observational Study. *Journal of Medical Internet Research* **2020**, *22*, e19201. https://doi.org/10.2196/19201.
244. Wu, Y.H.; Stangl, E.; Chipara, O.; Zhang, X. Test-retest Reliability of Ecological Momentary Assessment in Audiology Research. *Journal of the American Academy of Audiology* **2020**, *31*, 599–612. https://doi.org/10.1055/s-0040-1717066.
245. Mackesy-Amiti, M.E.; Donenberg, G. Negative affect and emotion dysregulation among people who inject drugs: An ecological momentary assessment study. *Psychology of addictive behaviors : journal of the Society of Psychologists in Addictive Behaviors* **2020**, *34*, 650–659. https://doi.org/10.1037/adb0000577.
246. Golan, D.; Sagiv, S.; Glass-Marmor, L.; Miller, A. Mobile phone-based e-diary for assessment and enhancement of medications adherence among patients with multiple sclerosis. *Multiple Sclerosis Journal - Experimental, Translational and Clinical* **2020**, *6*, 2055217320939309. https://doi.org/10.1177/2055217320939309.
247. Fatori, D.; Argeu, A.; Brentani, H.; Chiesa, A.; Fracolli, L.; Matijasevich, A.; Miguel, E.C.; Polanczyk, G. Maternal Parenting Electronic Diary in the Context of a Home Visit Intervention for Adolescent Mothers in an Urban Deprived Area of São Paulo, Brazil: Randomized Controlled Trial. *JMIR mHealth and uHealth* **2020**, *8*, e13686. https://doi.org/10.2196/13686.
248. Yang, Y.S.; Ryu, G.W.; Delespaul, P.A.E.G.; Choi, M. Psychometric Properties of the Korean Version of the PsyMate Scale Using a Smartphone App: Ecological Momentary Assessment Study. *JMIR mHealth and uHealth* **2020**, *8*, e17926. https://doi.org/10.2196/17926.
249. Fakih El Khoury, C.; Crutzen, R.; Schols, J.M.G.A.; Halfens, R.J.G.; Karavetian, M. A Dietary Mobile App for Patients Undergoing Hemodialysis: Prospective Pilot Study to Improve Dietary Intakes. *Journal of Medical Internet Research* **2020**, *22*, e17817. https://doi.org/10.2196/17817.
250. Panza, E.; Olson, K.; Goldstein, C.M.; Selby, E.A.; Lillis, J. Characterizing Lifetime and Daily Experiences of Weight Stigma among Sexual Minority Women with Overweight and Obesity: A Descriptive Study. *International Journal of Environmental Research and Public Health* **2020**, *17*, 4892. https://doi.org/10.3390/ijerph17134892.
251. Hallensleben, N.; Glaesmer, H.; Forkmann, T.; Rath, D.; Strauss, M.; Kersting, A.; Spangenberg, L. How Is the Presence of Company Related to Thwarted Belongingness in Real Time? Taking a Closer Look at the Conceptualization of the Construct of the Interpersonal Theory of Suicide. *International Journal of Environmental Research and Public Health* **2020**, *17*, 4873. https://doi.org/10.3390/ijerph17134873.
252. Nichols, M.; Miller, S.; Treiber, F.; Ruggiero, K.; Dawley, E.; Teufel II, R. Patient and Parent Perspectives on Improving Pediatric Asthma Self-Management Through a Mobile Health Intervention: Pilot Study. *JMIR Formative Research* **2020**, *4*, e15295. https://doi.org/10.2196/15295.

253. Giurgiu, M.; Plotnikoff, R.C.; Nigg, C.R.; Koch, E.D.; Ebner-Priemer, U.W.; Reichert, M. Momentary mood predicts upcoming real-life sedentary behavior. *Scandinavian Journal of Medicine & Science in Sports* **2020**, *30*, 1276–1286. https://doi.org/10.1111/sms.13652.
254. Stenzel, J.S.; Höller, I.; Rath, D.; Hallensleben, N.; Spangenberg, L.; Glaesmer, H.; Forkmann, T. Do Feelings of Defeat and Entrapment Change over Time? An Investigation of the Integrated Motivational—Volitional Model of Suicidal Behaviour Using Ecological Momentary Assessments. *International Journal of Environmental Research and Public Health* **2020**, *17*, 4685. https://doi.org/10.3390/ijerph17134685.
255. Katapally, T.R.; Chu, L.M. Digital epidemiological and citizen science methodology to capture prospective physical activity in free-living conditions: a SMART Platform study. *BMJ Open* **2020**, *10*, e036787. https://doi.org/10.1136/bmjopen-2020-036787.
256. Swendeman, D.; Sumstine, S.; Brink, A.; Mindry, D.; Medich, M.; Russell, M. Smartphone Self-Monitoring by Young Adolescents and Parents to Assess and Improve Family Functioning: Qualitative Feasibility Study. *JMIR Formative Research* **2020**, *4*, e15777. https://doi.org/10.2196/15777.
257. Villinger, K.; Wahl, D.R.; König, L.M.; Ziesemer, K.; Butscher, S.; Müller, J.; Reiterer, H.; Schupp, H.T.; Renner, B. Do We Know What We Enjoy? Accuracy of Forecasted Eating Happiness. *Frontiers in Psychology* **2020**, *11*, 1187. https://doi.org/10.3389/fpsyg.2020.01187.
258. Negishi, K.; Sekiguchi, T. Individual traits that influence the frequency and emotional characteristics of involuntary musical imagery: An experience sampling study. *PLoS ONE* **2020**, *15*, e0234111. https://doi.org/10.1371/journal.pone.0234111.
259. McNaughton, S.A.; Pendergast, F.J.; Worsley, A.; Leech, R.M. Eating occasion situational factors and sugar-sweetened beverage consumption in young adults. *International Journal of Behavioral Nutrition and Physical Activity* **2020**, *17*, 71. https://doi.org/10.1186/s12966-020-00975-y.
260. Nauta, M.H.; aan het Rot, M.; Schut, H.; Stroebe, M. Homesickness in social context: An ecological momentary assessment study among 1st-year university students. *International Journal of Psychology* **2020**, *55*, 392–397. https://doi.org/10.1002/ijop.12586.
261. Wray, T.B.; Monti, P.M. Characteristics of Sex Events, Partners, and Motivations and Their Associations with HIV-Risk Behavior in a Daily Diary Study of High-Risk Men Who Have Sex with Men (MSM). *AIDS and Behavior* **2020**, *24*, 1851–1864. https://doi.org/10.1007/s10461-019-02760-w.
262. Yang, Y.S.; Ryu, G.W.; Park, C.G.; Yeom, I.; Shim, K.W.; Choi, M. Mood and Stress Evaluation of Adult Patients With Moyamoya Disease in Korea: Ecological Momentary Assessment Method Using a Mobile Phone App. *JMIR mHealth and uHealth* **2020**, *8*, e17034. https://doi.org/10.2196/17034.
263. Verveer, I.; Remmerswaal, D.; Jongerling, J.; van der Veen, F.M.; Franken, I.H.A. No effect of repetitive tDCS on daily smoking behaviour in light smokers: A placebo controlled EMA study. *PLoS ONE* **2020**, *15*, e0233414. https://doi.org/10.1371/journal.pone.0233414.
264. Kumar, A.; Wang, M.; Riehm, A.; Yu, E.; Smith, T.; Kaplin, A. An Automated Mobile Mood Tracking Technology (Mood 24/7): Validation Study. *JMIR Mental Health* **2020**, *7*, e16237. https://doi.org/10.2196/16237.
265. Sicorello, M.; Dieckmann, L.; Moser, D.; Lux, V.; Luhmann, M.; Schlotz, W.; Kumsta, R. Oxytocin and the stress buffering effect of social company: a genetic study in daily life. *Social Cognitive and Affective Neuroscience* **2020**, *15*, 293–301. https://doi.org/10.1093/scan/nsaa034.
266. Davies, A.; Mueller, J.; Hennings, J.; Caress, A.L.; Jay, C. Recommendations for Developing Support Tools With People Suffering From Chronic Obstructive Pulmonary Disease: Co-Design and Pilot Testing of a Mobile Health Prototype. *JMIR Human Factors* **2020**, *7*, e16289. https://doi.org/10.2196/16289.
267. Hsiang, E.; Offer, C.; Prescott, M.; Rodriguez, A.; Behar, E.; Matheson, T.; Santa Maria, D.; Santos, G.M. Bridging the Digital Divide Among Racial and Ethnic Minority Men Who Have Sex With Men to Reduce Substance Use and HIV Risk: Mixed Methods Feasibility Study. *JMIR mHealth and uHealth* **2020**, *8*, e15282. https://doi.org/10.2196/15282.
268. Shrier, L.A.; Burke, P.J.; Parker, S.; Edwards, R.; Jonestrask, C.; Pluhar, E.; Harris, S.K. Development and pilot testing of a counseling-plus-mHealth intervention to reduce risk for pregnancy and sexually transmitted infection in young women with depression. *mHealth* **2020**, *6*, 17. https://doi.org/10.21037/mhealth.2019.11.05.
269. Lancaster, C.; Koychev, I.; Blane, J.; Chinner, A.; Wolters, L.; Hinds, C. Evaluating the Feasibility of Frequent Cognitive Assessment Using the Mezurio Smartphone App: Observational and Interview Study in Adults With Elevated Dementia Risk. *JMIR mHealth and uHealth* **2020**, *8*, e16142. https://doi.org/10.2196/16142.
270. Colonna, A.; Lombardo, L.; Siciliani, G.; Bracci, A.; Guarda-Nardini, L.; Djukic, G.; Manfredini, D. Smartphone-based application for EMA assessment of awake bruxism: compliance evaluation in a sample of healthy young adults. *Clinical Oral Investigations* **2020**, *24*, 1395–1400. https://doi.org/10.1007/s00784-019-03098-2.
271. Prince, M.A.; Collins, R.L.; Wilson, S.D.; Vincent, P.C. A Preliminary Test of a Brief Intervention to Lessen Young-Adults' Cannabis Use: Episode-level Smartphone Data Highlights the Role of Protective Behavioral Strategies and Exercise. *Experimental and clinical psychopharmacology* **2020**, *28*, 150–156. https://doi.org/10.1037/pha0000301.
272. Burke, L.E.; Sereika, S.M.; Parmanto, B.; Beatrice, B.; Cajita, M.; Loar, I.; Pulantara, I.W.; Wang, Y.; Kariuki, J.; Yu, Y.; et al. The SMARTER Trial: Design of a trial testing tailored mHealth feedback to impact self-monitoring of diet, physical activity, and weight. *Contemporary clinical trials* **2020**, *91*, 105958. https://doi.org/10.1016/j.cct.2020.105958.

273. NeCamp, T.; Sen, S.; Frank, E.; Walton, M.A.; Ionides, E.L.; Fang, Y.; Tewari, A.; Wu, Z. Assessing Real-Time Moderation for Developing Adaptive Mobile Health Interventions for Medical Interns: Micro-Randomized Trial. *Journal of Medical Internet Research* **2020**, *22*, e15033. https://doi.org/10.2196/15033.
274. Kimhy, D.; Lister, A.; Liu, Y.; Vakhrusheva, J.; Delespaul, P.; Malaspina, D.; Ospina, L.H.; Mittal, V.A.; Gross, J.J.; Wang, Y. The impact of emotion awareness and regulation on psychotic symptoms during daily functioning. *NPJ Schizophrenia* **2020**, *6*, 7. https://doi.org/10.1038/s41537-020-0096-6.
275. Forster, S.D.; Gauggel, S.; Petershofer, A.; Völzke, V.; Mainz, V. Ecological Momentary Assessment in Patients With an Acquired Brain Injury: A Pilot Study on Compliance and Fluctuations. *Frontiers in Neurology* **2020**, *11*, 115. https://doi.org/10.3389/fneur.2020.00115.
276. Hébert, E.T.; Ra, C.K.; Alexander, A.C.; Helt, A.; Moisiuc, R.; Kendzor, D.E.; Vidrine, D.J.; Funk-Lawler, R.K.; Businelle, M.S. A Mobile Just-in-Time Adaptive Intervention for Smoking Cessation: Pilot Randomized Controlled Trial. *Journal of Medical Internet Research* **2020**, *22*, e16907. https://doi.org/10.2196/16907.
277. Goldschmidt, A.B.; Evans, E.W.; Saletin, J.M.; O'Sullivan, K.; Koren, D.; Engel, S.G.; Haedt-Matt, A. Naturalistic, multimethod exploratory study of sleep duration and quality as predictors of dysregulated eating in youth with overweight and obesity. *Appetite* **2020**, *146*, 104521. https://doi.org/10.1016/j.appet.2019.104521.
278. Di Fraia, M.; Tripodi, S.; Arasi, S.; Dramburg, S.; Castelli, S.; Villalta, D.; Buzzulini, F.; Sfika, I.; Villella, V.; Potapova, E.; et al. Adherence to Prescribed E-Diary Recording by Patients With Seasonal Allergic Rhinitis: Observational Study. *Journal of Medical Internet Research* **2020**, *22*, e16642. https://doi.org/10.2196/16642.
279. Rupp, C.; Gühne, D.; Falke, C.; Doebler, P.; Andor, F.; Buhlmann, U. Comparing effects of detached mindfulness and cognitive restructuring in obsessive-compulsive disorder using ecological momentary assessment. *Clinical Psychology & Psychotherapy* **2020**, *27*, 193–202. https://doi.org/10.1002/cpp.2418.
280. Granholm, E.; Holden, J.L.; Mikhael, T.; Link, P.C.; Swendsen, J.; Depp, C.; Moore, R.C.; Harvey, P.D. What Do People With Schizophrenia Do All Day? Ecological Momentary Assessment of Real-World Functioning in Schizophrenia. *Schizophrenia Bulletin* **2019**, p. sbz070. https://doi.org/10.1093/schbul/sbz070.
281. Evans, W.E.; Raynor, H.A.; Howie, W.; Lipton, R.B.; Thomas, G.J.; Wing, R.R.; Pavlovic, J.; Farris, S.G.; Bond, D.S. Associations between lifestyle intervention-related changes in dietary targets and migraine headaches among women in the Women's Health and Migraine (WHAM) randomized controlled trial. *Obesity Science & Practice* **2020**, *6*, 119–125. https://doi.org/10.1002/osp4.376.
282. van Genugten, C.R.; Schuurmans, J.; Lamers, F.; Riese, H.; Penninx, B.W.J.H.; Schoevers, R.A.; Riper, H.M.; Smit, J.H. Experienced Burden of and Adherence to Smartphone-Based Ecological Momentary Assessment in Persons with Affective Disorders. *Journal of Clinical Medicine* **2020**, *9*, 322. https://doi.org/10.3390/jcm9020322.
283. Wahl, D.R.; Villinger, K.; Blumenschein, M.; König, L.M.; Ziesemer, K.; Sproesser, G.; Schupp, H.T.; Renner, B. Why We Eat What We Eat: Assessing Dispositional and In-the-Moment Eating Motives by Using Ecological Momentary Assessment. *JMIR mHealth and uHealth* **2020**, *8*, e13191. https://doi.org/10.2196/13191.
284. Waring, J.J.; Hébert, E.T.; Alexander, A.C.; Kendzor, D.E.; Businelle, M.S. Evaluating the influences of social support and smoking cues on daily smoking abstinence among socioeconomically disadvantaged adults. *Addictive behaviors* **2020**, *100*, 106107. https://doi.org/10.1016/j.addbeh.2019.106107.
285. Duif, M.; Thewissen, V.; Wouters, S.; Lechner, L.; Jacobs, N. Associations between affect and alcohol consumption in adults: an ecological momentary assessment study. *The American Journal of Drug and Alcohol Abuse* **2020**, *46*, 88–97. https://doi.org/10.1080/00952990.2019.1635606.
286. Colombo, D.; Suso-Ribera, C.; Fernández-Álvarez, J.; Cipresso, P.; Garcia-Palacios, A.; Riva, G.; Botella, C. Affect Recall Bias: Being Resilient by Distorting Reality. *Cognitive Therapy and Research* **2020**, *44*, 906–918. https://doi.org/10.1007/s10608-020-10122-3.
287. Ludwigs, K.; Lucas, R.; Veenhoven, R.; Richter, D.; Arends, L. Can Happiness Apps Generate Nationally Representative Datasets? - a Case Study Collecting Data on People's Happiness Using the German Socio-Economic Panel. *Applied Research in Quality of Life* **2020**, *15*, 1135–1149. https://doi.org/10.1007/s11482-019-09723-2.
288. Schnepper, R.; Reichenberger, J.; Blechert, J. Being My Own Companion in Times of Social Isolation – A 14-Day Mobile Self-Compassion Intervention Improves Stress Levels and Eating Behavior. *Frontiers in Psychology* **2020**, *11*, 595806. https://doi.org/10.3389/fpsyg.2020.595806.
289. Ma, R.; Kelly, A.C. The fragility of perceived social rank following exercise in anorexia nervosa: an ecological momentary assessment study of shame and pride. *Eating and Weight Disorders - Studies on Anorexia, Bulimia and Obesity* **2020**, *25*, 1601–1607. https://doi.org/10.1007/s40519-019-00797-3.
290. Drott, J.; Fomichov, V.; Börjeson, S.; Berterö, C. Sense of coherence and health-related quality of life in patients with neurotoxicity after cancer chemotherapy: Assessment from a real-time mobile phone–based system. *Psycho-Oncology* **2020**, *29*, 107–113. https://doi.org/10.1002/pon.5243.
291. Kaufmann, K.; Peil, C. The mobile instant messaging interview (MIMI): Using WhatsApp to enhance self-reporting and explore media usage in situ. *Mobile Media & Communication* **2020**, *8*, 229–246. https://doi.org/10.1177/2050157919852392.
292. Mulvaney, S.A.; Vaala, S.E.; Carroll, R.B.; Williams, L.K.; Lybarger, C.K.; Schmidt, D.C.; Dietrich, M.S.; Laffel, L.M.; Hood, K.K. A mobile app identifies momentary psychosocial and contextual factors related to mealtime self-management in adolescents with type 1 diabetes. *Journal of the American Medical Informatics Association : JAMIA* **2019**, *26*, 1627–1631. https://doi.org/10.1093/jamia/ocz147.

293. Roefs, A.; Boh, B.; Spanakis, G.; Nederkoorn, C.; Lemmens, L.H.J.M.; Jansen, A. Food craving in daily life: comparison of overweight and normal-weight participants with ecological momentary assessment. *Journal of Human Nutrition and Dietetics* **2019**, *32*, 765–774. https://doi.org/10.1111/jhn.12693.
294. Spangenberg, L.; Glaesmer, H.; Hallensleben, N.; Rath, D.; Forkmann, T. (In)stability of Capability for Suicide in Psychiatric Inpatients: Longitudinal Assessment Using Ecological Momentary Assessments. *Suicide and Life-Threatening Behavior* **2019**, *49*, 1560–1572. https://doi.org/10.1111/sltb.12547.
295. Cormack, F.; McCue, M.; Taptiklis, N.; Skirrow, C.; Glazer, E.; Panagopoulos, E.; van Schaik, T.A.; Fehnert, B.; King, J.; Barnett, J.H. Wearable Technology for High-Frequency Cognitive and Mood Assessment in Major Depressive Disorder: Longitudinal Observational Study. *JMIR Mental Health* **2019**, *6*, e12814. https://doi.org/10.2196/12814.
296. Santoro, A.; Delussi, M.; Leone, M.; Miscio, A.M.; De Rocco, L.; Leo, G.; De Tommaso, M. Effects of Botulinum Toxin on Migraine Attack Features in Chronic Migraine: A Six-Month Open-Label Observation Study through Electronic Diary Smartphone Application. *Toxins* **2019**, *11*, 668. https://doi.org/10.3390/toxins11110668.
297. Jensen, M.; George, M.; Russell, M.; Odgers, C. Young Adolescents' Digital Technology Use and Mental Health Symptoms: Little Evidence of Longitudinal or Daily Linkages. *Clinical psychological science : a journal of the Association for Psychological Science* **2019**, *7*, 1416–1433. https://doi.org/10.1177/2167702619859336.
298. Small, B.J.; Jim, H.S.L.; Eisel, S.L.; Jacobsen, P.B.; Scott, S.B. Cognitive Performance of Breast Cancer Survivors in Daily Life: Role of Fatigue and Depressed Mood. *Psycho-oncology* **2019**, *28*, 2174–2180. https://doi.org/10.1002/pon.5203.
299. Rupp, C.; Falke, C.; Gühne, D.; Doebler, P.; Andor, F.; Buhlmann, U. A study on treatment sensitivity of ecological momentary assessment in obsessive–compulsive disorder. *Clinical Psychology & Psychotherapy* **2019**, *26*, 695–706. https://doi.org/10.1002/cpp.2392.
300. Sufrinko, A.M.; Howie, E.K.; Charek, D.B.; Elbin, R.J.; Collins, M.W.; Kontos, A.P. Mobile Ecological Momentary Assessment of Postconcussion Symptoms and Recovery Outcomes. *The Journal of Head Trauma Rehabilitation* **2019**, *34*, E40. https://doi.org/10.1097/HTR.0000000000000474.
301. Meule, A.; Richard, A.; Dinic, R.; Blechert, J. Effects of a Smartphone-Based Approach-Avoidance Intervention on Chocolate Craving and Consumption: Randomized Controlled Trial. *JMIR mHealth and uHealth* **2019**, *7*, e12298. https://doi.org/10.2196/12298.
302. Fridberg, D.J.; Faria, J.; Cao, D.; King, A.C. Real-Time Mobile Monitoring of Drinking Episodes in Young Adult Heavy Drinkers: Development and Comparative Survey Study. *JMIR mHealth and uHealth* **2019**, *7*, e13765. https://doi.org/10.2196/13765.
303. Pryss, R.; John, D.; Schlee, W.; Schlotz, W.; Schobel, J.; Kraft, R.; Spiliopoulou, M.; Langguth, B.; Reichert, M.; O'Rourke, T.; et al. Exploring the Time Trend of Stress Levels While Using the Crowdsensing Mobile Health Platform, TrackYourStress, and the Influence of Perceived Stress Reactivity: Ecological Momentary Assessment Pilot Study. *JMIR mHealth and uHealth* **2019**, *7*, e13978. https://doi.org/10.2196/13978.
304. Perski, O.; Naughton, F.; Garnett, C.; Blandford, A.; Beard, E.; West, R.; Michie, S. Do Daily Fluctuations in Psychological and App-Related Variables Predict Engagement With an Alcohol Reduction App? A Series of N-Of-1 Studies. *JMIR mHealth and uHealth* **2019**, *7*, e14098. https://doi.org/10.2196/14098.
305. Yang, Y.S.; Ryu, G.W.; Choi, M. Factors Associated with Daily Completion Rates in a Smartphone-Based Ecological Momentary Assessment Study. *Healthcare Informatics Research* **2019**, *25*, 332–337. https://doi.org/10.4258/hir.2019.25.4.332.
306. Carpenter, R.W.; Lane, S.P.; Bruehl, S.; Trull, T.J. Concurrent and lagged associations of prescription opioid use with pain and negative affect in the daily lives of chronic pain patients. *Journal of consulting and clinical psychology* **2019**, *87*, 872–886. https://doi.org/10.1037/ccp0000402.
307. Turner-McGrievy, G.M.; Dunn, C.G.; Wilcox, S.; Boutté, A.K.; Hutto, B.; Hoover, A.; Muth, E. Defining Adherence to Mobile Dietary Self-Monitoring and Assessing Tracking Over Time: Tracking at Least Two Eating Occasions per Day Is Best Marker of Adherence within Two Different Mobile Health Randomized Weight Loss Interventions. *Journal of the Academy of Nutrition and Dietetics* **2019**, *119*, 1516–1524. https://doi.org/10.1016/j.jand.2019.03.012.
308. Van Dam, L.; Rietstra, S.; Van der Drift, E.; Stams, G.J.J.M.; Van der Mei, R.; Mahfoud, M.; Popma, A.; Schlossberg, E.; Pentland, A.; Reid, T.G. Can an Emoji a Day Keep the Doctor Away? An Explorative Mixed-Methods Feasibility Study to Develop a Self-Help App for Youth With Mental Health Problems. *Frontiers in Psychiatry* **2019**, *10*, 593. https://doi.org/10.3389/fpsyt.2019.00593.
309. Þórarinsdóttir, H.; Faurholt-Jepsen, M.; Ullum, H.; Frost, M.; Bardram, J.E.; Kessing, L.V. The Validity of Daily Self-Assessed Perceived Stress Measured Using Smartphones in Healthy Individuals: Cohort Study. *JMIR mHealth and uHealth* **2019**, *7*, e13418. https://doi.org/10.2196/13418.
310. Bailon, C.; Damas, M.; Pomares, H.; Sanabria, D.; Perakakis, P.; Goicoechea, C.; Banos, O. Smartphone-Based Platform for Affect Monitoring through Flexibly Managed Experience Sampling Methods. *Sensors (Basel, Switzerland)* **2019**, *19*, 3430. https://doi.org/10.3390/s19153430.
311. Smith, A.R.; Kircanski, K.; Brotman, M.A.; Do, Q.B.; Subar, A.R.; Silk, J.S.; Engel, S.; Crosby, R.D.; Harrewijn, A.; White, L.K.; et al. Advancing clinical neuroscience through enhanced tools: Pediatric social anxiety as an example. *Depression and Anxiety* **2019**, *36*, 701–711. https://doi.org/10.1002/da.22937.
312. Wenzel, M.; Geelen, A.; Wolters, M.; Hebestreit, A.; Van Laerhoven, K.; Lakerveld, J.; Andersen, L.F.; van't Veer, P.; Kubiak, T. The Role of Self-Control and the Presence of Enactment Models on Sugar-Sweetened Beverage Consumption: A Pilot Study. *Frontiers in Psychology* **2019**, *10*, 1511. https://doi.org/10.3389/fpsyg.2019.01511.

313. Götz, F.M.; Stieger, S.; Reips, U.D. The Emergence and Volatility of Homesickness in Exchange Students Abroad: A Smartphone-Based Longitudinal Study. *Environment and Behavior* **2019**, *51*, 689–716. https://doi.org/10.1177/0013916518754610.
314. Scholz, C.; Doré, B.P.; Cooper, N.; Falk, E.B. Neural Valuation of Anti-Drinking Campaigns and Risky Peer Influence in Daily Life. *Health psychology : official journal of the Division of Health Psychology, American Psychological Association* **2019**, *38*, 658–667. https://doi.org/10.1037/hea0000732.
315. Wu, Y.H.; Stangl, E.; Chipara, O.; Hasan, S.S.; DeVries, S.; Oleson, J. Efficacy and Effectiveness of Advanced Hearing Aid Directional and Noise Reduction Technologies for Older Adults with Mild to Moderate Hearing Loss. *Ear and hearing* **2019**, *40*, 805–822. https://doi.org/10.1097/AUD.0000000000000672.
316. Slavish, D.C.; Scaglione, N.M.; Hultgren, B.A.; Turrisi, R. An Ecological Momentary Assessment of Affect, Mental Health Symptoms, and Decisions to Drink Among First-Year College Women: A Pilot Study. *Prevention science : the official journal of the Society for Prevention Research* **2019**, *20*, 753–764. https://doi.org/10.1007/s11121-018-0966-6.
317. De Vuyst, H.J.; Dejonckheere, E.; Van der Gucht, K.; Kuppens, P. Does repeatedly reporting positive or negative emotions in daily life have an impact on the level of emotional experiences and depressive symptoms over time? *PLoS ONE* **2019**, *14*, e0219121. https://doi.org/10.1371/journal.pone.0219121.
318. Paterson, C. An ecological momentary assessment of self-management in prostate cancer survivors. *Journal of Cancer Survivorship* **2019**, *13*, 364–373. https://doi.org/10.1007/s11764-019-00758-w.
319. Smelror, R.E.; Bless, J.J.; Hugdahl, K.; Agartz, I. Feasibility and Acceptability of Using a Mobile Phone App for Characterizing Auditory Verbal Hallucinations in Adolescents With Early-Onset Psychosis: Exploratory Study. *JMIR Formative Research* **2019**, *3*, e13882. https://doi.org/10.2196/13882.
320. Schultchen, D.; Reichenberger, J.; Mittl, T.; Weh, T.R.M.; Smyth, J.M.; Blechert, J.; Pollatos, O. Bidirectional relationship of stress and affect with physical activity and healthy eating. *British Journal of Health Psychology* **2019**, *24*, 315–333. https://doi.org/10.1111/bjhp.12355.
321. Pentikäinen, S.; Tanner, H.; Karhunen, L.; Kolehmainen, M.; Poutanen, K.; Pennanen, K. Mobile Phone App for Self-Monitoring of Eating Rhythm: Field Experiment. *JMIR mHealth and uHealth* **2019**, *7*, e11490. https://doi.org/10.2196/11490.
322. Triantafillou, S.; Saeb, S.; Lattie, E.G.; Mohr, D.C.; Kording, K.P. Relationship Between Sleep Quality and Mood: Ecological Momentary Assessment Study. *JMIR Mental Health* **2019**, *6*, e12613. https://doi.org/10.2196/12613.
323. Bangerter, A.; Manyakov, N.V.; Lewin, D.; Boice, M.; Skalkin, A.; Jagannatha, S.; Chatterjee, M.; Dawson, G.; Goodwin, M.S.; Hendren, R.; et al. Caregiver Daily Reporting of Symptoms in Autism Spectrum Disorder: Observational Study Using Web and Mobile Apps. *JMIR Mental Health* **2019**, *6*, e11365. https://doi.org/10.2196/11365.
324. Greb, F.; Steffens, J.; Schlotz, W. Modeling Music-Selection Behavior in Everyday Life: A Multilevel Statistical Learning Approach and Mediation Analysis of Experience Sampling Data. *Frontiers in Psychology* **2019**, *10*, 390. https://doi.org/10.3389/fpsyg.2019.00390.
325. Heller, A.S.; Fox, A.S.; Davidson, R.J. Parsing Affective Dynamics to Identify Risk for Mood and Anxiety Disorders. *Emotion (Washington, D.C.)* **2019**, *19*, 283–291. https://doi.org/10.1037/emo0000440.
326. Andorko, N.D.; Rakhshan-Rouhakhtar, P.; Hinkle, C.; Mittal, V.A.; McAllister, M.; DeVylder, J.; Schiffman, J. Assessing validity of retrospective recall of physical activity in individuals with psychosis-like experiences. *Psychiatry research* **2019**, *273*, 211–217. https://doi.org/10.1016/j.psychres.2019.01.029.
327. Mason, T.B.; O'Connor, S.G.; Schembre, S.M.; Huh, J.; Chu, D.; Dunton, G.F. Momentary Affect, Stress Coping, and Food Intake in Mother-Child Dyads. *Health psychology : official journal of the Division of Health Psychology, American Psychological Association* **2019**, *38*, 238–247. https://doi.org/10.1037/hea0000714.
328. Zani, A.; Lobbezoo, F.; Bracci, A.; Ahlberg, J.; Manfredini, D. Ecological Momentary Assessment and Intervention Principles for the Study of Awake Bruxism Behaviors, Part 1: General Principles and Preliminary Data on Healthy Young Italian Adults. *Frontiers in Neurology* **2019**, *10*, 169. https://doi.org/10.3389/fneur.2019.00169.
329. Chan, Y.; So, S.H.w.; Mak, A.D.P.; Siah, K.T.H.; Chan, W.; Wu, J.C.Y. The temporal relationship of daily life stress, emotions, and bowel symptoms in irritable bowel syndrome—Diarrhea subtype: A smartphone-based experience sampling study. *Neurogastroenterology & Motility* **2019**, *31*, e13514. https://doi.org/10.1111/nmo.13514.
330. Sanjuan, P.M.; Pearson, M.R.; Poremba, C.; Amaro, H.; Leeman, L. An ecological momentary assessment study examining posttraumatic stress disorder symptoms, prenatal bonding, and substance use among pregnant women. *Drug and alcohol dependence* **2019**, *195*, 33–39. https://doi.org/10.1016/j.drugalcdep.2018.11.019.
331. Morita, P.P.; Yeung, M.S.; Ferrone, M.; Taite, A.K.; Madeley, C.; Stevens Lavigne, A.; To, T.; Lougheed, M.D.; Gupta, S.; Day, A.G.; et al. A Patient-Centered Mobile Health System That Supports Asthma Self-Management (breathe): Design, Development, and Utilization. *JMIR mHealth and uHealth* **2019**, *7*, e10956. https://doi.org/10.2196/10956.
332. Kreyenbuhl, J.; Record, E.J.; Himelhoch, S.; Charlotte, M.; Palmer-Bacon, J.; Dixon, L.B.; Medoff, D.R.; Li, L. Development and Feasibility Testing of a Smartphone Intervention to Improve Adherence to Antipsychotic Medications. *Clinical schizophrenia & related psychoses* **2019**, *12*, 152–167. https://doi.org/10.3371/CSRP.KRRE.070816.
333. Taylor, S.; Ferguson, C.; Peng, F.; Schoeneich, M.; Picard, R.W. Use of In-Game Rewards to Motivate Daily Self-Report Compliance: Randomized Controlled Trial. *Journal of Medical Internet Research* **2019**, *21*, e11683. https://doi.org/10.2196/11683.

334. Hyun, J.; Sliwinski, M.J.; Smyth, J.M. Waking Up on the Wrong Side of the Bed: The Effects of Stress Anticipation on Working Memory in Daily Life. *The Journals of Gerontology Series B: Psychological Sciences and Social Sciences* **2019**, *74*, 38–46. https://doi.org/10.1093/geronb/gby042.
335. Roberts, M.E.; Keller-Hamilton, B.; Hinton, A.; Browning, C.R.; Slater, M.D.; Xi, W.; Ferketich, A.K. The magnitude and impact of tobacco marketing exposure in adolescents' day-to-day lives: An ecological momentary assessment (EMA) study. *Addictive behaviors* **2019**, *88*, 144–149. https://doi.org/10.1016/j.addbeh.2018.08.035.
336. Matz-Costa, C.; Cosner Berzin, S.; Pitt-Catsouphes, M.; Halvorsen, C.J. Perceptions of the Meaningfulness of Work Among Older Social Purpose Workers: An Ecological Momentary Assessment Study. *Journal of Applied Gerontology* **2019**, *38*, 1121–1146. https://doi.org/10.1177/0733464817727109.
337. Victor, S.E.; Christensen, K.; Johnson, S.L.; Van Allen, J.; Brick, L.A. Dynamic Regulatory Processes in the Transition From Suicidal Ideation to Action in Adults Leaving Inpatient Psychiatric Care: Protocol for an Intensive Longitudinal Study. *JMIR Research Protocols* **2022**, *11*, e38582. https://doi.org/10.2196/38582.
338. Zhu, T.; Uduku, C.; Li, K.; Herrero, P.; Oliver, N.; Georgiou, P. Enhancing self-management in type 1 diabetes with wearables and deep learning. *npj Digital Medicine* **2022**, *5*, 1–11. https://doi.org/10.1038/s41746-022-00626-5.
339. Zink, J.; Yang, C.H.; Alves, J.M.; McAlister, K.L.; Huh, J.; Pentz, M.A.; Page, K.A.; Dunton, G.F.; Belcher, B.R. Time-Varying Associations Between Device-Based and Ecological Momentary Assessment–Reported Sedentary Behaviors and the Concurrent Affective States Among Adolescents: Proof-of-Concept Study. *JMIR Formative Research* **2022**, *6*, e37743. https://doi.org/10.2196/37743.
340. Mousavi, Z.A.; Lai, J.; Simon, K.; Rivera, A.P.; Yunusova, A.; Hu, S.; Labbaf, S.; Jafarlou, S.; Dutt, N.D.; Jain, R.C.; et al. Sleep Patterns and Affect Dynamics Among College Students During the COVID-19 Pandemic: Intensive Longitudinal Study. *JMIR Formative Research* **2022**, *6*, e33964. https://doi.org/10.2196/33964.
341. Goldstein, S.P.; Hoover, A.; Thomas, J.G. Combining passive eating monitoring and ecological momentary assessment to characterize dietary lapses from a lifestyle modification intervention. *Appetite* **2022**, *175*, 106090. https://doi.org/10.1016/j.appet.2022.106090.
342. Nabasny, A.; Rabinowitz, A.; Wright, B.; Wang, J.; Preminger, S.; Terhorst, L.; Juengst, S.B. Neurobehavioral Symptoms and Heart Rate Variability: Feasibility of Remote Collection Using Mobile Health Technology. *The Journal of Head Trauma Rehabilitation* **2022**, *37*, 178–188. https://doi.org/10.1097/HTR.0000000000000764.
343. Hart, A.; Reis, D.; Prestele, E.; Jacobson, N.C. Using Smartphone Sensor Paradata and Personalized Machine Learning Models to Infer Participants' Well-being: Ecological Momentary Assessment. *Journal of Medical Internet Research* **2022**, *24*, e34015. https://doi.org/10.2196/34015.
344. Clapp, J.D.; Madden, D.R.; Pakdaman, S. Drinking with Friends: Measuring the Two-week Ecology of Drinking Behaviors. *American Journal of Health Behavior* **2022**, *46*, 96–113. https://doi.org/10.5993/AJHB.46.2.1.
345. Shrestha, R.; Telkmann, K.; Schüz, B.; Koju, P.; Shrestha, R.; Karmacharya, B.; Bolte, G. Measuring Environmental Justice in Real Time: A Pilot Study Using Digital Participatory Method in the Global South, Nepal. *International Journal of Environmental Research and Public Health* **2022**, *19*, 4752. https://doi.org/10.3390/ijerph19084752.
346. Cook, P.; Jankowski, C.; Erlandson, K.M.; Reeder, B.; Starr, W.; Flynn Makic, M.B. Low- and High-Intensity Physical Activity Among People with HIV: Multilevel Modeling Analysis Using Sensor- and Survey-Based Predictors. *JMIR mHealth and uHealth* **2022**, *10*, e33938. https://doi.org/10.2196/33938.
347. Mair, J.L.; Hayes, L.D.; Campbell, A.K.; Buchan, D.S.; Easton, C.; Sculthorpe, N. A Personalized Smartphone-Delivered Just-in-time Adaptive Intervention (JitaBug) to Increase Physical Activity in Older Adults: Mixed Methods Feasibility Study. *JMIR Formative Research* **2022**, *6*, e34662. https://doi.org/10.2196/34662.
348. Krohn, H.; Guintivano, J.; Frische, R.; Steed, J.; Rackers, H.; Meltzer-Brody, S. App-Based Ecological Momentary Assessment to Enhance Clinical Care for Postpartum Depression: Pilot Acceptability Study. *JMIR Formative Research* **2022**, *6*, e28081. https://doi.org/10.2196/28081.
349. Haucke, M.; Heinz, A.; Liu, S.; Heinzel, S. The Impact of COVID-19 Lockdown on Daily Activities, Cognitions, and Stress in a Lonely and Distressed Population: Temporal Dynamic Network Analysis. *Journal of Medical Internet Research* **2022**, *24*, e32598. https://doi.org/10.2196/32598.
350. Lucassen, D.A.; Brouwer-Brolsma, E.M.; Slotegraaf, A.I.; Kok, E.; Feskens, E.J.M. DIetary ASSessment (DIASS) Study: Design of an Evaluation Study to Assess Validity, Usability and Perceived Burden of an Innovative Dietary Assessment Methodology. *Nutrients* **2022**, *14*, 1156. https://doi.org/10.3390/nu14061156.
351. Zhang, P.; Fonnesbeck, C.; Schmidt, D.C.; White, J.; Kleinberg, S.; Mulvaney, S.A. Using Momentary Assessment and Machine Learning to Identify Barriers to Self-management in Type 1 Diabetes: Observational Study. *JMIR mHealth and uHealth* **2022**, *10*, e21959. https://doi.org/10.2196/21959.
352. Bell, B.M.; Alam, R.; Mondol, A.S.; Ma, M.; Emi, I.A.; Preum, S.M.; de la Haye, K.; Stankovic, J.A.; Lach, J.; Spruijt-Metz, D. Validity and Feasibility of the Monitoring and Modeling Family Eating Dynamics System to Automatically Detect In-field Family Eating Behavior: Observational Study. *JMIR mHealth and uHealth* **2022**, *10*, e30211. https://doi.org/10.2196/30211.
353. Zhai, D.; van Stiphout, R.; Schiavone, G.; De Raedt, W.; Van Hoof, C. Characterizing and Modeling Smoking Behavior Using Automatic Smoking Event Detection and Mobile Surveys in Naturalistic Environments: Observational Study. *JMIR mHealth and uHealth* **2022**, *10*, e28159. https://doi.org/10.2196/28159.

354. Carlier, C.; Niemeijer, K.; Mestdagh, M.; Bauwens, M.; Vanbrabant, P.; Geurts, L.; van Waterschoot, T.; Kuppens, P. In Search of State and Trait Emotion Markers in Mobile-Sensed Language: Field Study. *JMIR Mental Health* **2022**, *9*, e31724. https://doi.org/10.2196/31724.
355. Ponnada, A.; Wang, S.; Chu, D.; Do, B.; Dunton, G.; Intille, S. Intensive Longitudinal Data Collection Using Microinteraction Ecological Momentary Assessment: Pilot and Preliminary Results. *JMIR Formative Research* **2022**, *6*, e32772. https://doi.org/10.2196/32772.
356. Pellegrini, A.M.; Huang, E.J.; Staples, P.C.; Hart, K.L.; Lorme, J.M.; Brown, H.E.; Perlis, R.H.; Onnela, J.P.J. Estimating longitudinal depressive symptoms from smartphone data in a transdiagnostic cohort. *Brain and Behavior* **2022**, *12*, e02077. https://doi.org/10.1002/brb3.2077.
357. Sun-Suslow, N.; Campbell, L.M.; Tang, B.; Fisher, A.C.; Lee, E.; Paolillo, E.W.; Heaton, A.; Moore, R.C. Use of digital health technologies to examine subjective and objective sleep with next-day cognition and daily indicators of health in persons with and without HIV. *Journal of Behavioral Medicine* **2022**, *45*, 62–75. https://doi.org/10.1007/s10865-021-00233-x.
358. Zlatar, Z.Z.; Campbell, L.M.; Tang, B.; Gabin, S.; Heaton, A.; Higgins, M.; Swendsen, J.; Moore, D.J.; Moore, R.C. Daily Level Association of Physical Activity and Performance on Ecological Momentary Cognitive Tests in Free-living Environments: A Mobile Health Observational Study. *JMIR mHealth and uHealth* **2022**, *10*, e33747. https://doi.org/10.2196/33747.
359. Gansner, M.; Nisenson, M.; Lin, V.; Pong, S.; Torous, J.; Carson, N. Problematic Internet Use Before and During the COVID-19 Pandemic in Youth in Outpatient Mental Health Treatment: App-Based Ecological Momentary Assessment Study. *JMIR Mental Health* **2022**, *9*, e33114. https://doi.org/10.2196/33114.
360. Massar, S.A.A.; Ng, A.S.C.; Soon, C.S.; Ong, J.L.; Chua, X.Y.; Chee, N.I.Y.N.; Lee, T.S.; Chee, M.W.L. Reopening after lockdown: the influence of working-from-home and digital device use on sleep, physical activity, and wellbeing following COVID-19 lockdown and reopening. *Sleep* **2021**, *45*, zsab250. https://doi.org/10.1093/sleep/zsab250.
361. Bai, Y.; Copeland, W.E.; Burns, R.; Nardone, H.; Devadanam, V.; Rettew, J.; Hudziak, J. Ecological Momentary Assessment of Physical Activity and Wellness Behaviors in College Students Throughout a School Year: Longitudinal Naturalistic Study. *JMIR Public Health and Surveillance* **2022**, *8*, e25375. https://doi.org/10.2196/25375.
362. Fiedler, J.; Seiferth, C.; Eckert, T.; Woll, A.; Wunsch, K. Sleep quality, valence, energetic arousal, and calmness as predictors of device-based measured physical activity during a three-week mHealth intervention. *German Journal of Exercise and Sport Research* **2022**, *52*, 237–247. https://doi.org/10.1007/s12662-022-00809-y.
363. Russell, M.A.; Turrisi, R.J.; Smyth, J.M. Transdermal sensor features correlate with ecological momentary assessment drinking reports and predict alcohol-related consequences in young adults' natural settings. *Alcoholism: Clinical and Experimental Research* **2022**, *46*, 100–113. https://doi.org/10.1111/acer.14739.
364. Su, L.; Zhou, S.; Kwan, M.P.; Chai, Y.; Zhang, X. The impact of immediate urban environments on people's momentary happiness. *Urban Studies* **2022**, *59*, 140–160. https://doi.org/10.1177/0042098020986499.
365. Aalbers, G.; vanden Abeele, M.M.P.; Hendrickson, A.T.; de Marez, L.; Keijsers, L. Caught in the moment: Are there person-specific associations between momentary procrastination and passively measured smartphone use? *Mobile Media & Communication* **2022**, *10*, 115–135. https://doi.org/10.1177/2050157921993896.
366. Schilling, R.; Cody, R.; Ludyga, S.; Brand, S.; Faude, O.; Pühse, U.; Gerber, M. Does dispositional self-control moderate the association between stress at work and physical activity after work? A real-life study with police officers. *German Journal of Exercise and Sport Research* **2022**, *52*, 290–299. https://doi.org/10.1007/s12662-022-00810-5.
367. Martin, E.C.; Verkaik, R.; Stultiens, J.J.A.; van de Berg, M.R.; Janssen, A.M.L.; Leue, C.; Delespaul, P.; Peeters, F.; Widdershoven, J.; Erdkamp, A.; et al. The DizzyQuest: relation between self-reported hearing loss, tinnitus and objective hearing thresholds in patients with Meniere's disease. *Journal of Neurology* **2022**, *269*, 5239–5248. https://doi.org/10.1007/s00415-021-10909-8.
368. Miller, S.; Teufel, R.; Nichols, M.; Davenport, P.; Mueller, M.; Silverman, E.; Madisetti, M.; Pittman, M.; Kelechi, T.; Strange, C. Feasibility of RESP-FIT: Technology-Enhanced Self-Management Intervention for Adults with COPD. *International Journal of Chronic Obstructive Pulmonary Disease* **2021**, *16*, 3263–3273. https://doi.org/10.2147/COPD.S326675.
369. Harrington, K.; Zenk, S.N.; Van Horn, L.; Giurini, L.; Mahakala, N.; Kershaw, K.N. The Use of Food Images and Crowdsourcing to Capture Real-time Eating Behaviors: Acceptability and Usability Study. *JMIR formative research* **2021**, *5*, e27512. https://doi.org/10.2196/27512.
370. O'Rourke, N.; Sixsmith, A.; BADAS Study Team. Ecological momentary assessment of mood and movement with bipolar disorder over time: Participant recruitment and efficacy of study methods. *International Journal of Methods in Psychiatric Research* **2021**, *30*, e1895. https://doi.org/10.1002/mpr.1895.
371. Hevel, D.J.; Drollette, E.S.; Dunton, G.F.; Maher, J.P. Social and Physical Context Moderates Older Adults' Affective Responses to Sedentary Behavior: An Ecological Momentary Assessment Study. *The Journals of Gerontology. Series B, Psychological Sciences and Social Sciences* **2021**, *76*, 1983–1992. https://doi.org/10.1093/geronb/gbab036.
372. Elavsky, S.; Klocek, A.; Knapova, L.; Smahelova, M.; Smahel, D.; Cimler, R.; Kuhnova, J. Feasibility of Real-time Behavior Monitoring Via Mobile Technology in Czech Adults Aged 50 Years and Above: 12-Week Study With Ecological Momentary Assessment. *JMIR aging* **2021**, *4*, e15220. https://doi.org/10.2196/15220.
373. Labhart, F.; Phan, T.T.; Gatica-Perez, D.; Kuntsche, E. Shooting shots: Estimating alcoholic drink sizes in real life using event-level reports and annotations of close-up pictures. *Drug and Alcohol Review* **2021**, *40*, 1228–1238. https://doi.org/10.1111/dar.13212.

374. Jenstad, L.M.; Singh, G.; Boretzki, M.; DeLongis, A.; Fichtl, E.; Ho, R.; Huen, M.; Meyer, V.; Pang, F.; Stephenson, E. Ecological Momentary Assessment: A Field Evaluation of Subjective Ratings of Speech in Noise. *Ear and Hearing* **2021**, *42*, 1770–1781. https://doi.org/10.1097/AUD.0000000000001071.
375. Kim, J.; Murata, T.; Foo, J.C.; Md Azmol Hossain, B.; Togo, F. A Pilot Study of Temporal Associations Between Psychological Stress and Cardiovascular Response. In Proceedings of the 2021 43rd Annual International Conference of the IEEE Engineering in Medicine & Biology Society (EMBC), 2021, pp. 7040–7043. https://doi.org/10.1109/EMBC46164.2021.9630872.
376. Engelhard, M.M.; D'Arcy, J.; Oliver, J.A.; Kozink, R.; McClernon, F.J. Prediction of Smoking Risk From Repeated Sampling of Environmental Images: Model Validation. *Journal of Medical Internet Research* **2021**, *23*, e27875. https://doi.org/10.2196/27875.
377. Norman, T.; Peacock, A.; Ferguson, S.G.; Kuntsche, E.; Bruno, R. Combining transdermal and breath alcohol assessments, real-time drink logs and retrospective self-reports to measure alcohol consumption and intoxication across a multi-day music festival. *Drug and Alcohol Review* **2021**, *40*, 1112–1121. https://doi.org/10.1111/dar.13215.
378. Schumacher, L.M.; Espel-Huynh, H.M.; Thomas, J.G.; Vithiananthan, S.; Jones, D.B.; Bond, D.S. Energy, Attentiveness, and Fatigue After Bariatric Surgery and Associations with Daily Physical Activity and Weight Loss: an Ecological Momentary Assessment Study. *Obesity Surgery* **2021**, *31*, 4893–4900. https://doi.org/10.1007/s11695-021-05662-w.
379. Camenga, D.R.; Haeny, A.M.; Krishnan-Sarin, S.; O'Malley, S.S.; Bold, K.W. Pilot Ecological Momentary Assessment Study of Subjective and Contextual Factors Surrounding E-Cigarette and Combustible Tobacco Product Use among Young Adults. *International Journal of Environmental Research and Public Health* **2021**, *18*. https://doi.org/10.3390/ijerph182111005.
380. Khanshan, A.; Van Gorp, P.; Nuijten, R.; Markopoulos, P. Assessing the Influence of Physical Activity Upon the Experience Sampling Response Rate on Wrist-Worn Devices. *International Journal of Environmental Research and Public Health* **2021**, *18*, 10593. https://doi.org/10.3390/ijerph182010593.
381. de Vries, H.; Kamphuis, W.; Oldenhuis, H.; van der Schans, C.; Sanderman, R. Moderation of the Stressor-Strain Process in Interns by Heart Rate Variability Measured With a Wearable and Smartphone App: Within-Subject Design Using Continuous Monitoring. *JMIR cardio* **2021**, *5*, e28731. https://doi.org/10.2196/28731.
382. Giurgiu, M.; Nissen, R.; Müller, G.; Ebner-Priemer, U.W.; Reichert, M.; Clark, B. Drivers of productivity: Being physically active increases yet sedentary bouts and lack of sleep decrease work ability. *Scandinavian Journal of Medicine & Science in Sports* **2021**, *31*, 1921–1931. https://doi.org/10.1111/sms.14005.
383. Meyerhoff, J.; Liu, T.; Kording, K.P.; Ungar, L.H.; Kaiser, S.M.; Karr, C.J.; Mohr, D.C. Evaluation of Changes in Depression, Anxiety, and Social Anxiety Using Smartphone Sensor Features: Longitudinal Cohort Study. *Journal of Medical Internet Research* **2021**, *23*, e22844. https://doi.org/10.2196/22844.
384. Thøgersen-Ntoumani, C.; Dodos, L.A.; Stenling, A.; Ntoumanis, N. Does self-compassion help to deal with dietary lapses among overweight and obese adults who pursue weight-loss goals? *British Journal of Health Psychology* **2021**, *26*, 767–788. https://doi.org/10.1111/bjhp.12499.
385. Kamalyan, L.; Yang, J.A.; Pope, C.N.; Paolillo, E.W.; Campbell, L.M.; Tang, B.; Marquine, M.J.; Depp, C.A.; Moore, R.C. Increased Social Interactions Reduce the Association Between Constricted Life-Space and Lower Daily Happiness in Older Adults With and Without HIV: A GPS and Ecological Momentary Assessment Study. *The American Journal of Geriatric Psychiatry: Official Journal of the American Association for Geriatric Psychiatry* **2021**, *29*, 867–879. https://doi.org/10.1016/j.jagp.2020.11.005.
386. Pinto, B.M.; Kindred, M.D.; Dunsiger, S.I.; Williams, D.M. Sedentary behavior among breast cancer survivors: a longitudinal study using ecological momentary assessments. *Journal of Cancer Survivorship* **2021**, *15*, 546–553. https://doi.org/10.1007/s11764-020-00948-x.
387. Alinia, P.; Sah, R.K.; McDonell, M.; Pendry, P.; Parent, S.; Ghasemzadeh, H.; Cleveland, M.J. Associations Between Physiological Signals Captured Using Wearable Sensors and Self-reported Outcomes Among Adults in Alcohol Use Disorder Recovery: Development and Usability Study. *JMIR formative research* **2021**, *5*, e27891. https://doi.org/10.2196/27891.
388. Beierle, F.; Schobel, J.; Vogel, C.; Allgaier, J.; Mulansky, L.; Haug, F.; Haug, J.; Schlee, W.; Holfelder, M.; Stach, M.; et al. Corona Health-A Study- and Sensor-Based Mobile App Platform Exploring Aspects of the COVID-19 Pandemic. *International Journal of Environmental Research and Public Health* **2021**, *18*, 7395. https://doi.org/10.3390/ijerph18147395.
389. Do, B.; Mason, T.B.; Yi, L.; Yang, C.H.; Dunton, G.F. Momentary associations between stress and physical activity among children using ecological momentary assessment. *Psychology of sport and exercise* **2021**, *55*, 101935. https://doi.org/10.1016/j.psychsport.2021.101935.
390. Shui, X.; Zhang, M.; Li, Z.; Hu, X.; Wang, F.; Zhang, D. A dataset of daily ambulatory psychological and physiological recording for emotion research. *Scientific Data* **2021**, *8*, 161. https://doi.org/10.1038/s41597-021-00945-4.
391. Wu, C.; Fritz, H.; Bastami, S.; Maestre, J.P.; Thomaz, E.; Julien, C.; Castelli, D.M.; de Barbaro, K.; Bearman, S.K.; Harari, G.M.; et al. Multi-modal data collection for measuring health, behavior, and living environment of large-scale participant cohorts. *GigaScience* **2021**, *10*, giab044. https://doi.org/10.1093/gigascience/giab044.
392. Nam, S.; Jeon, S.; Ash, G.; Whittemore, R.; Vlahov, D. Racial Discrimination, Sedentary Time, and Physical Activity in African Americans: Quantitative Study Combining Ecological Momentary Assessment and Accelerometers. *JMIR formative research* **2021**, *5*, e25687. https://doi.org/10.2196/25687.
393. Massar, S.A.A.; Chua, X.Y.; Soon, C.S.; Ng, A.S.C.; Ong, J.L.; Chee, N.I.Y.N.; Lee, T.S.; Ghosh, A.; Chee, M.W.L. Trait-like nocturnal sleep behavior identified by combining wearable, phone-use, and self-report data. *NPJ digital medicine* **2021**, *4*, 90. https://doi.org/10.1038/s41746-021-00466-9.

394. Buck, B.; Hallgren, K.A.; Campbell, A.T.; Choudhury, T.; Kane, J.M.; Ben-Zeev, D. mHealth-Assisted Detection of Precursors to Relapse in Schizophrenia. *Frontiers in Psychiatry* **2021**, *12*, 642200. https://doi.org/10.3389/fpsyt.2021.642200.
395. Li, Z.; Benowitz-Fredericks, C.; Ling, P.M.; Cohen, J.E.; Thrul, J. Assessing Young Adults' ENDS Use via Ecological Momentary Assessment and a Smart Bluetooth Enabled ENDS Device. *Nicotine & Tobacco Research* **2020**, *23*, 842–848. https://doi.org/10.1093/ntr/ntaa205.
396. Naya, C.H.; Zink, J.; Huh, J.; Dunton, G.F.; Belcher, B.R. Examining the same-day relationship between morning cortisol after awakening, perceived stress in the morning, and physical activity in youth. *Stress (Amsterdam, Netherlands)* **2021**, *24*, 338–347. https://doi.org/10.1080/10253890.2020.1804852.
397. Stewart, M.T.; Nezich, T.; Lee, J.M.; Hasson, R.E.; Colabianchi, N. Using a Mobile Phone App to Analyze the Relationship Between Planned and Performed Physical Activity in University Students: Observational Study. *JMIR mHealth and uHealth* **2021**, *9*, e17581. https://doi.org/10.2196/17581.
398. Kracht, C.L.; Beyl, R.A.; Maher, J.P.; Katzmarzyk, P.T.; Staiano, A.E. Adolescents' sedentary time, affect, and contextual factors: An ecological momentary assessment study. *International Journal of Behavioral Nutrition and Physical Activity* **2021**, *18*, 53. https://doi.org/10.1186/s12966-021-01121-y.
399. Mun, E.Y.; Li, X.; Businelle, M.S.; Hébert, E.T.; Tan, Z.; Barnett, N.P.; Walters, S.T. Ecological Momentary Assessment of Alcohol Consumption and Its Concordance with Transdermal Alcohol Detection and Timeline Follow-Back Self-report Among Adults Experiencing Homelessness. *Alcoholism: Clinical and Experimental Research* **2021**, *45*, 864–876. https://doi.org/10.1111/acer.14571.
400. Difrancesco, S.; Penninx, B.W.J.H.; Antypa, N.; van Hemert, A.M.; Riese, H.; Lamers, F. The day-to-day bidirectional longitudinal association between objective and self-reported sleep and affect: An ambulatory assessment study. *Journal of Affective Disorders* **2021**, *283*, 165–171. https://doi.org/10.1016/j.jad.2021.01.052.
401. Ponnada, A.; Thapa-Chhetry, B.; Manjourides, J.; Intille, S. Measuring Criterion Validity of Microinteraction Ecological Momentary Assessment (Micro-EMA): Exploratory Pilot Study With Physical Activity Measurement. *JMIR mHealth and uHealth* **2021**, *9*, e23391. https://doi.org/10.2196/23391.
402. Connelly, M.A.; Boorigie, M.E. Feasibility of using "SMARTER" methodology for monitoring precipitating conditions of pediatric migraine episodes. *Headache: The Journal of Head and Face Pain* **2021**, *61*, 500–510. https://doi.org/10.1111/head.14028.
403. König, L.M.; Koller, J.E.; Villinger, K.; Wahl, D.R.; Ziesemer, K.; Schupp, H.T.; Renner, B. Investigating the Relationship between Perceived Meal Colour Variety and Food Intake across Meal Types in a Smartphone-Based Ecological Momentary Assessment. *Nutrients* **2021**, *13*, 755. https://doi.org/10.3390/nu13030755.
404. Hevel, D.J.; Dunton, G.F.; Maher, J.P. Acute Bidirectional Relations Between Affect, Physical Feeling States, and Activity-Related Behaviors Among Older Adults: An Ecological Momentary Assessment Study. *Annals of Behavioral Medicine* **2021**, *55*, 41–54. https://doi.org/10.1093/abm/kaaa027.
405. Allicock, M.; Kendzor, D.; Sedory, A.; Gabriel, K.P.; Swartz, M.D.; Thomas, P.; Yudkin, J.S.; Rivers, A. A Pilot and Feasibility Mobile Health Intervention to Support Healthy Behaviors in African American Breast Cancer Survivors. *Journal of Racial and Ethnic Health Disparities* **2021**, *8*, 157–165. https://doi.org/10.1007/s40615-020-00767-x.
406. Wen, H.; Sobolev, M.; Vitale, R.; Kizer, J.; Pollak, J.P.; Muench, F.; Estrin, D. mPulse Mobile Sensing Model for Passive Detection of Impulsive Behavior: Exploratory Prediction Study. *JMIR mental health* **2021**, *8*, e25019. https://doi.org/10.2196/25019.
407. von Gablenz, P.; Kowalk, U.; Bitzer, J.; Meis, M.; Holube, I. Individual Hearing Aid Benefit in Real Life Evaluated Using Ecological Momentary Assessment. *Trends in Hearing* **2021**, *25*, 2331216521990288. https://doi.org/10.1177/2331216521990288.
408. Ghosh, S.; Ganguly, N.; Mitra, B.; De, P. Designing an Experience Sampling Method for Smartphone Based Emotion Detection. *IEEE Transactions on Affective Computing* **2021**, *12*, 913–927. https://doi.org/10.1109/TAFFC.2019.2905561.
409. Ghosh, S.; Mandi, S.; Mitra, B.; De, P. Exploring Smartphone Keyboard Interactions for Experience Sampling Method driven Probe Generation. In Proceedings of the 26th International Conference on Intelligent User Interfaces; Association for Computing Machinery: New York, NY, USA, 2021; IUI '21, pp. 133–138. https://doi.org/10.1145/3397481.3450669.
410. Wu, Y.H.; Xu, J.; Stangl, E.; Pentony, S.; Vyas, D.; Chipara, O.; Gudjonsdottir, A.; Oleson, J.; Galster, J. Why Ecological Momentary Assessment Surveys Go Incomplete: When It Happens and How It Impacts Data. *Journal of the American Academy of Audiology* **2021**, *32*, 16–26. https://doi.org/10.1055/s-0040-1719135.
411. Tseng, V.W.; Costa, J.D.R.; Jung, M.F.; Choudhury, T. Using Smartphone Sensor Data to Assess Inhibitory Control in the Wild: Longitudinal Study. *JMIR mHealth and uHealth* **2020**, *8*, e21703. https://doi.org/10.2196/21703.
412. Raugh, I.M.; James, S.H.; Gonzalez, C.M.; Chapman, H.C.; Cohen, A.S.; Kirkpatrick, B.; Strauss, G.P. Geolocation as a Digital Phenotyping Measure of Negative Symptoms and Functional Outcome. *Schizophrenia Bulletin* **2020**, *46*, 1596–1607. https://doi.org/10.1093/schbul/sbaa121.
413. Nakajima, M.; Lemieux, A.M.; Fiecas, M.; Chatterjee, S.; Sarker, H.; Saleheen, N.; Ertin, E.; Kumar, S.; al'Absi, M. Using novel mobile sensors to assess stress and smoking lapse. *International journal of psychophysiology : official journal of the International Organization of Psychophysiology* **2020**, *158*, 411–418. https://doi.org/10.1016/j.ijpsycho.2020.11.005.
414. Munasinghe, S.; Sperandei, S.; Freebairn, L.; Conroy, E.; Jani, H.; Marjanovic, S.; Page, A. The Impact of Physical Distancing Policies During the COVID-19 Pandemic on Health and Well-Being Among Australian Adolescents. *The Journal of Adolescent Health: Official Publication of the Society for Adolescent Medicine* **2020**, *67*, 653–661. https://doi.org/10.1016/j.jadohealth.2020.08.008.

415. Das-Friebel, A.; Lenneis, A.; Realo, A.; Sanborn, A.; Tang, N.K.Y.; Wolke, D.; von Mühlenen, A.; Lemola, S. Bedtime social media use, sleep, and affective wellbeing in young adults: an experience sampling study. *Journal of Child Psychology and Psychiatry* **2020**, *61*, 1138–1149. https://doi.org/10.1111/jcpp.13326.
416. Giurgiu, M.; Niermann, C.; Ebner-Priemer, U.; Kanning, M. Accuracy of Sedentary Behavior-Triggered Ecological Momentary Assessment for Collecting Contextual Information: Development and Feasibility Study. *JMIR mHealth and uHealth* **2020**, *8*, e17852. https://doi.org/10.2196/17852.
417. Mendez, D.D.; Sanders, S.A.; Lai, Y.H.; Wallace, M.L.; Rathbun, S.L.; Gary-Webb, T.L.; Davis, E.M.; Burke, L.E. Ecological momentary assessment of stress, racism and other forms of discrimination during pregnancy using smartphone technology. *Paediatric and Perinatal Epidemiology* **2020**, *34*, 522–531. https://doi.org/10.1111/ppe.12619.
418. Di Matteo, D.; Fotinos, K.; Lokuge, S.; Yu, J.; Sternat, T.; Katzman, M.A.; Rose, J. The Relationship Between Smartphone-Recorded Environmental Audio and Symptomatology of Anxiety and Depression: Exploratory Study. *JMIR formative research* **2020**, *4*, e18751. https://doi.org/10.2196/18751.
419. Elliston, K.G.; Schüz, B.; Albion, T.; Ferguson, S.G. Comparison of Geographic Information System and Subjective Assessments of Momentary Food Environments as Predictors of Food Intake: An Ecological Momentary Assessment Study. *JMIR mHealth and uHealth* **2020**, *8*, e15948. https://doi.org/10.2196/15948.
420. Campbell, L.M.; Tang, B.; Watson, C.W.M.; Higgins, M.; Cherner, M.; Henry, B.L.; Moore, R.C. Cannabis use is associated with greater total sleep time in middle-aged and older adults with and without HIV: A preliminary report utilizing digital health technologies. *Cannabis (Research Society on Marijuana)* **2020**, *3*, 180–189. https://doi.org/10.26828/cannabis.2020.02.005.
421. Zhang, X.; Zhou, S.; Kwan, M.P.; Su, L.; Lu, J. Geographic Ecological Momentary Assessment (GEMA) of environmental noise annoyance: the influence of activity context and the daily acoustic environment. *International Journal of Health Geographics* **2020**, *19*, 50. https://doi.org/10.1186/s12942-020-00246-w.
422. Jacobson, N.C.; Chung, Y.J. Passive Sensing of Prediction of Moment-To-Moment Depressed Mood among Undergraduates with Clinical Levels of Depression Sample Using Smartphones. *Sensors (Basel, Switzerland)* **2020**, *20*, E3572. https://doi.org/10.3390/s20123572.
423. Sengupta, A.; Beckie, T.; Dutta, K.; Dey, A.; Chellappan, S. A Mobile Health Intervention System for Women With Coronary Heart Disease: Usability Study. *JMIR formative research* **2020**, *4*, e16420. https://doi.org/10.2196/16420.
424. Huckins, J.F.; daSilva, A.W.; Wang, W.; Hedlund, E.; Rogers, C.; Nepal, S.K.; Wu, J.; Obuchi, M.; Murphy, E.I.; Meyer, M.L.; et al. Mental Health and Behavior of College Students During the Early Phases of the COVID-19 Pandemic: Longitudinal Smartphone and Ecological Momentary Assessment Study. *Journal of Medical Internet Research* **2020**, *22*, e20185. https://doi.org/10.2196/20185.
425. Norman, ; Kjellenberg, K.; Torres Aréchiga, D.; Löf, M.; Patterson, E. "Everyone can take photos." Feasibility and relative validity of phone photography-based assessment of children's diets – a mixed methods study. *Nutrition Journal* **2020**, *19*, 50. https://doi.org/10.1186/s12937-020-00558-4.
426. de Brito, J.N.; Loth, K.A.; Tate, A.; Berge, J.M. Associations Between Parent Self-Reported and Accelerometer-Measured Physical Activity and Sedentary Time in Children: Ecological Momentary Assessment Study. *JMIR mHealth and uHealth* **2020**, *8*, e15458. https://doi.org/10.2196/15458.
427. Habets, J.; Heijmans, M.; Herff, C.; Simons, C.; Leentjens, A.F.; Temel, Y.; Kuijf, M.; Kubben, P. Mobile Health Daily Life Monitoring for Parkinson Disease: Development and Validation of Ecological Momentary Assessments. *JMIR mHealth and uHealth* **2020**, *8*, e15628. https://doi.org/10.2196/15628.
428. Yang, C.H.; Huh, J.; Mason, T.B.; Belcher, B.R.; Kanning, M.; Dunton, G.F. Mother-child dyadic influences of affect on everyday movement behaviors: evidence from an ecological momentary assessment study. *The International Journal of Behavioral Nutrition and Physical Activity* **2020**, *17*, 56. https://doi.org/10.1186/s12966-020-00951-6.
429. Narziev, N.; Goh, H.; Toshnazarov, K.; Lee, S.A.; Chung, K.M.; Noh, Y. STDD: Short-Term Depression Detection with Passive Sensing. *Sensors* **2020**, *20*, 1396. https://doi.org/10.3390/s20051396.
430. Rijsbergen, M.; Niemeyer-van der Kolk, T.; Rijneveld, R.; Pinckaers, J.H.F.M.; Meshcheriakov, I.; Bouwes Bavinck, J.N.; van Doorn, M.B.A.; Hogendoorn, G.; Feiss, G.; Cohen, A.F.; et al. Mobile e-diary application facilitates the monitoring of patient-reported outcomes and a high treatment adherence for clinical trials in dermatology. *Journal of the European Academy of Dermatology and Venereology: JEADV* **2020**, *34*, 633–639. https://doi.org/10.1111/jdv.15872.
431. Poelman, M.P.; van Lenthe, F.J.; Scheider, S.; Kamphuis, C.B. A Smartphone App Combining Global Positioning System Data and Ecological Momentary Assessment to Track Individual Food Environment Exposure, Food Purchases, and Food Consumption: Protocol for the Observational FoodTrack Study. *JMIR research protocols* **2020**, *9*, e15283. https://doi.org/10.2196/15283.
432. Kondo, M.C.; Triguero-Mas, M.; Donaire-Gonzalez, D.; Seto, E.; Valentín, A.; Hurst, G.; Carrasco-Turigas, G.; Masterson, D.; Ambròs, A.; Ellis, N.; et al. Momentary mood response to natural outdoor environments in four European cities. *Environment International* **2020**, *134*, 105237. https://doi.org/10.1016/j.envint.2019.105237.
433. Schilling, R.; Herrmann, C.; Ludyga, S.; Colledge, F.; Brand, S.; Pühse, U.; Gerber, M. Does Cardiorespiratory Fitness Buffer Stress Reactivity and Stress Recovery in Police Officers? A Real-Life Study. *Frontiers in Psychiatry* **2020**, *11*, 594. https://doi.org/10.3389/fpsyt.2020.00594.
434. Ben-Zeev, D.; Buck, B.; Chander, A.; Brian, R.; Wang, W.; Atkins, D.; Brenner, C.J.; Cohen, T.; Campbell, A.; Munson, J. Mobile RDoC: Using Smartphones to Understand the Relationship Between Auditory Verbal Hallucinations and Need for Care. *Schizophrenia Bulletin Open* **2020**, *1*, sgaa060. https://doi.org/10.1093/schizbullopen/sgaa060.

435. Rabbi, M.; Li, K.; Yan, H.Y.; Hall, K.; Klasnja, P.; Murphy, S. ReVibe: A Context-assisted Evening Recall Approach to Improve Self-report Adherence. *Proceedings of the ACM on interactive, mobile, wearable and ubiquitous technologies* **2019**, *3*, 1–27. https://doi.org/10.1145/3369806.
436. Fairbairn, C.E.; Rosen, I.G.; Luczak, S.E.; Venerable, W.J. Estimating the quantity and time course of alcohol consumption from transdermal alcohol sensor data: A combined laboratory-ambulatory study. *Alcohol (Fayetteville, N.Y.)* **2019**, *81*, 111–116. https://doi.org/10.1016/j.alcohol.2018.08.015.
437. Depp, C.A.; Bashem, J.; Moore, R.C.; Holden, J.L.; Mikhael, T.; Swendsen, J.; Harvey, P.D.; Granholm, E.L. GPS mobility as a digital biomarker of negative symptoms in schizophrenia: a case control study. *npj Digital Medicine* **2019**, *2*, 1–7. https://doi.org/10.1038/s41746-019-0182-1.
438. Tomko, R.L.; McClure, E.A.; Cato, P.A.; Wang, J.B.; Carpenter, M.J.; Karelitz, J.L.; Froeliger, B.; Saladin, M.E.; Gray, K.M. An electronic, smart lighter to measure cigarette smoking: A pilot study to assess feasibility and initial validity. *Addictive Behaviors* **2019**, *98*, 106052. https://doi.org/10.1016/j.addbeh.2019.106052.
439. Schuwerk, T.; Kaltefleiter, L.J.; Au, J.Q.; Hoesl, A.; Stachl, C. Enter the Wild: Autistic Traits and Their Relationship to Mentalizing and Social Interaction in Everyday Life. *Journal of Autism and Developmental Disorders* **2019**, *49*, 4193–4208. https://doi.org/10.1007/s10803-019-04134-6.
440. Heijmans, M.; Habets, J.G.V.; Herff, C.; Aarts, J.; Stevens, A.; Kuijf, M.L.; Kubben, P.L. Monitoring Parkinson's disease symptoms during daily life: a feasibility study. *NPJ Parkinson's disease* **2019**, *5*, 21. https://doi.org/10.1038/s41531-019-0093-5.
441. Giurgiu, M.; Koch, E.D.; Ottenbacher, J.; Plotnikoff, R.C.; Ebner-Priemer, U.W.; Reichert, M. Sedentary behavior in everyday life relates negatively to mood: An ambulatory assessment study. *Scandinavian Journal of Medicine & Science in Sports* **2019**, *29*, 1340–1351. https://doi.org/10.1111/sms.13448.
442. Wray, T.B.; Pérez, A.E.; Celio, M.A.; Carr, D.J.; Adia, A.C.; Monti, P.M. Exploring the Use of Smartphone Geofencing to Study Characteristics of Alcohol Drinking Locations in High-Risk Gay and Bisexual Men. *Alcoholism, Clinical and Experimental Research* **2019**, *43*, 900–906. https://doi.org/10.1111/acer.13991.
443. Romanzini, C.L.P.; Romanzini, M.; Barbosa, C.C.L.; Batista, M.B.; Shigaki, G.B.; Ronque, E.R.V. Characterization and Agreement Between Application of Mobile Ecological Momentary Assessment (mEMA) and Accelerometry in the Identification of Prevalence of Sedentary Behavior (SB) in Young Adults. *Frontiers in Psychology* **2019**, *10*, 720. https://doi.org/10.3389/fpsyg.2019.00720.
444. Srinivas, P.; Bodke, K.; Ofner, S.; Keith, N.R.; Tu, W.; Clark, D.O. Context-Sensitive Ecological Momentary Assessment: Application of User-Centered Design for Improving User Satisfaction and Engagement During Self-Report. *JMIR mHealth and uHealth* **2019**, *7*, e10894. https://doi.org/10.2196/10894.
445. Goodale, B.M.; Shilaih, M.; Falco, L.; Dammeier, F.; Hamvas, G.; Leeners, B. Wearable Sensors Reveal Menses-Driven Changes in Physiology and Enable Prediction of the Fertile Window: Observational Study. *Journal of Medical Internet Research* **2019**, *21*, e13404. https://doi.org/10.2196/13404.
446. Rozet, A.; Kronish, I.M.; Schwartz, J.E.; Davidson, K.W. Using Machine Learning to Derive Just-In-Time and Personalized Predictors of Stress: Observational Study Bridging the Gap Between Nomothetic and Ideographic Approaches. *Journal of Medical Internet Research* **2019**, *21*, e12910. https://doi.org/10.2196/12910.
447. Manini, T.M.; Mendoza, T.; Battula, M.; Davoudi, A.; Kheirkhahan, M.; Young, M.E.; Weber, E.; Fillingim, R.B.; Rashidi, P. Perception of Older Adults Toward Smartwatch Technology for Assessing Pain and Related Patient-Reported Outcomes: Pilot Study. *JMIR mHealth and uHealth* **2019**, *7*, e10044. https://doi.org/10.2196/10044.
448. Tasian, G.E.; Ross, M.; Song, L.; Audrain-McGovern, J.; Wiebe, D.; Warner, S.G.; Henderson, B.; Patel, A.; Furth, S.L. Ecological Momentary Assessment of Factors Associated with Water Intake among Adolescents with Kidney Stone Disease. *The Journal of Urology* **2019**, *201*, 606–614. https://doi.org/10.1016/j.juro.2018.07.064.
449. Merikangas, K.R.; Swendsen, J.; Hickie, I.B.; Cui, L.; Shou, H.; Merikangas, A.K.; Zhang, J.; Lamers, F.; Crainiceanu, C.; Volkow, N.D.; et al. Real-time Mobile Monitoring of the Dynamic Associations Among Motor Activity, Energy, Mood, and Sleep in Adults With Bipolar Disorder. *JAMA psychiatry* **2019**, *76*, 190–198. https://doi.org/10.1001/jamapsychiatry.2018.3546.
450. Teufel Ii, R.J.; Patel, S.K.; Shuler, A.B.; Andrews, A.L.; Nichols, M.; Ebeling, M.D.; Dawley, E.; Mueller, M.; Ruggiero, K.J.; Treiber, F.A. Smartphones for Real-time Assessment of Adherence Behavior and Symptom Exacerbation for High-Risk Youth with Asthma: Pilot Study. *JMIR pediatrics and parenting* **2018**, *1*, e8. https://doi.org/10.2196/pediatrics.9796.
451. Maher, J.P.; Rebar, A.L.; Dunton, G.F. Ecological Momentary Assessment Is a Feasible and Valid Methodological Tool to Measure Older Adults' Physical Activity and Sedentary Behavior. *Frontiers in Psychology* **2018**, *9*, 1485. https://doi.org/10.3389/fpsyg.2018.01485.
452. Zulueta, J.; Piscitello, A.; Rasic, M.; Easter, R.; Babu, P.; Langenecker, S.A.; McInnis, M.; Ajilore, O.; Nelson, P.C.; Ryan, K.; et al. Predicting Mood Disturbance Severity with Mobile Phone Keystroke Metadata: A BiAffect Digital Phenotyping Study. *Journal of Medical Internet Research* **2018**, *20*, e241. https://doi.org/10.2196/jmir.9775.
453. Mulvaney, S.A.; Vaala, S.; Hood, K.K.; Lybarger, C.; Carroll, R.; Williams, L.; Schmidt, D.C.; Johnson, K.; Dietrich, M.S.; Laffel, L. Mobile Momentary Assessment and Biobehavioral Feedback for Adolescents with Type 1 Diabetes: Feasibility and Engagement Patterns. *Diabetes Technology & Therapeutics* **2018**, *20*, 465–474. https://doi.org/10.1089/dia.2018.0064.
454. Zink, J.; Belcher, B.R.; Dzubur, E.; Ke, W.; O'Connor, S.; Huh, J.; Lopez, N.; Maher, J.P.; Dunton, G.F. Association Between Self-Reported and Objective Activity Levels by Demographic Factors: Ecological Momentary Assessment Study in Children. *JMIR mHealth and uHealth* **2018**, *6*, e150. https://doi.org/10.2196/mhealth.9592.

455. Sano, A.; Taylor, S.; McHill, A.W.; Phillips, A.J.; Barger, L.K.; Klerman, E.; Picard, R. Identifying Objective Physiological Markers and Modifiable Behaviors for Self-Reported Stress and Mental Health Status Using Wearable Sensors and Mobile Phones: Observational Study. *Journal of Medical Internet Research* **2018**, *20*, e210. https://doi.org/10.2196/jmir.9410.
456. Kalmbach, D.A.; Fang, Y.; Arnedt, J.T.; Cochran, A.L.; Deldin, P.J.; Kaplin, A.I.; Sen, S. Effects of Sleep, Physical Activity, and Shift Work on Daily Mood: a Prospective Mobile Monitoring Study of Medical Interns. *Journal of General Internal Medicine* **2018**, *33*, 914–920. https://doi.org/10.1007/s11606-018-4373-2.
457. Cook, P.F.; Schmiege, S.J.; Bradley-Springer, L.; Starr, W.; Carrington, J.M. Motivation as a Mechanism for Daily Experiences' Effects on HIV Medication Adherence. *The Journal of the Association of Nurses in AIDS Care: JANAC* **2018**, *29*, 383–393. https://doi.org/10.1016/j.jana.2017.09.003.
458. Fairbairn, C.E.; Bresin, K.; Kang, D.; Rosen, I.G.; Ariss, T.; Luczak, S.E.; Barnett, N.P.; Eckland, N.S. A multimodal investigation of contextual effects on alcohol's emotional rewards. *Journal of Abnormal Psychology* **2018**, *127*, 359–373. https://doi.org/10.1037/abn0000346.
459. Minami, H.; Brinkman, H.R.; Nahvi, S.; Arnsten, J.H.; Rivera-Mindt, M.; Wetter, D.W.; Bloom, E.L.; Price, L.H.; Vieira, C.; Donnelly, R.; et al. Rationale, design and pilot feasibility results of a smartphone-assisted, mindfulness-based intervention for smokers with mood disorders: Project mSMART MIND. *Contemporary Clinical Trials* **2018**, *66*, 36–44. https://doi.org/10.1016/j.cct.2017.12.014.
460. Comulada, W.S.; Swendeman, D.; Koussa, M.K.; Mindry, D.; Medich, M.; Estrin, D.; Mercer, N.; Ramanathan, N. Adherence to self-monitoring healthy lifestyle behaviours through mobile phone-based ecological momentary assessments and photographic food records over 6 months in mostly ethnic minority mothers. *Public Health Nutrition* **2018**, *21*, 679–688. https://doi.org/10.1017/S1368980017003044.
461. Goldstein, S.P.; Thomas, J.G.; Vithiananthan, S.; Blackburn, G.A.; Jones, D.B.; Webster, J.; Jones, R.; Evans, E.W.; Dushay, J.; Moon, J.; et al. Multi-sensor ecological momentary assessment of behavioral and psychosocial predictors of weight loss following bariatric surgery: study protocol for a multicenter prospective longitudinal evaluation. *BMC obesity* **2018**, *5*, 27. https://doi.org/10.1186/s40608-018-0204-6.
462. Zhang, Y.; Qin, F.; Liu, B.; Qi, X.; Zhao, Y.; Zhang, D. Wearable Neurophysiological Recordings in Middle-School Classroom Correlate With Students' Academic Performance. *Frontiers in Human Neuroscience* **2018**, *12*, 457. https://doi.org/10.3389/fnhum.2018.00457.
463. Vigo, M.; Hassan, L.; Vance, W.; Jay, C.; Brass, A.; Cruickshank, S. Britain Breathing: using the experience sampling method to collect the seasonal allergy symptoms of a country. *Journal of the American Medical Informatics Association: JAMIA* **2018**, *25*, 88–92. https://doi.org/10.1093/jamia/ocx148.
464. King, Z.; Moskowitz, J.; Wakschlag, L.; Alshurafa, N. Predicting Perceived Stress Through Mirco-EMAs and a Flexible Wearable ECG Device. In Proceedings of the Proceedings of the 2018 ACM International Joint Conference and 2018 International Symposium on Pervasive and Ubiquitous Computing and Wearable Computers; Association for Computing Machinery: New York, NY, USA, 2018; UbiComp '18, pp. 106–109. https://doi.org/10.1145/3267305.3267639.
465. Jeckel, S.; Sudeck, G. Sport activities in daily routine. *German Journal of Exercise and Sport Research* **2018**, *48*, 26–39. https://doi.org/10.1007/s12662-017-0469-9.
466. Rodrigues, S.; Kaiseler, M.; Pimentel, G.; Rodrigues, J.; Aguiar, A.; Queirós, C.; Cunha, J.P.S. Ambulatory Assessment of Psychophysiological Stress among Police Officers: a Proof-of-Concept Study. *Occupational Health Science* **2018**, *2*, 215–231. https://doi.org/10.1007/s41542-017-0008-y.
467. McClure, E.A.; Tomko, R.L.; Carpenter, M.J.; Treiber, F.A.; Gray, K.M. Acceptability and compliance with a remote monitoring system to track smoking and abstinence among young smokers. *The American Journal of Drug and Alcohol Abuse* **2018**, *44*, 561–570. https://doi.org/10.1080/00952990.2018.1467431.
468. Marquet, O.; Alberico, C.; Adlakha, D.; Hipp, J.A. Examining Motivations to Play Pokémon GO and Their Influence on Perceived Outcomes and Physical Activity. *JMIR serious games* **2017**, *5*, e21. https://doi.org/10.2196/games.8048.
469. Staples, P.; Torous, J.; Barnett, I.; Carlson, K.; Sandoval, L.; Keshavan, M.; Onnela, J.P. A comparison of passive and active estimates of sleep in a cohort with schizophrenia. *NPJ schizophrenia* **2017**, *3*, 37. https://doi.org/10.1038/s41537-017-0038-0.
470. Jones, M.; Taylor, A.; Liao, Y.; Intille, S.S.; Dunton, G.F. Real-time subjective assessment of psychological stress: Associations with objectively-measured physical activity levels. *Psychology of Sport and Exercise* **2017**, *31*, 79–87. https://doi.org/10.1016/j.psychsport.2017.03.013.
471. Bae, S.; Ferreira, D.; Suffoletto, B.; Puyana, J.C.; Kurtz, R.; Chung, T.; Dey, A.K. Detecting Drinking Episodes in Young Adults Using Smartphone-based Sensors. *Proceedings of the ACM on interactive, mobile, wearable and ubiquitous technologies* **2017**, *1*, 5. https://doi.org/10.1145/3090051.
472. van Wel, L.; Huss, A.; Bachmann, P.; Zahner, M.; Kromhout, H.; Fröhlich, J.; Vermeulen, R. Context-sensitive ecological momentary assessments; integrating real-time exposure measurements, data-analytics and health assessment using a smartphone application. *Environment International* **2017**, *103*, 8–12. https://doi.org/10.1016/j.envint.2017.03.016.
473. Pendergast, F.J.; Ridgers, N.D.; Worsley, A.; McNaughton, S.A. Evaluation of a smartphone food diary application using objectively measured energy expenditure. *The International Journal of Behavioral Nutrition and Physical Activity* **2017**, *14*, 30. https://doi.org/10.1186/s12966-017-0488-9.

474. Cabrita, M.; Lousberg, R.; Tabak, M.; Hermens, H.J.; Vollenbroek-Hutten, M.M.R. An exploratory study on the impact of daily activities on the pleasure and physical activity of older adults. *European Review of Aging and Physical Activity: Official Journal of the European Group for Research into Elderly and Physical Activity* **2017**, *14*, 1. https://doi.org/10.1186/s11556-016-0170-2.
475. Byrnes, H.F.; Miller, B.A.; Morrison, C.N.; Wiebe, D.J.; Woychik, M.; Wiehe, S.E. Association of environmental indicators with teen alcohol use and problem behavior: Teens' observations vs. objectively-measured indicators. *Health & Place* **2017**, *43*, 151–157. https://doi.org/10.1016/j.healthplace.2016.12.004.
476. Ghosh, S.; Ganguly, N.; Mitra, B.; De, P. TapSense: combining self-report patterns and typing characteristics for smartphone based emotion detection. In Proceedings of the Proceedings of the 19th International Conference on Human-Computer Interaction with Mobile Devices and Services; Association for Computing Machinery: New York, NY, USA, 2017; MobileHCI '17, pp. 1–12. https://doi.org/10.1145/3098279.3098564.
477. Ghosh, S.; Ganguly, N.; Mitra, B.; De, P. Towards designing an intelligent experience sampling method for emotion detection. In Proceedings of the 2017 14th IEEE Annual Consumer Communications & Networking Conference (CCNC), 2017, pp. 401–406. https://doi.org/10.1109/CCNC.2017.7983143.
478. Brannon, E.E.; Cushing, C.C.; Crick, C.J.; Mitchell, T.B. The promise of wearable sensors and ecological momentary assessment measures for dynamical systems modeling in adolescents: a feasibility and acceptability study. *Translational Behavioral Medicine* **2016**, *6*, 558–565. https://doi.org/10.1007/s13142-016-0442-4.
479. Fisher, J.M.; Hammerla, N.Y.; Ploetz, T.; Andras, P.; Rochester, L.; Walker, R.W. Unsupervised home monitoring of Parkinson's disease motor symptoms using body-worn accelerometers. *Parkinsonism & Related Disorders* **2016**, *33*, 44–50. https://doi.org/10.1016/j.parkreldis.2016.09.009.
480. Blaauw, F.J.; Schenk, H.M.; Jeronimus, B.F.; van der Krieke, L.; de Jonge, P.; Aiello, M.; Emerencia, A.C. Let's get Physiqual - An intuitive and generic method to combine sensor technology with ecological momentary assessments. *Journal of Biomedical Informatics* **2016**, *63*, 141–149. https://doi.org/10.1016/j.jbi.2016.08.001.
481. Naughton, F.; Hopewell, S.; Lathia, N.; Schalbroeck, R.; Brown, C.; Mascolo, C.; McEwen, A.; Sutton, S. A Context-Sensing Mobile Phone App (Q Sense) for Smoking Cessation: A Mixed-Methods Study. *JMIR mHealth and uHealth* **2016**, *4*, e106. https://doi.org/10.2196/mhealth.5787.
482. Tan, X.; Alén, M.; Wiklund, P.; Partinen, M.; Cheng, S. Effects of aerobic exercise on home-based sleep among overweight and obese men with chronic insomnia symptoms: a randomized controlled trial. *Sleep Medicine* **2016**, *25*, 113–121. https://doi.org/10.1016/j.sleep.2016.02.010.
483. Hung, G.C.L.; Yang, P.C.; Chang, C.C.; Chiang, J.H.; Chen, Y.Y. Predicting Negative Emotions Based on Mobile Phone Usage Patterns: An Exploratory Study. *JMIR research protocols* **2016**, *5*, e160. https://doi.org/10.2196/resprot.5551.
484. Niermann, C.Y.N.; Herrmann, C.; von Haaren, B.; van Kann, D.; Woll, A. Affect and Subsequent Physical Activity: An Ambulatory Assessment Study Examining the Affect-Activity Association in a Real-Life Context. *Frontiers in Psychology* **2016**, *7*, 677. https://doi.org/10.3389/fpsyg.2016.00677.
485. Zang, J.; Song, J.; Wang, Z.; Yao, C.; Ma, J.; Huang, C.; Zhu, Z.; Smith, L.P.; Du, S.; Hua, J.; et al. Acceptability and feasibility of smartphone-assisted 24 h recalls in the Chinese population. *Public Health Nutrition* **2015**, *18*, 3272–3277. https://doi.org/10.1017/S1368980015000907.
486. Bond, D.S.; Thomas, J.G.; O'Leary, K.C.; Lipton, R.B.; Peterlin, B.L.; Roth, J.; Rathier, L.; Wing, R.R. Objectively measured physical activity in obese women with and without migraine. *Cephalalgia: An International Journal of Headache* **2015**, *35*, 886–893. https://doi.org/10.1177/0333102414562970.
487. Kennedy, A.P.; Epstein, D.H.; Jobes, M.L.; Agage, D.; Tyburski, M.; Phillips, K.A.; Ali, A.A.; Bari, R.; Hossain, S.M.; Hovsepian, K.; et al. Continuous in-the-field measurement of heart rate: Correlates of drug use, craving, stress, and mood in polydrug users. *Drug and Alcohol Dependence* **2015**, *151*, 159–166. https://doi.org/10.1016/j.drugalcdep.2015.03.024.
488. Liao, Y.; Intille, S.S.; Dunton, G.F. Using Ecological Momentary Assessment to Understand Where and With Whom Adults' Physical and Sedentary Activity Occur. *International Journal of Behavioral Medicine* **2015**, *22*, 51–61. https://doi.org/10.1007/s12529-014-9400-z.
489. Watkins, K.L.; Regan, S.D.; Nguyen, N.; Businelle, M.S.; Kendzor, D.E.; Lam, C.; Balis, D.; Cuevas, A.G.; Cao, Y.; Reitzel, L.R. Advancing cessation research by integrating EMA and geospatial methodologies: associations between tobacco retail outlets and real-time smoking urges during a quit attempt. *Nicotine & Tobacco Research: Official Journal of the Society for Research on Nicotine and Tobacco* **2014**, *16 Suppl 2*, S93–101. https://doi.org/10.1093/ntr/ntt135.
490. Tilley, P.J.M.; Rees, C.S. A clinical case study of the use of ecological momentary assessment in obsessive compulsive disorder. *Frontiers in Psychology* **2014**, *5*, 339. https://doi.org/10.3389/fpsyg.2014.00339.
491. Reitzel, L.R.; Kendzor, D.E.; Nguyen, N.; Regan, S.D.; Okuyemi, K.S.; Castro, Y.; Wetter, D.W.; Businelle, M.S. Shelter proximity and affect among homeless smokers making a quit attempt. *American Journal of Health Behavior* **2014**, *38*, 161–169. https://doi.org/10.5993/AJHB.38.2.1.
492. Liao, Y.; Intille, S.; Wolch, J.; Pentz, M.A.; Dunton, G.F. Understanding the physical and social contexts of children's nonschool sedentary behavior: an ecological momentary assessment study. *Journal of Physical Activity & Health* **2014**, *11*, 588–595. https://doi.org/10.1123/jpah.2011-0363.
493. Dunton, G.F.; Dzubur, E.; Kawabata, K.; Yanez, B.; Bo, B.; Intille, S. Development of a smartphone application to measure physical activity using sensor-assisted self-report. *Frontiers in Public Health* **2014**, *2*, 12. https://doi.org/10.3389/fpubh.2014.00012.

494. Dulin, P.L.; Gonzalez, V.M.; Campbell, K. Results of a pilot test of a self-administered smartphone-based treatment system for alcohol use disorders: usability and early outcomes. *Substance Abuse* **2014**, *35*, 168–175. https://doi.org/10.1080/08897077.2013.821437.
495. Runyan, J.D.; Steenbergh, T.A.; Bainbridge, C.; Daugherty, D.A.; Oke, L.; Fry, B.N. A smartphone ecological momentary assessment/intervention "app" for collecting real-time data and promoting self-awareness. *PloS One* **2013**, *8*, e71325. https://doi.org/10.1371/journal.pone.0071325.
496. Synnott, J.; Chen, L.; Nugent, C.D.; Moore, G. WiiPD—Objective Home Assessment of Parkinson's Disease Using the Nintendo Wii Remote. *IEEE Transactions on Information Technology in Biomedicine* **2012**, *16*, 1304–1312. https://doi.org/10.1109/TITB.2012.2215878.
497. Martin, C.K.; Correa, J.B.; Han, H.; Allen, H.R.; Rood, J.C.; Champagne, C.M.; Gunturk, B.K.; Bray, G.A. Validity of the Remote Food Photography Method (RFPM) for estimating energy and nutrient intake in near real-time. *Obesity (Silver Spring, Md.)* **2012**, *20*, 891–899. https://doi.org/10.1038/oby.2011.344.
498. Dunton, G.F.; Liao, Y.; Intille, S.S.; Spruijt-Metz, D.; Pentz, M. Investigating Children's Physical Activity and Sedentary Behavior Using Ecological Momentary Assessment With Mobile Phones. *Obesity* **2011**, *19*, 1205–1212. https://doi.org/10.1038/oby.2010.302.
499. Andersson, C.; Söderpalm Gordh, A.H.V.; Berglund, M. Use of Real-Time Interactive Voice Response in a Study of Stress and Alcohol Consumption. *Alcoholism: Clinical and Experimental Research* **2007**, *31*, 1908–1912. https://doi.org/10.1111/j.1530-0277.2007.00520.x.
500. Cole, C.A.; Anshari, D.; Lambert, V.; Thrasher, J.F.; Valafar, H. Detecting Smoking Events Using Accelerometer Data Collected Via Smartwatch Technology: Validation Study. *JMIR mHealth and uHealth* **2017**, *5*, e189. https://doi.org/10.2196/mhealth.9035.
501. Burns, M.N.; Begale, M.; Duffecy, J.; Gergle, D.; Karr, C.J.; Giangrande, E.; Mohr, D.C. Harnessing Context Sensing to Develop a Mobile Intervention for Depression. *Journal of Medical Internet Research* **2011**, *13*, e55. https://doi.org/10.2196/jmir.1838.
502. Saeb, S.; Zhang, M.; Kwasny, M.M.; Karr, C.J.; Kording, K.; Mohr, D.C. The Relationship between Clinical, Momentary, and Sensor-based Assessment of Depression. *International Conference on Pervasive Computing Technologies for Healthcare : [proceedings]. International Conference on Pervasive Computing Technologies for Healthcare* **2015**, *2015*, 10.4108/icst.pervasivehealth.2015.259034.
503. von Stumm, S. Feeling low, thinking slow? Associations between situational cues, mood and cognitive function. *Cognition and Emotion* **2018**, *32*, 1545–1558. https://doi.org/10.1080/02699931.2017.1420632.
504. Gaudiano, B.A.; Ellenberg, S.; Price, L.H.; Moitra, E. Time-Lagged Predictors of Daily Medication Nonadherence Beliefs during the Month Post-Hospital Discharge in Patients with Psychotic-Spectrum Disorders. *Psychiatry research* **2018**, *270*, 253–256. https://doi.org/10.1016/j.psychres.2018.09.048.
505. Preston, K.L.; Schroeder, J.R.; Kowalczyk, W.J.; Phillips, K.A.; Jobes, M.L.; Dwyer, M.; Vahabzadeh, M.; Lin, J.L.; Mezghanni, M.; Epstein, D.H. End-of-day reports of daily hassles and stress in men and women with opioid-use disorder: Relationship to momentary reports of opioid and cocaine use and stress. *Drug and Alcohol Dependence* **2018**, *193*, 21–28. https://doi.org/10.1016/j.drugalcdep.2018.08.023.
506. Sainsbury, K.; Vieira, R.; Walburn, J.; Sniehotta, F.F.; Sarkany, R.; Weinman, J.; Araujo-Soares, V. Understanding and predicting a complex behavior using n-of-1 methods: Photoprotection in xeroderma pigmentosum. *Health Psychology*, *37*, 1145. https://doi.org/10.1037/hea0000673.
507. Lindsay, E.K.; Chin, B.; Greco, C.M.; Young, S.; Brown, K.W.; Wright, A.G.C.; Smyth, J.M.; Burkett, D.; Creswell, J.D. How mindfulness training promotes positive emotions: Dismantling acceptance skills training in two randomized controlled trials. *Journal of Personality and Social Psychology*, *115*, 944. https://doi.org/10.1037/pspa0000134.
508. Chmurzynska, A.; Mlodzik-Czyzewska, M.A.; Malinowska, A.M.; Czarnocinska, J.; Wiebe, D.J. Use of a Smartphone Application Can Improve Assessment of High-Fat Food Consumption in Overweight Individuals. *Nutrients* **2018**, *10*, 1692. https://doi.org/10.3390/nu10111692.
509. Todd, K.R.; Martin Ginis, K.A. An examination of diurnal variations in neuropathic pain and affect, on exercise and non-exercise days, in adults with spinal cord injury. *Spinal Cord Series and Cases* **2018**, *4*, 1–9. https://doi.org/10.1038/s41394-018-0130-3.
510. Langer, S.L.; Romano, J.M.; Todd, M.; Strauman, T.J.; Keefe, F.J.; Syrjala, K.L.; Bricker, J.B.; Ghosh, N.; Burns, J.W.; Bolger, N.; et al. Links Between Communication and Relationship Satisfaction Among Patients With Cancer and Their Spouses: Results of a Fourteen-Day Smartphone-Based Ecological Momentary Assessment Study. *Frontiers in Psychology* **2018**, *9*, 1843. https://doi.org/10.3389/fpsyg.2018.01843.
511. Farris, S.G.; Thomas, J.G.; Abrantes, A.M.; Lipton, R.B.; Pavlovic, J.; Smitherman, T.A.; Irby, M.B.; Penzien, D.B.; Roth, J.; Leary, K.C.O.; et al. Pain worsening with physical activity during migraine attacks in women with overweight/obesity: A prospective evaluation of frequency, consistency, and correlates. *Cephalalgia : an international journal of headache* **2018**, *38*, 1707–1715. https://doi.org/10.1177/0333102417747231.
512. Yang, Y.S.; Ryu, G.W.; Han, I.; Oh, S.; Choi, M. Ecological Momentary Assessment Using Smartphone-Based Mobile Application for Affect and Stress Assessment. *Healthcare Informatics Research* **2018**, *24*, 381–386. https://doi.org/10.4258/hir.2018.24.4.381.
513. Wouters, S.; Thewissen, V.; Duif, M.; van Bree, R.J.; Lechner, L.; Jacobs, N. Habit strength and between-meal snacking in daily life: the moderating role of level of education. *Public Health Nutrition* **2018**, *21*, 2595–2605. https://doi.org/10.1017/S1368980018001283.

514. Suso-Ribera, C.; Castilla, D.; Zaragozá, I.; Ribera-Canudas, M.V.; Botella, C.; García-Palacios, A. Validity, Reliability, Feasibility, and Usefulness of Pain Monitor: A Multidimensional Smartphone App for Daily Monitoring of Adults With Heterogenous Chronic Pain. *The Clinical Journal of Pain* **2018**, *34*, 900–908. https://doi.org/10.1097/AJP.0000000000000618.
515. Bouvard, A.; Dupuy, M.; Schweitzer, P.; Revranche, M.; Fatseas, M.; Serre, F.; Misdrahi, D.; Auriacombe, M.; Swendsen, J. Feasibility and validity of mobile cognitive testing in patients with substance use disorders and healthy controls. *The American Journal on Addictions* **2018**, *27*, 553–556. https://doi.org/10.1111/ajad.12804.
516. Lambert, V.; Ferguson, S.G.; Niederdeppe, J.; Hammond, D.; Hardin, J.W.; Thrasher, J.F. Exploring the impact of efficacy messages on cessation-related outcomes using Ecological Momentary Assessment. *Tobacco Induced Diseases* **2018**, *16*, 44. https://doi.org/10.18332/tid/94460.
517. Koslovsky, M.D.; Hébert, E.T.; Swartz, M.D.; Chan, W.; Leon-Novelo, L.; Wilkinson, A.V.; Kendzor, D.E.; Businelle, M.S. The Time-Varying Relations Between Risk Factors and Smoking Before and After a Quit Attempt. *Nicotine & Tobacco Research* **2018**, *20*, 1231–1236. https://doi.org/10.1093/ntr/ntx225.
518. Baker, A.W.; Losiewicz, O.M.; Hellberg, S.N.; Simon, N.M. Integrating Technology into Clinical Care to Improve Outcomes in Panic Disorder: Use of Safety Behaviors and Resulting Anxiety as Assessed by Smartphone-Based Experience Sampling Methods. *Iproceedings* **2018**, *4*, e11788. https://doi.org/10.2196/11788.
519. Versluis, A.; Verkuil, B.; Spinhoven, P.; F Brosschot, J. Effectiveness of a smartphone-based worry-reduction training for stress reduction: A randomized-controlled trial. *Psychology & Health* **2018**, *33*, 1079–1099. https://doi.org/10.1080/08870446.2018.1456660.
520. Preston, K.L.; Kowalczyk, W.J.; Phillips, K.A.; Jobes, M.L.; Vahabzadeh, M.; Lin, J.L.; Mezghanni, M.; Epstein, D.H. Before and After: Craving, Mood, and Background Stress in the Hours Surrounding Drug Use and Stressful Events in Patients with Opioid-Use Disorder. *Psychopharmacology* **2018**, *235*, 2713–2723. https://doi.org/10.1007/s00213-018-4966-9.
521. Reback, C.J.; Rünger, D.; Fletcher, J.B.; Swendeman, D. Ecological Momentary Assessments for Self-Monitoring and Counseling to Optimize Methamphetamine Treatment and Sexual Risk Reduction Outcomes among Gay and Bisexual Men. *Journal of substance abuse treatment* **2018**, *92*, 17–26. https://doi.org/10.1016/j.jsat.2018.06.005.
522. Goldstein, S.P.; Zhang, F.; Thomas, J.G.; Butryn, M.L.; Herbert, J.D.; Forman, E.M. Application of Machine Learning to Predict Dietary Lapses During Weight Loss. *Journal of Diabetes Science and Technology* **2018**, *12*, 1045–1052. https://doi.org/10.1177/1932296818775757.
523. Swendeman, D.; Comulada, W.S.; Koussa, M.; Worthman, C.M.; Estrin, D.; Rotheram-Borus, M.J.; Ramanathan, N. Longitudinal Validity and Reliability of Brief Smartphone Self-Monitoring of Diet, Stress, and Physical Activity in a Diverse Sample of Mothers. *JMIR mHealth and uHealth* **2018**, *6*, e176. https://doi.org/10.2196/mhealth.9378.
524. Diaz, K.M.; Thanataveerat, A.; Parsons, F.E.; Yoon, S.; Cheung, Y.K.; Alcantara, C.; Duran, A.T.; Ensari, I.; Krupka, D.J.; Schwartz, J.E.; et al. The Influence of Daily Stress on Sedentary Behavior: Group and Person (N of 1) Level Results of a 1-Year Observational Study. *Psychosomatic medicine* **2018**, *80*, 620–627. https://doi.org/10.1097/PSY.0000000000000610.
525. Mead, E.L.; Chen, J.C.; Kirchner, T.R.; Butler, J.; Feldman, R.H. An Ecological Momentary Assessment of Cigarette and Cigar Dual Use Among African American Young Adults. *Nicotine & Tobacco Research* **2018**, *20*, S12–S21. https://doi.org/10.1093/ntr/nty061.
526. Robinson, C.D.; Muench, C.; Brede, E.; Endrighi, R.; Szeto, E.H.; Sells, J.R.; Lammers, J.P.; Okuyemi, K.S.; Izmirlian, G.; Waters, A.J. Pro-Tobacco Advertisement Exposure Among African American Smokers: An Ecological Momentary Assessment Study. *Addictive behaviors* **2018**, *83*, 142–147. https://doi.org/10.1016/j.addbeh.2017.10.015.
527. Lipperman-Kreda, S.; Finan, L.J.; Grube, J.W. Social and Situational Characteristics Associated with Adolescents' Drinking at Party and Non-Party Events. *Addictive behaviors* **2018**, *83*, 148–153. https://doi.org/10.1016/j.addbeh.2017.12.001.
528. Paolillo, E.W.; Obermeit, L.C.; Tang, B.; Depp, C.A.; Vaida, F.; Moore, D.J.; Moore, R.C. Smartphone-based ecological momentary assessment (EMA) of alcohol and cannabis use in older adults with and without HIV infection. *Addictive behaviors* **2018**, *83*, 102–108. https://doi.org/10.1016/j.addbeh.2017.10.016.
529. Shrier, L.A.; Burke, P.J.; Kells, M.; Scherer, E.A.; Sarda, V.; Jonestrask, C.; Xuan, Z.; Harris, S.K. Pilot randomized trial of MOMENT, a motivational counseling-plus-ecological momentary intervention to reduce marijuana use in youth. *mHealth* **2018**, *4*, 29. https://doi.org/10.21037/mhealth.2018.07.04.
530. Wright, C.; Dietze, P.M.; Agius, P.A.; Kuntsche, E.; Livingston, M.; Black, O.C.; Room, R.; Hellard, M.; Lim, M.S. Mobile Phone-Based Ecological Momentary Intervention to Reduce Young Adults' Alcohol Use in the Event: A Three-Armed Randomized Controlled Trial. *JMIR mHealth and uHealth* **2018**, *6*, e149. https://doi.org/10.2196/mhealth.9324.
531. Nes, A.A.G.; van Dulmen, S.; Brembo, E.A.; Eide, H. An mHealth Intervention for Persons with Diabetes Type 2 Based on Acceptance and Commitment Therapy Principles: Examining Treatment Fidelity. *JMIR mHealth and uHealth* **2018**, *6*, e151. https://doi.org/10.2196/mhealth.9942.
532. Roggeveen, S.; van Os, J.; Bemelmans, K.; van Poll, M.; Lousberg, R. Investigating Associations Between Changes in Mobile Phone Use and Emotions Using the Experience Sampling Method: Pilot Study. *JMIR Formative Research* **2018**, *2*, e12. https://doi.org/10.2196/formative.8499.
533. Levinson, C.A.; Sala, M.; Fewell, L.; Brosof, L.C.; Fournier, L.; Lenze, E.J. Meal and Snack-time Eating Disorder Cognitions Predict Eating Disorder Behaviors and Vice Versa in a Treatment Seeking Sample: A Mobile Technology Based Ecological Momentary Assessment Study. *Behaviour research and therapy* **2018**, *105*, 36–42. https://doi.org/10.1016/j.brat.2018.03.008.

534. Mackesy-Amiti, M.E.; Boodram, B. Feasibility of ecological momentary assessment to study mood and risk behavior among young people who inject drugs. *Drug and alcohol dependence* **2018**, *187*, 227–235. https://doi.org/10.1016/j.drugalcdep.2018.03.016.
535. Li, J.J.; Lansford, J.E. A Smartphone-Based Ecological Momentary Assessment of Parental Behavioral Consistency: Associations with Parental Stress and Child ADHD Symptoms. *Developmental psychology* **2018**, *54*, 1086–1098. https://doi.org/10.1037/dev0000516.
536. Bracci, A.; Djukic, G.; Favero, L.; Salmaso, L.; Guarda-Nardini, L.; Manfredini, D. Frequency of awake bruxism behaviours in the natural environment. A 7-day, multiple-point observation of real-time report in healthy young adults. *Journal of Oral Rehabilitation* **2018**, *45*, 423–429. https://doi.org/10.1111/joor.12627.
537. Riordan, B.C.; Moradi, S.; Carey, K.B.; Conner, T.S.; Jang, K.; Reid, K.E.; Scarf, D. Effectiveness of a Combined Web-Based and Ecological Momentary Intervention for Incoming First-Year University Students: Protocol for a 3-Arm Randomized Controlled Trial. *JMIR Research Protocols* **2018**, *7*, e10164. https://doi.org/10.2196/10164.
538. Paolillo, E.W.; Tang, B.; Depp, C.A.; Rooney, A.S.; Vaida, F.; Kaufmann, C.N.; Mausbach, B.T.; Moore, D.J.; Moore, R.C. Temporal Associations Between Social Activity and Mood, Fatigue, and Pain in Older Adults With HIV: An Ecological Momentary Assessment Study. *JMIR Mental Health* **2018**, *5*, e38. https://doi.org/10.2196/mental.9802.
539. Pramana, G.; Parmanto, B.; Lomas, J.; Lindhiem, O.; Kendall, P.C.; Silk, J. Using Mobile Health Gamification to Facilitate Cognitive Behavioral Therapy Skills Practice in Child Anxiety Treatment: Open Clinical Trial. *JMIR Serious Games* **2018**, *6*, e9. https://doi.org/10.2196/games.8902.
540. Ashurst, J.; van Woerden, I.; Dunton, G.; Todd, M.; Ohri-Vachaspati, P.; Swan, P.; Bruening, M. The Association among Emotions and Food Choices in First-Year College Students Using mobile-Ecological Momentary Assessments. *BMC Public Health* **2018**, *18*, 573. https://doi.org/10.1186/s12889-018-5447-0.
541. Doogan, N.J.; Cooper, S.; Quisenberry, A.J.; Brasky, T.M.; Browning, C.R.; Klein, E.G.; Hinton, A.; Nagaraja, H.N.; Xi, W.; Wewers, M.E. The role of travel distance and price promotions in tobacco product purchase quantity. *Health & place* **2018**, *51*, 151–157. https://doi.org/10.1016/j.healthplace.2018.03.009.
542. Jones, A.; Tiplady, B.; Houben, K.; Nederkoorn, C.; Field, M. Do daily fluctuations in inhibitory control predict alcohol consumption? An ecological momentary assessment study. *Psychopharmacology* **2018**, *235*, 1487–1496. https://doi.org/10.1007/s00213-018-4860-5.
543. Clark, D.O.; Srinivas, P.; Bodke, K.; Keith, N.; Hood, S.; Tu, W. Addressing People and Place Microenvironments in Weight Loss Disparities (APP-Me): Design of a randomized controlled trial testing timely messages for weight loss behavior in low income black and white women. *Contemporary clinical trials* **2018**, *67*, 74–80. https://doi.org/10.1016/j.cct.2018.01.006.
544. Kranzler, A.; Fehling, K.B.; Lindqvist, J.; Brillante, J.; Yuan, F.; Gao, X.; Miller, A.L.; Selby, E.A. An Ecological Investigation of the Emotional Context Surrounding Nonsuicidal Self-Injurious Thoughts and Behaviors in Adolescents and Young Adults. *Suicide and Life-Threatening Behavior* **2018**, *48*, 149–159. https://doi.org/10.1111/sltb.12373.
545. Wouters, S.; Jacobs, N.; Duif, M.; Lechner, L.; Thewissen, V. Affect and between-meal snacking in daily life: the moderating role of gender and age. *Psychology & Health* **2018**, *33*, 555–572. https://doi.org/10.1080/08870446.2017.1380813.
546. Most, J.; Vallo, P.M.; Altazan, A.D.; Gilmore, L.A.; Sutton, E.F.; Cain, L.E.; Burton, J.H.; Martin, C.K.; Redman, L.M. Food Photography Is Not an Accurate Measure of Energy Intake in Obese, Pregnant Women. *The Journal of Nutrition* **2018**, *148*, 658–663. https://doi.org/10.1093/jn/nxy009.
547. Santa Maria, D.; Padhye, N.; Yang, Y.; Gallardo, K.; Businelle, M. Predicting Sexual Behaviors Among Homeless Young Adults: Ecological Momentary Assessment Study. *JMIR Public Health and Surveillance* **2018**, *4*, e39. https://doi.org/10.2196/publichealth.9020.
548. Wouters, S.; Jacobs, N.; Duif, M.; Lechner, L.; Thewissen, V. Negative affective stress reactivity: The dampening effect of snacking. *Stress and Health* **2018**, *34*, 286–295. https://doi.org/10.1002/smi.2788.
549. Mason, A.E.; Jhaveri, K.; Cohn, M.; Brewer, J.A. Testing a Mobile Mindful Eating Intervention Targeting Craving-Related Eating: Feasibility and Proof of Concept. *Journal of behavioral medicine* **2018**, *41*, 160–173. https://doi.org/10.1007/s10865-017-9884-5.
550. Jongeneel, A.; Scheffers, D.; Tromp, N.; Nuij, C.; Delespaul, P.; Riper, H.; van der Gaag, M.; van den Berg, D. Reducing distress and improving social functioning in daily life in people with auditory verbal hallucinations: study protocol for the 'Temstem' randomised controlled trial. *BMJ Open* **2018**, *8*, e020537. https://doi.org/10.1136/bmjopen-2017-020537.
551. Hébert, E.T.; Stevens, E.M.; Frank, S.G.; Kendzor, D.E.; Wetter, D.W.; Zvolensky, M.J.; Buckner, J.D.; Businelle, M.S. AN ECOLOGICAL MOMENTARY INTERVENTION FOR SMOKING CESSATION: THE ASSOCIATIONS OF JUST-IN-TIME, TAILORED MESSAGES WITH LAPSE RISK FACTORS. *Addictive behaviors* **2018**, *78*, 30–35. https://doi.org/10.1016/j.addbeh.2017.10.026.
552. Moran, E.K.; Culbreth, A.J.; Barch, D.M. Emotion Regulation Predicts Everyday Emotion Experience and Social Function in Schizophrenia. *Clinical psychological science : a journal of the Association for Psychological Science* **2018**, *6*, 271–279. https://doi.org/10.1177/2167702617738827.
553. Henry, S.L.; Jamner, L.D.; Choi, S.E.; Pahl, M.V. THE EFFECT OF THE INTERDIALYTIC INTERVAL ON COGNITIVE FUNCTION IN PATIENTS ON HAEMODIALYSIS. *Journal of renal care* **2018**, *44*, 44–51. https://doi.org/10.1111/jorc.12231.
554. Vork, L.; Keszthelyi, D.; Mujagic, Z.; Kruimel, J.W.; Leue, C.; Pontén, I.; Törnblom, H.; Simrén, M.; Albu-Soda, A.; Aziz, Q.; et al. Development, content validity, and cross-cultural adaptation of a patient-reported outcome measure for real-time symptom assessment in irritable bowel syndrome. *Neurogastroenterology & Motility* **2018**, *30*, e13244. https://doi.org/10.1111/nmo.13244.

555. MacLean, R.R.; Pincus, A.L.; Smyth, J.M.; Geier, C.F.; Wilson, S.J. Extending the Balloon Analogue Risk Task to Assess Naturalistic Risk Taking via a Mobile Platform. *Journal of psychopathology and behavioral assessment* **2018**, *40*, 107–116. https://doi.org/10.1007/s10862-017-9628-4.
556. Han, H.; Zhang, J.Y.; Hser, Y.I.; Liang, D.; Li, X.; Wang, S.S.; Du, J.; Zhao, M. Feasibility of a Mobile Phone App to Support Recovery From Addiction in China: Secondary Analysis of a Pilot Study. *JMIR mHealth and uHealth* **2018**, *6*, e46. https://doi.org/10.2196/mhealth.8388.
557. Kumar, D.; Tully, L.M.; Iosif, A.M.; Zakskorn, L.N.; Nye, K.E.; Zia, A.; Niendam, T.A. A Mobile Health Platform for Clinical Monitoring in Early Psychosis: Implementation in Community-Based Outpatient Early Psychosis Care. *JMIR Mental Health* **2018**, *5*, e15. https://doi.org/10.2196/mental.8551.
558. Suso-Ribera, C.; Mesas, ; Medel, J.; Server, A.; Márquez, E.; Castilla, D.; Zaragozá, I.; García-Palacios, A. Improving pain treatment with a smartphone app: study protocol for a randomized controlled trial. *Trials* **2018**, *19*, 145. https://doi.org/10.1186/s13063-018-2539-1.
559. Kuerbis, A.; Treloar, H.; Shao, S.; Houser, J.; Muench, F.; Morgenstern, J. Comparing daily drivers of problem drinking among older and younger adults: An electronic daily diary study using smartphones. *Drug and alcohol dependence* **2018**, *183*, 240–246. https://doi.org/10.1016/j.drugalcdep.2017.11.012.
560. Rendina, H.J.; Millar, B.M.; Parsons, J.T. The Critical Role of Internalized HIV-Related Stigma in the Daily Negative Affective Experiences of HIV-Positive Gay and Bisexual Men. *Journal of affective disorders* **2018**, *227*, 289–297. https://doi.org/10.1016/j.jad.2017.11.005.
561. Bakolis, I.; Hammoud, R.; Smythe, M.; Gibbons, J.; Davidson, N.; Tognin, S.; Mechelli, A. Urban Mind: Using Smartphone Technologies to Investigate the Impact of Nature on Mental Well-Being in Real Time. *Bioscience* **2018**, *68*, 134–145. https://doi.org/10.1093/biosci/bix149.
562. Boesen, V.B.; Christoffersen, T.; Watt, T.; Borresen, S.W.; Klose, M.; Feldt-Rasmussen, U. PlenadrEMA: effect of dual-release versus conventional hydrocortisone on fatigue, measured by ecological momentary assessments: a study protocol for an open-label switch pilot study. *BMJ Open* **2018**, *8*, e019487. https://doi.org/10.1136/bmjopen-2017-019487.
563. Meyer, T.; Quaedflieg, C.W.E.M.; Weijland, K.; Schruers, K.; Merckelbach, H.; Smeets, T. Frontal EEG asymmetry during symptom provocation predicts subjective responses to intrusions in survivors with and without PTSD. *Psychophysiology* **2018**, *55*, e12779. https://doi.org/10.1111/psyp.12779.
564. Hébert, E.T.; Vandewater, E.A.; Businelle, M.S.; Harrell, M.B.; Kelder, S.H.; Perry, C.L. Real Time Assessment of Young Adults' Attitudes toward Tobacco Messages. *Tobacco regulatory science* **2018**, *4*, 644–655. https://doi.org/10.18001/TRS.4.1.10.
565. Sasaki, W.; Nakazawa, J.; Okoshi, T. Comparing ESM Timings for Emotional Estimation Model with Fine Temporal Granularity. In Proceedings of the Proceedings of the 2018 ACM International Joint Conference and 2018 International Symposium on Pervasive and Ubiquitous Computing and Wearable Computers; ACM: Singapore Singapore, 2018; pp. 722–725. https://doi.org/10.1145/3267305.3267699.
566. Harvey, E.J.; Rubin, L.F.; Smiley, S.L.; Zhou, Y.; Elmasry, H.; Pearson, J.L. Mobile Phone Ownership Is Not a Serious Barrier to Participation in Studies: Descriptive Study. *JMIR mHealth and uHealth* **2018**, *6*, e21. https://doi.org/10.2196/mhealth.8123.
567. Ludwigs, K.; Lucas, R.; Burger, M.; Veenhoven, R.; Arends, L. How Does More Attention to Subjective Well-Being Affect Subjective Well-Being? *Applied Research in Quality of Life* **2018**, *13*, 1055–1080. https://doi.org/10.1007/s11482-017-9575-y.
568. Chan, C.S.; van Tilburg, W.A.P.; Igou, E.R.; Poon, C.Y.S.; Tam, K.Y.Y.; Wong, V.U.T.; Cheung, S.K. Situational meaninglessness and state boredom: Cross-sectional and experience-sampling findings. *Motivation and Emotion* **2018**, *42*, 555–565. https://doi.org/10.1007/s11031-018-9693-3.
569. Gago-Veiga, A.B.; Pagán, J.; Henares, K.; Heredia, P.; González-García, N.; De Orbe, M.I.; Ayala, J.L.; Sobrado, M.; Vivancos, J. To what extent are patients with migraine able to predict attacks? *Journal of Pain Research* **2018**, *11*, 2083–2094. https://doi.org/10.2147/JPR.S175602.
570. George, M.J.; Russell, M.A.; Piontak, J.R.; Odgers, C.L. Concurrent and Subsequent Associations between Daily Digital Technology Use and High-Risk Adolescents' Mental Health Symptoms. *Child development* **2018**, *89*, 78–88. https://doi.org/10.1111/cdev.12819.
571. Sliwinski, M.J.; Mogle, J.A.; Hyun, J.; Munoz, E.; Smyth, J.M.; Lipton, R.B. Reliability and Validity of Ambulatory Cognitive Assessments. *Assessment* **2018**, *25*, 14–30. https://doi.org/10.1177/1073191116643164.
572. Connolly, S.L.; Alloy, L.B. Negative Event Recall as a Vulnerability for Depression: Relationship between Momentary Stress-Reactive Rumination and Memory for Daily Life Stress. *Clinical psychological science : a journal of the Association for Psychological Science* **2018**, *6*, 32–47. https://doi.org/10.1177/2167702617729487.
573. Moran, L.M.; Kowalczyk, W.J.; Phillips, K.A.; Vahabzadeh, M.; Lin, J.L.; Mezghanni, M.; Epstein, D.H.; Preston, K.L. Sex differences in daily life stress and craving in opioid-dependent patients. *The American Journal of Drug and Alcohol Abuse* **2018**, *44*, 512–523. https://doi.org/10.1080/00952990.2018.1454934.
574. Kleindienst, N.; Priebe, K.; Petri, M.; Hecht, A.; Santangelo, P.; Bohus, M.; Schulte-Herbrüggen, O. Trauma-related memories in PTSD after interpersonal violence: an ambulatory assessment study. *European Journal of Psychotraumatology* **2017**, *8*, 1409062. https://doi.org/10.1080/20008198.2017.1409062.

575. Wahl, D.R.; Villinger, K.; König, L.M.; Ziesemer, K.; Schupp, H.T.; Renner, B. Healthy food choices are happy food choices: Evidence from a real life sample using smartphone based assessments. *Scientific Reports* **2017**, *7*, 17069. https://doi.org/10.1038/s41598-017-17262-9.
576. Kirchner, T.; Magallón-Neri, E.; Ortiz, M.S.; Planellas, I.; Forns, M.; Calderón, C. Adolescents' Daily Perception of Internalizing Emotional States by Means of Smartphone-based Ecological Momentary Assessment. *The Spanish Journal of Psychology* **2017**, *20*, E71. https://doi.org/10.1017/sjp.2017.70.
577. Everhart, R.S.; Heron, K.E.; Leibach, G.G.; Miadich, S.A. Developing a Mobile Health Intervention for Low-Income, Urban Caregivers of Children with Asthma: A Pilot Study. *Pediatric Allergy, Immunology, and Pulmonology* **2017**, *30*, 252–256. https://doi.org/10.1089/ped.2017.0794.
578. Odgers, C.L.; Russell, M.A. Violence exposure is associated with adolescents' same and next day mental health symptoms. *Journal of child psychology and psychiatry, and allied disciplines* **2017**, *58*, 1310–1318. https://doi.org/10.1111/jcpp.12763.
579. Labhart, F.; Anderson, K.G.; Kuntsche, E. The Spirit Is Willing, But the Flesh is Weak: Why Young People Drink More Than Intended on Weekend Nights—An Event-Level Study. *Alcoholism: Clinical and Experimental Research* **2017**, *41*, 1961–1969. https://doi.org/10.1111/acer.13490.
580. Inauen, J.; Bolger, N.; Shrout, P.E.; Stadler, G.; Amrein, M.; Rackow, P.; Scholz, U. Using Smartphone-Based Support Groups to Promote Healthy Eating in Daily Life: A Randomised Trial. *Applied Psychology: Health and Well-Being* **2017**, *9*, 303–323. https://doi.org/10.1111/aphw.12093.
581. Verhagen, S.J.W.; Berben, J.A.; Leue, C.; Marsman, A.; Delespaul, P.A.E.G.; van Os, J.; Lousberg, R. Demonstrating the reliability of transdiagnostic mHealth Routine Outcome Monitoring in mental health services using experience sampling technology. *PLoS ONE* **2017**, *12*, e0186294. https://doi.org/10.1371/journal.pone.0186294.
582. Connolly, S.L.; Alloy, L.B. Rumination Interacts with Life Stress to Predict Depressive Symptoms: An Ecological Momentary Assessment Study. *Behaviour research and therapy* **2017**, *97*, 86–95. https://doi.org/10.1016/j.brat.2017.07.006.
583. Lipperman-Kreda, S.; Gruenewald, P.J.; Grube, J.W.; Bersamin, M. Adolescents, alcohol, and marijuana: Context characteristics and problems associated with simultaneous use. *Drug and alcohol dependence* **2017**, *179*, 55–60. https://doi.org/10.1016/j.drugalcdep.2017.06.023.
584. Suffoletto, B.; Goyal, A.; Puyana, J.C.; Chung, T. Can an App Help Identify Psychomotor Function Impairments During Drinking Occasions in the Real World? A Mixed Method Pilot Study. *Substance abuse* **2017**, *38*, 438–449. https://doi.org/10.1080/08897077.2017.1356797.
585. Hébert, E.T.; Vandewater, E.A.; Businelle, M.S.; Harrell, M.B.; Kelder, S.H.; Perry, C.L. Feasibility and Reliability of a Mobile Tool to Evaluate Exposure to Tobacco Product Marketing and Messages Using Ecological Momentary Assessment. *Addictive behaviors* **2017**, *73*, 105–110. https://doi.org/10.1016/j.addbeh.2017.05.004.
586. Wright, A.G.; Stepp, S.D.; Scott, L.N.; Hallquist, M.N.; Beeney, J.E.; Lazarus, S.A.; Pilkonis, P.A. The Effect of Pathological Narcissism on Interpersonal and Affective Processes in Social Interactions. *Journal of abnormal psychology* **2017**, *126*, 898–910. https://doi.org/10.1037/abn0000286.
587. Hoeppner, B.B.; Hoeppner, S.S.; Kelly, L.; Schick, M.; Kelly, J.F. Smiling Instead of Smoking: Development of a Positive Psychology Smoking Cessation Smartphone App for Non-daily Smokers. *International Journal of Behavioral Medicine* **2017**, *24*, 683–693. https://doi.org/10.1007/s12529-017-9640-9.
588. Farmer, S.; Mindry, D.; Comulada, W.S.; Swendeman, D. Mobile Phone Ecological Momentary Assessment of Daily Stressors among People Living With HIV: Elucidating Factors Underlying Health-Related Challenges in Daily Routines. *The Journal of the Association of Nurses in AIDS Care : JANAC* **2017**, *28*, 737–751. https://doi.org/10.1016/j.jana.2017.04.001.
589. PONNADA, A.; HAYNES, C.; MANIAR, D.; MANJOURIDES, J.; INTILLE, S. Microinteraction Ecological Momentary Assessment Response Rates: Effect of Microinteractions or the Smartwatch? *Proceedings of the ACM on interactive, mobile, wearable and ubiquitous technologies* **2017**, *1*, 92. https://doi.org/10.1145/3130957.
590. Schweitzer, P.; Husky, M.; Allard, M.; Amieva, H.; Pérès, K.; Foubert-Samier, A.; Dartigues, J.; Swendsen, J. Feasibility and validity of mobile cognitive testing in the investigation of age-related cognitive decline. *International Journal of Methods in Psychiatric Research* **2016**, *26*, e1521. https://doi.org/10.1002/mpr.1521.
591. Preston, K.L.; Kowalczyk, W.J.; Phillips, K.A.; Jobes, M.L.; Vahabzadeh, M.; Lin, J.L.; Mezghanni, M.; Epstein, D.H. Context and Craving During Stressful Events in the Daily Lives of Drug-dependent Patients. *Psychopharmacology* **2017**, *234*, 2631–2642. https://doi.org/10.1007/s00213-017-4663-0.
592. Piontak, J.R.; Russell, M.A.; Danese, A.; Copeland, W.E.; Hoyle, R.H.; Odgers, C.L. Violence exposure and adolescents' same-day obesogenic behaviors: New findings and a replication. *Social science & medicine (1982)* **2017**, *189*, 145–151. https://doi.org/10.1016/j.socscimed.2017.07.004.
593. Fritz, H.; Tarraf, W.; Saleh, D.J.; Cutchin, M.P. Using a Smartphone-Based Ecological Momentary Assessment Protocol With Community Dwelling Older African Americans. *The Journals of Gerontology Series B: Psychological Sciences and Social Sciences* **2017**, *72*, 876–887. https://doi.org/10.1093/geronb/gbw166.
594. Rose, S.W.; Anesetti-Rothermel, A.; Elmasry, H.; Niaura, R. Young adult non-smokers' exposure to real-world tobacco marketing: results of an ecological momentary assessment pilot study. *BMC Research Notes* **2017**, *10*, 435. https://doi.org/10.1186/s13104-017-2758-7.

595. Scott, C.K.; Dennis, M.L.; Gustafson, D.H. Using smartphones to decrease substance use via self-monitoring and recovery support: study protocol for a randomized control trial. *Trials* **2017**, *18*, 374. https://doi.org/10.1186/s13063-017-2096-z.
596. Probst, T.; Pryss, R.C.; Langguth, B.; Rauschecker, J.P.; Schobel, J.; Reichert, M.; Spiliopoulou, M.; Schlee, W.; Zimmermann, J. Does Tinnitus Depend on Time-of-Day? An Ecological Momentary Assessment Study with the "TrackYourTinnitus" Application. *Frontiers in Aging Neuroscience* **2017**, *9*, 253. https://doi.org/10.3389/fnagi.2017.00253.
597. Liddle, J.; Wishink, A.; Springfield, L.; Gustafsson, L.; Ireland, D.; Silburn, P. Can smartphones measure momentary quality of life and participation? A proof of concept using experience sampling surveys with university students. *Australian Occupational Therapy Journal* **2017**, *64*, 294–304. https://doi.org/10.1111/1440-1630.12360.
598. Moore, R.C.; Kaufmann, C.N.; Rooney, A.S.; Moore, D.J.; Eyler, L.T.; Granholm, E.; Woods, S.P.; Swendsen, J.; Heaton, R.K.; Scott, J.; et al. Feasibility and Acceptability of Ecological Momentary Assessment of Daily Functioning Among Older Adults with HIV. *The American journal of geriatric psychiatry : official journal of the American Association for Geriatric Psychiatry* **2017**, *25*, 829–840. https://doi.org/10.1016/j.jagp.2016.11.019.
599. Roberts, M.E.; Lu, B.; Browning, C.R.; Ferketich, A.K. Tracking Young Adults' Attitudes toward Tobacco Marketing Using Ecological Momentary Assessment (EMA). *Substance use & misuse* **2017**, *52*, 1219–1224. https://doi.org/10.1080/10826084.2017.1302958.
600. Vansimaeys, C.; Zuber, M.; Pitrat, B.; Join-Lambert, C.; Tamazyan, R.; Farhat, W.; Bungener, C. Combining Standard Conventional Measures and Ecological Momentary Assessment of Depression, Anxiety and Coping Using Smartphone Application in Minor Stroke Population: A Longitudinal Study Protocol. *Frontiers in Psychology* **2017**, *8*, 1172. https://doi.org/10.3389/fpsyg.2017.01172.
601. Sundberg, K.; Wengström, Y.; Blomberg, K.; Hälleberg-Nyman, M.; Frank, C.; Langius-Eklöf, A. Early detection and management of symptoms using an interactive smartphone application (Interaktor) during radiotherapy for prostate cancer. *Supportive Care in Cancer* **2017**, *25*, 2195–2204. https://doi.org/10.1007/s00520-017-3625-8.
602. Kimhy, D.; Wall, M.M.; Hansen, M.C.; Vakhrusheva, J.; Choi, C.J.; Delespaul, P.; Tarrier, N.; Sloan, R.P.; Malaspina, D. Autonomic Regulation and Auditory Hallucinations in Individuals With Schizophrenia: An Experience Sampling Study. *Schizophrenia Bulletin* **2017**, *43*, 754–763. https://doi.org/10.1093/schbul/sbw219.
603. Demirci, J.R.; Bogen, D.L. Feasibility and acceptability of a mobile app in an ecological momentary assessment of early breastfeeding. *Maternal & Child Nutrition* **2016**, *13*, e12342. https://doi.org/10.1111/mcn.12342.
604. Roth, A.M.; Rossi, J.; Goldshear, J.L.; Truong, Q.; Armenta, R.F.; Lankenau, S.E.; Garfein, R.S.; Simmons, J. Potential Risks of Ecological Momentary Assessment Among Persons Who Inject Drugs. *Substance use & misuse* **2017**, *52*, 840–847. https://doi.org/10.1080/10826084.2016.1264969.
605. Patterson, P.D.; Moore, C.G.; Guyette, F.X.; Doman, J.M.; Sequeira, D.; Werman, H.A.; Swanson, D.; Hostler, D.; Lynch, J.; Russo, L.; et al. Fatigue mitigation with SleepTrackTXT2 in air medical emergency care systems: study protocol for a randomized controlled trial. *Trials* **2017**, *18*, 254. https://doi.org/10.1186/s13063-017-1999-z.
606. Holland, E.; Koval, P.; Stratemeyer, M.; Thomson, F.; Haslam, N. Sexual objectification in women's daily lives: A smartphone ecological momentary assessment study. *British Journal of Social Psychology* **2017**, *56*, 314–333. https://doi.org/10.1111/bjso.12152.
607. Wright, C.J.; Dietze, P.M.; Agius, P.A.; Kuntsche, E.; Room, R.; Livingston, M.; Hellard, M.; Lim, M.S. An Ecological Momentary Intervention to Reduce Alcohol Consumption in Young Adults Delivered During Drinking Events: Protocol for a Pilot Randomized Controlled Trial. *JMIR Research Protocols* **2017**, *6*, e95. https://doi.org/10.2196/resprot.6760.
608. Gernart, M.; Tsianakas, A.; Zeidler, C.; Riepe, C.; Osada, N.; Pihan, D.; Ständer, S. ItchApp©: An App-based eDiary for Assessment of Chronic Pruritus in Clinical Trials. *Acta Dermato Venereologica* **2017**, *97*, 601–606. https://doi.org/10.2340/00015555-2602.
609. Bidargaddi, N.; Musiat, P.; Winsall, M.; Vogl, G.; Blake, V.; Quinn, S.; Orlowski, S.; Antezana, G.; Schrader, G. Efficacy of a Web-Based Guided Recommendation Service for a Curated List of Readily Available Mental Health and Well-Being Mobile Apps for Young People: Randomized Controlled Trial. *Journal of Medical Internet Research* **2017**, *19*, e141. https://doi.org/10.2196/jmir.6775.
610. Milesi, C.; Perez-Felkner, L.; Brown, K.; Schneider, B. Engagement, Persistence, and Gender in Computer Science: Results of a Smartphone ESM Study. *Frontiers in Psychology* **2017**, *8*, 602. https://doi.org/10.3389/fpsyg.2017.00602.
611. Nahum, M.; Van Vleet, T.M.; Sohal, V.S.; Mirzabekov, J.J.; Rao, V.R.; Wallace, D.L.; Lee, M.B.; Dawes, H.; Stark-Inbar, A.; Jordan, J.T.; et al. Immediate Mood Scaler: Tracking Symptoms of Depression and Anxiety Using a Novel Mobile Mood Scale. *JMIR mHealth and uHealth* **2017**, *5*, e44. https://doi.org/10.2196/mhealth.6544.
612. Moitra, E.; Gaudiano, B.A.; Davis, C.H.; Ben-Zeev, D. Feasibility and Acceptability of Post-Hospitalization Ecological Momentary Assessment in Patients with Psychotic-Spectrum Disorders. *Comprehensive psychiatry* **2017**, *74*, 204–213. https://doi.org/10.1016/j.comppsych.2017.01.018.
613. Nes, A.A.G.; van Dulmen, S.; Wicksell, R.; Fors, E.A.; Eide, H. Analyzing Change Processes Resulting from a Smartphone Maintenance Intervention Based on Acceptance and Commitment Therapy for Women with Chronic Widespread Pain. *International Journal of Behavioral Medicine* **2017**, *24*, 215–229. https://doi.org/10.1007/s12529-016-9590-7.
614. Steffens, J.; Steele, D.; Guastavino, C. Situational and person-related factors influencing momentary and retrospective soundscape evaluations in day-to-day life. *The Journal of the Acoustical Society of America* **2017**, *141*, 1414–1425. https://doi.org/10.1121/1.4976627.

615. Barrigón, M.L.; Berrouiguet, S.; Carballo, J.J.; Bonal-Giménez, C.; Fernández-Navarro, P.; Pfang, B.; Delgado-Gómez, D.; Courtet, P.; Aroca, F.; Lopez-Castroman, J.; et al. User profiles of an electronic mental health tool for ecological momentary assessment: MEmind. *International Journal of Methods in Psychiatric Research* **2017**, *26*, e1554. https://doi.org/10.1002/mpr.1554.
616. Burke, L.E.; Shiffman, S.; Music, E.; Styn, M.A.; Kriska, A.; Smailagic, A.; Siewiorek, D.; Ewing, L.J.; Chasens, E.; French, B.; et al. Ecological Momentary Assessment in Behavioral Research: Addressing Technological and Human Participant Challenges. *Journal of Medical Internet Research* **2017**, *19*, e77. https://doi.org/10.2196/jmir.7138.
617. Conner, T.S.; Brookie, K.L.; Carr, A.C.; Mainvil, L.A.; Vissers, M.C.M. Let them eat fruit! The effect of fruit and vegetable consumption on psychological well-being in young adults: A randomized controlled trial. *PLoS ONE* **2017**, *12*, e0171206. https://doi.org/10.1371/journal.pone.0171206.
618. Carter, M.C.; Burley, V.J.; Cade, J.E. Weight Loss Associated With Different Patterns of Self-Monitoring Using the Mobile Phone App My Meal Mate. *JMIR mHealth and uHealth* **2017**, *5*, e8. https://doi.org/10.2196/mhealth.4520.
619. van der Krieke, L.; Blaauw, F.J.; Emerencia, A.C.; Schenk, H.M.; Slaets, J.P.J.; Bos, E.H.; de Jonge, P.; Jeronimus, B.F. Temporal Dynamics of Health and Well-Being: A Crowdsourcing Approach to Momentary Assessments and Automated Generation of Personalized Feedback. *Psychosomatic Medicine* **2017**, *79*, 213–223. https://doi.org/10.1097/PSY.0000000000000378.
620. Tomko, R.L.; Saladin, M.E.; McClure, E.A.; Squeglia, L.M.; Carpenter, M.J.; Tiffany, S.T.; Baker, N.L.; Gray, K.M. Alcohol consumption as a predictor of reactivity to smoking and stress cues presented in the natural environment of smokers. *Psychopharmacology* **2017**, *234*, 427–435. https://doi.org/10.1007/s00213-016-4472-x.
621. Gleeson, J.; Lederman, R.; Herrman, H.; Koval, P.; Eleftheriadis, D.; Bendall, S.; Cotton, S.M.; Alvarez-Jimenez, M. Moderated online social therapy for carers of young people recovering from first-episode psychosis: study protocol for a randomised controlled trial. *Trials* **2017**, *18*, 27. https://doi.org/10.1186/s13063-016-1775-5.
622. Worthen-Chaudhari, L.; McGonigal, J.; Logan, K.; Bockbrader, M.A.; Yeates, K.O.; Mysiw, W.J. Reducing concussion symptoms among teenage youth: Evaluation of a mobile health app. *Brain injury* **2017**, *31*, 1279–1286. https://doi.org/10.1080/02699052.2017.1332388.
623. Cheung, Y.K.; Hsueh, P.Y.S.; Qian, M.; Yoon, S.; Meli, L.; Diaz, K.M.; Schwartz, J.E.; Kronish, I.M.; Davidson, K.W. Are nomothetic or ideographic approaches superior in predicting daily exercise behaviors? Analyzing N-of-1 mHealth data. *Methods of information in medicine* **2017**, *56*, 452–460. https://doi.org/10.3414/ME16-02-0051.
624. Duncan, D.T.; Kapadia, F.; Kirchner, T.R.; Goedel, W.C.; Brady, W.J.; Halkitis, P.N. Acceptability of Ecological Momentary Assessment Among Young Men Who Have Sex with Men. *Journal of LGBT youth* **2017**, *14*, 436–444. https://doi.org/10.1080/19361653.2017.1365038.
625. Scott, S.B.; Ram, N.; Smyth, J.; Almeida, D.; Sliwinski, M. Age Differences in Negative Emotional Responses to Daily Stressors Depend on Time Since Event. *Developmental psychology* **2017**, *53*, 177–190. https://doi.org/10.1037/dev0000257.
626. Randall, W.M.; Rickard, N.S. Reasons for personal music listening: A mobile experience sampling study of emotional outcomes. *Psychology of Music* **2017**, *45*, 479–495. https://doi.org/10.1177/0305735616666939.
627. Moran, E.K.; Culbreth, A.J.; Barch, D.M. Ecological Momentary Assessment of Negative Symptoms in Schizophrenia: Relationships to Effort Based Decision Making and Reinforcement Learning. *Journal of abnormal psychology* **2017**, *126*, 96–105. https://doi.org/10.1037/abn0000240.
628. Shiyko, M.; Perkins, S.; Caldwell, L. Feasibility and Adherence Paradigm to Ecological Momentary Assessments in Urban Minority Youth. *Psychological assessment* **2017**, *29*, 926–934. https://doi.org/10.1037/pas0000386.
629. Wouters, S.; Thewissen, V.; Duif, M.; Lechner, L.; Jacobs, N. Assessing Energy Intake in Daily Life: Signal-Contingent Smartphone Application Versus Event-Contingent Paper and Pencil Estimated Diet Diary. *Psychologica Belgica*, *56*, 357–369. https://doi.org/10.5334/pb.339.
630. Schlee, W.; Pryss, R.C.; Probst, T.; Schobel, J.; Bachmeier, A.; Reichert, M.; Langguth, B. Measuring the Moment-to-Moment Variability of Tinnitus: The TrackYourTinnitus Smart Phone App. *Frontiers in Aging Neuroscience* **2016**, *8*, 294. https://doi.org/10.3389/fnagi.2016.00294.
631. Ernst, L.d.L.; Harden, C.L.; Pennell, P.B.; Llewellyn, N.; Lau, C.; Barnard, S.; Bartfeld, E.; French, J.A. Medication adherence in women with epilepsy who are planning pregnancy. *Epilepsia* **2016**, *57*, 2039–2044. https://doi.org/10.1111/epi.13586.
632. Businelle, M.S.; Ma, P.; Kendzor, D.E.; Frank, S.G.; Vidrine, D.J.; Wetter, D.W. An Ecological Momentary Intervention for Smoking Cessation: Evaluation of Feasibility and Effectiveness. *Journal of Medical Internet Research* **2016**, *18*, e321. https://doi.org/10.2196/jmir.6058.
633. Haines, S.J.; Gleeson, J.; Kuppens, P.; Hollenstein, T.; Ciarrochi, J.; Labuschagne, I.; Grace, C.; Koval, P. The Wisdom to Know the Difference: Strategy-Situation Fit in Emotion Regulation in Daily Life Is Associated With Well-Being. *Psychological Science* **2016**, *27*, 1651–1659. https://doi.org/10.1177/0956797616669086.
634. Kenny, R.; Dooley, B.; Fitzgerald, A. Ecological Momentary Assessment of Adolescent Problems, Coping Efficacy, and Mood States Using a Mobile Phone App: An Exploratory Study. *JMIR Mental Health* **2016**, *3*, e51. https://doi.org/10.2196/mental.6361.
635. Thomas, J.G.; Pavlovic, J.; Lipton, R.B.; Roth, J.; Rathier, L.; O'Leary, K.C.; Buse, D.C.; Evans, E.W.; Bond, D.S. Ecological momentary assessment of the relationship between headache pain intensity and pain interference in women with migraine and obesity. *Cephalalgia* **2016**, *36*, 1228–1237. https://doi.org/10.1177/0333102415625613.

636. Seidel, M.; Petermann, J.; Diestel, S.; Ritschel, F.; Boehm, I.; King, J.A.; Geisler, D.; Bernardoni, F.; Roessner, V.; Goschke, T.; et al. A naturalistic examination of negative affect and disorder-related rumination in anorexia nervosa. *European Child & Adolescent Psychiatry* **2016**, *25*, 1207–1216. https://doi.org/10.1007/s00787-016-0844-3.
637. Wenze, S.J.; Armey, M.F.; Weinstock, L.M.; Gaudiano, B.A.; Miller, I.W. An Open Trial of a Smartphone-Assisted, Adjunctive Intervention to Improve Treatment Adherence in Bipolar Disorder. *Journal of psychiatric practice* **2016**, *22*, 492–504. https://doi.org/10.1097/PRA.0000000000000196.
638. van Ballegooijen, W.; Ruwaard, J.; Karyotaki, E.; Ebert, D.D.; Smit, J.H.; Riper, H. Reactivity to smartphone-based ecological momentary assessment of depressive symptoms (MoodMonitor): protocol of a randomised controlled trial. *BMC Psychiatry* **2016**, *16*, 359. https://doi.org/10.1186/s12888-016-1065-5.
639. Kaufmann, C.N.; Gershon, A.; Eyler, L.T.; Depp, C.A. Clinical Significance of Mobile Health Assessed Sleep Duration and Variability in Bipolar Disorder. *Journal of psychiatric research* **2016**, *81*, 152–159. https://doi.org/10.1016/j.jpsychires.2016.07.008.
640. Businelle, M.S.; Ma, P.; Kendzor, D.E.; Frank, S.G.; Wetter, D.W.; Vidrine, D.J. Using Intensive Longitudinal Data Collected via Mobile Phone to Detect Imminent Lapse in Smokers Undergoing a Scheduled Quit Attempt. *Journal of Medical Internet Research* **2016**, *18*, e275. https://doi.org/10.2196/jmir.6307.
641. Magallón-Neri, E.; Kirchner-Nebot, T.; Forns-Santacana, M.; Calderón, C.; Planellas, I. Ecological Momentary Assessment with smartphones for measuring mental health problems in adolescents. *World Journal of Psychiatry* **2016**, *6*, 303–310. https://doi.org/10.5498/wjp.v6.i3.303.
642. Willner-Reid, J.; Whitaker, D.; Epstein, D.H.; Phillips, K.A.; Pulaski, A.; Preston, K.L.; Willner, P. Cognitive-behavioral therapy for heroin and cocaine use: ecological momentary assessment of homework simplification and compliance1. *Psychology and psychotherapy* **2016**, *89*, 276–293. https://doi.org/10.1111/papt.12080.
643. Groefsema, M.; Engels, R.; Kuntsche, E.; Smit, K.; Luijten, M. Cognitive Biases for Social Alcohol-Related Pictures and Alcohol Use in Specific Social Settings: An Event-Level Study. *Alcoholism: Clinical and Experimental Research* **2016**, *40*, 2001–2010. https://doi.org/10.1111/acer.13165.
644. Intille, S.; Haynes, C.; Maniar, D.; Ponnada, A.; Manjourides, J. EMA: Microinteraction-based Ecological Momentary Assessment (EMA) Using a Smartwatch. *Proceedings of the ... ACM International Conference on Ubiquitous Computing . UbiComp (Conference)* **2016**, *2016*, 1124–1128. https://doi.org/10.1145/2971648.2971717.
645. Connelly, K.; Stein, K.F.; Chaudry, B.; Trabold, N. Development of an Ecological Momentary Assessment Mobile App for a Low-Literacy, Mexican American Population to Collect Disordered Eating Behaviors. *JMIR Public Health and Surveillance* **2016**, *2*, e31. https://doi.org/10.2196/publichealth.5511.
646. Jones, K.K.; Zenk, S.N.; McDonald, A.; Corte, C. Experiences of African-American Women with Smartphone-Based Ecological Momentary Assessment. *Public Health Nursing* **2016**, *33*, 371–380. https://doi.org/10.1111/phn.12239.
647. Krieke, L.V.D.; Jeronimus, B.F.; Blaauw, F.J.; Wanders, R.B.; Emerencia, A.C.; Schenk, H.M.; Vos, S.D.; Snippe, E.; Wichers, M.; Wigman, J.T.; et al. HowNutsAreTheDutch (HoeGekIsNL): A crowdsourcing study of mental symptoms and strengths. *International Journal of Methods in Psychiatric Research* **2015**, *25*, 123–144. https://doi.org/10.1002/mpr.1495.
648. Bromberg, M.H.; Connelly, M.; Anthony, K.K.; Gil, K.M.; Schanberg, L.E. Prospective Mediation Models of Sleep, Pain and Daily Function in Children with Arthritis using Ecological Momentary Assessment. *The Clinical journal of pain* **2016**, *32*, 471–477. https://doi.org/10.1097/AJP.0000000000000298.
649. Borzekowski, D.L.G.; Chen, J.C. Tobacco cues in India: An ecological momentary assessment. *Tobacco Induced Diseases* **2016**, *14*, 16. https://doi.org/10.1186/s12971-016-0081-z.
650. Huh, J.; Cerrada, C.J.; Kirkpatrick, M.G.; Dunton, G.; Leventhal, A.M. Social contexts of momentary craving to smoke among Korean American emerging adults. *Addictive behaviors* **2016**, *56*, 23–29. https://doi.org/10.1016/j.addbeh.2016.01.006.
651. Kolar, D.R.; Hammerle, F.; Jenetzky, E.; Huss, M.; Bürger, A. Aversive tension in female adolescents with Anorexia Nervosa: a controlled ecological momentary assessment using smartphones. *BMC Psychiatry* **2016**, *16*, 97. https://doi.org/10.1186/s12888-016-0807-8.
652. Depp, C.A.; Moore, R.C.; Perivoliotis, D.; Holden, J.L.; Swendsen, J.; Granholm, E.L. Social behavior, interaction appraisals, and suicidal ideation in schizophrenia: The dangers of being alone. *Schizophrenia research* **2016**, *172*, 195–200. https://doi.org/10.1016/j.schres.2016.02.028.
653. Boh, B.; Lemmens, L.H.J.M.; Jansen, A.; Nederkoorn, C.; Kerkhofs, V.; Spanakis, G.; Weiss, G.; Roefs, A. An Ecological Momentary Intervention for weight loss and healthy eating via smartphone and Internet: study protocol for a randomised controlled trial. *Trials* **2016**, *17*, 154. https://doi.org/10.1186/s13063-016-1280-x.
654. Linas, B.S.; Genz, A.; Westergaard, R.P.; Chang, L.W.; Bollinger, R.C.; Latkin, C.; Kirk, G.D. Ecological Momentary Assessment of Illicit Drug Use Compared to Biological and Self-Reported Methods. *JMIR mHealth and uHealth* **2016**, *4*, e27. https://doi.org/10.2196/mhealth.4470.
655. Depp, C.A.; Moore, R.C.; Dev, S.I.; Mausbach, B.T.; Eyler, L.T.; Granholm, E.L. The temporal course and clinical correlates of subjective impulsivity in bipolar disorder as revealed through ecological momentary assessment. *Journal of affective disorders* **2016**, *193*, 145–150. https://doi.org/10.1016/j.jad.2015.12.016.
656. Broen, M.P.G.; Marsman, V.A.M.; Kuijf, M.L.; Van Oostenbrugge, R.J.; van Os, J.; Leentjens, A.F.G. Unraveling the Relationship between Motor Symptoms, Affective States and Contextual Factors in Parkinson's Disease: A Feasibility Study of the Experience Sampling Method. *PLoS ONE* **2016**, *11*, e0151195. https://doi.org/10.1371/journal.pone.0151195.

657. Asselbergs, J.; Ruwaard, J.; Ejdys, M.; Schrader, N.; Sijbrandij, M.; Riper, H. Mobile Phone-Based Unobtrusive Ecological Momentary Assessment of Day-to-Day Mood: An Explorative Study. *Journal of Medical Internet Research* **2016**, *18*, e72. https://doi.org/10.2196/jmir.5505.
658. Wright, C.J.C.; Dietze, P.M.; Crockett, B.; Lim, M.S.C. Participatory development of MIDY (Mobile Intervention for Drinking in Young people). *BMC Public Health* **2016**, *16*, 184. https://doi.org/10.1186/s12889-016-2876-5.
659. Engelen, L.; Chau, J.Y.; Burks-Young, S.; Bauman, A. Application of ecological momentary assessment in workplace health evaluation. *Health Promotion Journal of Australia* **2016**, *27*, 259–263. https://doi.org/10.1071/HE16043.
660. Schwartz, S.; Schultz, S.; Reider, A.; Saunders, E.F. Daily mood monitoring of symptoms using smartphones in bipolar disorder: A pilot study assessing the feasibility of ecological momentary assessment. *Journal of affective disorders* **2016**, *191*, 88–93. https://doi.org/10.1016/j.jad.2015.11.013.
661. Hendriks, M.; Ludwigs, K.; Veenhoven, R. Why are Locals Happier than Internal Migrants? The Role of Daily Life. *Social Indicators Research* **2016**, *125*, 481–508. https://doi.org/10.1007/s11205-014-0856-7.
662. Gotink, R.A.; Hermans, K.S.; Geschwind, N.; De Nooij, R.; De Groot, W.T.; Speckens, A.E. Mindfulness and mood stimulate each other in an upward spiral: a mindful walking intervention using experience sampling. *Mindfulness* **2016**, *7*, 1114–1122. https://doi.org/10.1007/s12671-016-0550-8.
663. Ramsey, A.T.; Wetherell, J.L.; Depp, C.; Dixon, D.; Lenze, E. Feasibility and Acceptability of Smartphone Assessment in Older Adults with Cognitive and Emotional Difficulties. *Journal of technology in human services* **2016**, *34*, 209–223. https://doi.org/10.1080/15228835.2016.1170649.
664. Dunton, G.F.; Dzubur, E.; Li, M.; Huh, J.; McConnell, R.; Intille, S. Momentary Assessment of Psychosocial Stressors, Context, and Asthma Symptoms in Hispanic Adolescents. *Behavior modification* **2016**, *40*, 257–280. https://doi.org/10.1177/0145445515608145.
665. Pike, J.R.; Xie, B.; Tan, N.; Sabado-Liwag, M.D.; Orne, A.; Toilolo, T.; Cen, S.; May, V.; Lee, C.; Pang, V.K.; et al. Developing an Internet- and Mobile-Based System to Measure Cigarette Use Among Pacific Islanders: An Ecological Momentary Assessment Study. *JMIR mHealth and uHealth* **2016**, *4*, e2. https://doi.org/10.2196/mhealth.4437.
666. Bruening, M.; van Woerden, I.; Todd, M.; Brennhofer, S.; Laska, M.N.; Dunton, G. A Mobile Ecological Momentary Assessment Tool (devilSPARC) for Nutrition and Physical Activity Behaviors in College Students: A Validation Study. *Journal of Medical Internet Research* **2016**, *18*, e209. https://doi.org/10.2196/jmir.5969.
667. Do, B.; Yang, C.H.; Lopez, N.V.; Mason, T.B.; Margolin, G.; Dunton, G.F. Investigating the momentary association between maternal support and children's fruit and vegetable consumption using ecological momentary assessment. *Appetite* **2020**, *150*, 104667. https://doi.org/10.1016/j.appet.2020.104667.
668. Ryan, P.; Brown, R.; Csuka, M.E.; Papanek, P. Efficacy of Osteoporosis Prevention Smartphone App on Bone Mineral Density and Trabecular Bone Scores. *Nursing research* **2020**, *69*, 31–41. https://doi.org/10.1097/NNR.0000000000000392.
669. Vakhrusheva, J.; Khan, S.; Chang, R.; Hansen, M.; Ayanruoh, L.; Gross, J.; Kimhy, D. Lexical Analysis of Emotional Responses to "Real-World" Experiences in Individuals with Schizophrenia. *Schizophrenia research* **2020**, *216*, 272–278. https://doi.org/10.1016/j.schres.2019.11.045.
670. Huhn, A.; Khan, V.; Ketelaar, P.; Van 't Riet, J.; Konig, R.; Rozendaal, E.; Batalas, N.; Markopoulos, P. Does location congruence matter? A field study on the effects of location-based advertising on perceived ad intrusiveness, relevance & value. *COMPUTERS IN HUMAN BEHAVIOR* **2017**, *73*, 659–668. https://doi.org/10.1016/j.chb.2017.03.003.
671. Slot, E.; Vulperhorst, J.; Bronkhorst, L.; van Der Rijst, R.; Wubbels, T.; Akkerman, S. Mechanisms of interest sustainment. *LEARNING CULTURE AND SOCIAL INTERACTION* **2020**, *24*. https://doi.org/10.1016/j.lcsi.2019.100356.
672. Andrews, L.; Russell Bennett, R.; Drennan, J. Capturing affective experiences using the SMS Experience Sampling (SMS-ES) method. *International Journal of Market Research* **2011**, *53*, 479–506. Publisher: Sage Publications Inc.
673. Forgasz, H.J.; Leder, G.C. Academic Life: Monitoring Work Patterns and Daily Activities. *Australian Educational Researcher* **2006**, *33*, 1–22. Publisher: Australian Educational Researcher.
674. Palmer, K.; Ciccarelli, M.; Falkmer, T.; Parsons, R. Associations between exposure to Information and Communication Technology (ICT) and reported discomfort among adolescents. *Work* **2014**, *48*, 165–173. Publisher: IOS Press.
675. Könen, T.; Dirk, J.; Schmiedek, F. Cognitive Benefits of Last Night's Sleep: Daily Variations in Children's Sleep Behavior Are Related to Working Memory Fluctuations. *Journal of Child Psychology and Psychiatry* **2015**, *56*, 171–182. Publisher: Journal of Child Psychology and Psychiatry.
676. Daugherty, D.A.; Runyan, J.D.; Steenbergh, T.A.; Fratzke, B.J.; Fry, B.N.; Westra, E. Smartphone delivery of a hope intervention: Another way to flourish. *PloS one* **2018**, *13*, e0197930. https://doi.org/10.1371/journal.pone.0197930.
677. Pearson, A.D.; Young, C.M.; Shank, F.; Neighbors, C. Flow mediates the relationship between problematic smartphone use and satisfaction with life among college students. *Journal of American college health : J of ACH* **2021**, pp. 1–9. Place: United States, https://doi.org/10.1080/07448481.2021.1910274.
678. Haque, M.M.; Rabbani, M.; Dipal, D.D.; Zarif, M.I.I.; Iqbal, A.; Akhter, S.; Parveen, S.; Rasel, M.; Rabbani, G.; Alam, F.; et al. Grant Report on mCARE: Mobile-Based Care for Children with Autism Spectrum Disorder (ASD) for Low- and Middle-Income Countries (LMICs). *Journal of psychiatry and brain science* **2021**, *6*, e210004. https://doi.org/10.20900/jpbs.20210004.
679. Zenk, S.N.; Horoi, I.; McDonald, A.; Corte, C.; Riley, B.; Odoms-Young, A.M. Ecological momentary assessment of environmental and personal factors and snack food intake in African American women. *Appetite* **2014**, *83*, 333–341. https://doi.org/10.1016/j.appet.2014.09.008.

680. Herbert, M.S.; Wooldridge, J.S.; Paolillo, E.W.; Depp, C.A.; Moore, R.C. Social Contact Frequency and Pain among Older Adults with HIV: An Ecological Momentary Assessment Study. *Annals of behavioral medicine : a publication of the Society of Behavioral Medicine* **2022**, *56*, 168–175. https://doi.org/10.1093/abm/kaab037.
681. Bertz, J.W.; Panlilio, L.V.; Stull, S.W.; Smith, K.E.; Reamer, D.; Holtyn, A.F.; Toegel, F.; Kowalczyk, W.J.; Phillips, K.A.; Epstein, D.H.; et al. Being at work improves stress, craving, and mood for people with opioid use disorder: Ecological momentary assessment during a randomized trial of experimental employment in a contingency-management-based therapeutic workplace. *Behaviour research and therapy* **2022**, *152*, 104071. https://doi.org/10.1016/j.brat.2022.104071.
682. Thrul, J.; Bühler, A.; Ferguson, S.G. An Internet-based ecological momentary assessment study relying on participants' own mobile phones: insights from a study with young adult smokers. *European addiction research* **2015**, *21*, 1–5. Place: Switzerland, https://doi.org/10.1159/000363231.
683. Emodi-Perlman, A.; Manfredini, D.; Shalev, T.; Bracci, A.; Frideman-Rubin, P.; Eli, I. Psychosocial and Behavioral Factors in Awake Bruxism-Self-Report versus Ecological Momentary Assessment. *Journal of clinical medicine* **2021**, *10*. https://doi.org/10.3390/jcm10194447.
684. Roberts, M.E.; Keller-Hamilton, B.; Ferketich, A.K. Testing if attitudes mediate the association between advertising exposure and adolescent tobacco use. *Addictive behaviors* **2022**, *134*, 107415. Place: England, https://doi.org/10.1016/j.addbeh.2022.107415.
685. Thompson, W.K.; Gershon, A.; O'Hara, R.; Bernert, R.A.; Depp, C.A. The prediction of study-emergent suicidal ideation in bipolar disorder: a pilot study using ecological momentary assessment data. *Bipolar disorders* **2014**, *16*, 669–677. https://doi.org/10.1111/bdi.12218.
686. Smiley, S.L.; Elmasry, H.; Webb Hooper, M.; Niaura, R.S.; Hamilton, A.B.; Milburn, N.G. Feasibility of Ecological Momentary Assessment of Daily Sexting and Substance Use Among Young Adult African American Gay and Bisexual Men: A Pilot Study. *JMIR research protocols* **2017**, *6*, e9. https://doi.org/10.2196/resprot.6520.
687. Kuntsche, E.; Labhart, F. ICAT: Development of an Internet-Based Data Collection Method for Ecological Momentary Assessment Using Personal Cell Phones. *European journal of psychological assessment : official organ of the European Association of Psychological Assessment* **2013**, *29*, 140–148. https://doi.org/10.1027/1015-5759/a000137.
688. Goldstein, S.P.; Brick, L.A.; Thomas, J.G.; Forman, E.M. Examination of the relationship between lapses and weight loss in a smartphone-based just-in time adaptive intervention. *Translational behavioral medicine* **2021**, *11*, 993–1005. Place: England, https://doi.org/10.1093/tbm/ibaa097.
689. Brenner, C.J.; Ben-Zeev, D. Affective forecasting in schizophrenia: comparing predictions to real-time Ecological Momentary Assessment (EMA) ratings. *Psychiatric rehabilitation journal* **2014**, *37*, 316–320. Place: United States, https://doi.org/10.1037/prj0000105.
690. Abrantes, A.M.; Kunicki, Z.; Braun, T.; Miranda, R.J.; Blevins, C.E.; Brick, L.; Thomas, G.; Marsh, E.; Feltus, S.; Stein, M.D. Daily associations between alcohol and sweets craving and consumption in early AUD recovery: Results from an ecological momentary assessment study. *Journal of substance abuse treatment* **2022**, *132*, 108614. Place: United States, https://doi.org/10.1016/j.jsat.2021.108614.
691. Martinez, G.J.; Mattingly, S.M.; Robles-Granda, P.; Saha, K.; Sirigiri, A.; Young, J.; Chawla, N.; De Choudhury, M.; D'Mello, S.; Mark, G.; et al. Predicting Participant Compliance With Fitness Tracker Wearing and Ecological Momentary Assessment Protocols in Information Workers: Observational Study. *JMIR mHealth and uHealth* **2021**, *9*, e22218. https://doi.org/10.2196/22218.
692. Thøgersen-Ntoumani, C.; Loughren, E.A.; Kinnafick, F.E.; Taylor, I.M.; Duda, J.L.; Fox, K.R. Changes in work affect in response to lunchtime walking in previously physically inactive employees: A randomized trial. *Scandinavian journal of medicine & science in sports* **2015**, *25*, 778–787. Place: Denmark, https://doi.org/10.1111/sms.12398.
693. Comulada, W.S. Mobile Phone Assessment in Egocentric Networks: A Pilot Study on Gay Men and Their Peers. *Connections (Toronto, Ont.)* **2014**, *34*, 43–51. https://doi.org/10.17266/34.1.4.
694. Comulada, W.S.; Lightfoot, M.; Swendeman, D.; Grella, C.; Wu, N. Compliance to Cell Phone-Based EMA Among Latino Youth in Outpatient Treatment. *Journal of ethnicity in substance abuse* **2015**, *14*, 232–250. https://doi.org/10.1080/15332640.2014.986354.
695. Ben-Zeev, D.; Frounfelker, R.; Morris, S.B.; Corrigan, P.W. Predictors of Self-Stigma in Schizophrenia: New Insights Using Mobile Technologies. *Journal of dual diagnosis* **2012**, *8*, 305–314. https://doi.org/10.1080/15504263.2012.723311.
696. Huguet, A.; McGrath, P.J.; Wheaton, M.; Mackinnon, S.P.; Rozario, S.; Tougas, M.E.; Stinson, J.N.; MacLean, C. Testing the Feasibility and Psychometric Properties of a Mobile Diary (myWHI) in Adolescents and Young Adults With Headaches. *JMIR mHealth and uHealth* **2015**, *3*, e39. https://doi.org/10.2196/mhealth.3879.
697. Naya, C.H.; Chu, D.; Wang, W.L.; Nicolo, M.; Dunton, G.F.; Mason, T.B. Children's Daily Negative Affect Patterns and Food Consumption on Weekends: An Ecological Momentary Assessment Study. *Journal of nutrition education and behavior* **2022**, *54*, 600–609. Place: United States Publisher: Elsevier, https://doi.org/10.1016/j.jneb.2022.02.007.
698. Bauer, J.M.; Nielsen, K.S.; Hofmann, W.; Reisch, L.A. Healthy eating in the wild: An experience-sampling study of how food environments and situational factors shape out-of-home dietary success. *Social science & medicine (1982)* **2022**, *299*, 114869. Place: England Publisher: Pergamon, https://doi.org/10.1016/j.socscimed.2022.114869.
699. Rich, M.; Bickham, D.S.; Shrier, L.A. Measuring Youth Media Exposure. *American Behavioral Scientist* **2015**, *59*, 1736–1754.
700. Turner, A.; Brokamp, C.; Wolfe, C.; Reponen, T.; Ryan, P. Personal exposure to average weekly ultrafine particles, lung function, and respiratory symptoms in asthmatic and non-asthmatic adolescents. *Environment International* **2021**, *156*, N.PAG–N.PAG.

701. Ketonen, E.E.; Malmberg, L.E.; Salmela-Aro, K.; Muukkonen, H.; Tuominen, H.; Lonka, K. The role of study engagement in university students' daily experiences: A multilevel test of moderation. *Learning & Individual Differences* **2019**, *69*, 196–205.
702. Tomlinson, M.; Rotheram-Borus, M.J.; Doherty, T.; Swendeman, D.; Tsai, A.C.; Ijumba, P.; le Roux, I.; Jackson, D.; Stewart, J.; Friedman, A.; et al. Value of a mobile information system to improve quality of care by community health workers. *South African Journal of Information Management* **2013**, *15*, 1–9.
703. Hanssen, E.; Balvert, S.; Oorschot, M.; Borkelmans, K.; van Os, J.; Delespaul, P.; Fett, A.K. An ecological momentary intervention incorporating personalised feedback to improve symptoms and social functioning in schizophrenia spectrum disorders. *Psychiatry research* **2020**, *284*, 112695. Place: Ireland Publisher: Elsevier/North-Holland Biomedical Press, https://doi.org/10.1016/j.psychres.2019.112695.
704. Andersson, K.E.; Andersen, L.S.; Christensen, J.H.; Neher, T. Assessing Real-Life Benefit From Hearing-Aid Noise Management: SSQ12 Questionnaire Versus Ecological Momentary Assessment With Acoustic Data-Logging. *American journal of audiology* **2021**, *30*, 93–104. Place: United States Publisher: American Speech-Language-Hearing Association, https://doi.org/10.1044/2020_AJA-20-00042.
705. Litmanen, T.; Lonka, K.; Inkinen, M.; Lipponen, L.; Hakkarainen, K. Capturing Teacher Students' Emotional Experiences in Context: Does Inquiry-Based Learning Make a Difference? *Instructional Science: An International Journal of the Learning Sciences* **2012**, *40*, 1083–1101. Publisher: Instructional Science: An International Journal of the Learning Sciences.
706. Khor, A.S.; Melvin, G.A.; Reid, S.C.; Gray, K.M. Coping, Daily Hassles and Behavior and Emotional Problems in Adolescents with High-Functioning Autism/Asperger's Disorder. *Journal of Autism and Developmental Disorders* **2014**, *44*, 593–608. Publisher: Journal of Autism and Developmental Disorders.
707. Schinkel-Bielefeld, N.; Kunz, P.; Zutz, A.; Buder, B. Evaluation of Hearing Aids in Everyday Life Using Ecological Momentary Assessment: What Situations Are We Missing? *American journal of audiology* **2020**, *29*, 591–609. Place: United States Publisher: American Speech-Language-Hearing Association, https://doi.org/10.1044/2020_AJA-19-00075.
708. EXPLORING THE MOMENT OF CONSUMPTION USING CELL PHONES. *Journal of Electronic Commerce Research* **2005**, *6*, 225–240.
709. Pronk, M.; Besser, J.; Smits, C.; Feenstra-Kikken, V.; van Beek, H.; Polleunis, C.; Kramer, S.E. Rationale, Theoretical Underpinnings, and Design of HEAR-aware: Providing Adults With Hearing Loss With Tailored Support to Self-Manage Their Hearing Problems via a Smartphone App, as an Alternative to Hearing Aids. *American journal of audiology* **2020**, *29*, 648–660. Place: United States Publisher: American Speech-Language-Hearing Association, https://doi.org/10.1044/2020_AJA-19-00079.
710. Dennis, M.L.; Scott, C.K.; Funk, R.R.; Nicholson, L. A Pilot Study to Examine the Feasibility and Potential Effectiveness of Using Smartphones to Provide Recovery Support for Adolescents. *Substance abuse* **2015**, *36*, 486–492. Place: United States Publisher: Routledge, https://doi.org/10.1080/08897077.2014.970323.
711. McKee, H.C.; Ntoumanis, N.; Taylor, I.M. An ecological momentary assessment of lapse occurrences in dieters. *Annals of behavioral medicine : a publication of the Society of Behavioral Medicine* **2014**, *48*, 300–310. Place: England Publisher: Oxford University Press, https://doi.org/10.1007/s12160-014-9594-y.
712. Reynolds, G.L.; Fisher, D.G.; Laurenceau, J.P.; Fortenberry, J.D. An Electronic Daily Diary Study of Anal Intercourse in Drug-Using Women. *AIDS and behavior* **2015**, *19*, 2325–2332. Place: United States Publisher: Springer Science + Business Media, https://doi.org/10.1007/s10461-015-1045-7.
713. Depp, C.A.; Ceglowski, J.; Wang, V.C.; Yaghouti, F.; Mausbach, B.T.; Thompson, W.K.; Granholm, E.L. Augmenting psychoeducation with a mobile intervention for bipolar disorder: a randomized controlled trial. *Journal of affective disorders* **2015**, *174*, 23–30. Place: Netherlands Publisher: Elsevier/North-Holland Biomedical Press, https://doi.org/10.1016/j.jad.2014.10.053.
714. Linas, B.S.; Latkin, C.; Westergaard, R.P.; Chang, L.W.; Bollinger, R.C.; Genz, A.; Kirk, G.D. Capturing illicit drug use where and when it happens: an ecological momentary assessment of the social, physical and activity environment of using versus craving illicit drugs. *Addiction (Abingdon, England)* **2015**, *110*, 315–325. Place: England Publisher: Wiley-Blackwell, https://doi.org/10.1111/add.12768.
715. Berkman, E.T.; Giuliani, N.R.; Pruitt, A.K. Comparison of text messaging and paper-and-pencil for ecological momentary assessment of food craving and intake. *Appetite* **2014**, *81*, 131–137. Place: England Publisher: Academic Press, https://doi.org/10.1016/j.appet.2014.06.010.
716. Fatseas, M.; Serre, F.; Alexandre, J.M.; Debrabant, R.; Auriacombe, M.; Swendsen, J. Craving and substance use among patients with alcohol, tobacco, cannabis or heroin addiction: a comparison of substance- and person-specific cues. *Addiction (Abingdon, England)* **2015**, *110*, 1035–1042. Place: England Publisher: Wiley-Blackwell, https://doi.org/10.1111/add.12882.
717. Ma, P.; Kendzor, D.E.; Poonawalla, I.B.; Balis, D.S.; Businelle, M.S. Daily nicotine patch wear time predicts smoking abstinence in socioeconomically disadvantaged adults: An analysis of ecological momentary assessment data. *Drug and alcohol dependence* **2016**, *169*, 64–67. Place: Ireland Publisher: Elsevier, https://doi.org/10.1016/j.drugalcdep.2016.10.013.
718. Stinson, J.N.; Jibb, L.A.; Nguyen1, C.; Nathan, P.C.; Maloney, A.M.; Dupuis, L.L.; Gerstle, J.T.; Alman, B.; Hopyan, S.; Strahlendorf, C.; et al. Development and Testing of a Multidimensional iPhone Pain Assessment Application for Adolescents with Cancer. *Journal of Medical Internet Research* **2013**, *15*, 1–1.
719. Luczak, S.E.; Rosen, I.G.; Wall, T.L. Development of a real-time repeated-measures assessment protocol to capture change over the course of a drinking episode. *Alcohol and alcoholism (Oxford, Oxfordshire)* **2015**, *50*, 180–187. Place: England Publisher: Oxford University Press, https://doi.org/10.1093/alcalc/agu100.

720. Hofmann, W.; Adriaanse, M.; Vohs, K.D.; Baumeister, R.F. Dieting and the self-control of eating in everyday environments: an experience sampling study. *British journal of health psychology* **2014**, *19*, 523–539. Place: England Publisher: Wiley-Blackwell, https://doi.org/10.1111/bjhp.12053.
721. Garcia-Palacios, A.; Herrero, R.; Belmonte, M.A.; Castilla, D.; Guixeres, J.; Molinari, G.; Baños, R.M. Ecological momentary assessment for chronic pain in fibromyalgia using a smartphone: a randomized crossover study. *European journal of pain (London, England)* **2014**, *18*, 862–872. Place: England Publisher: Wiley, https://doi.org/10.1002/j.1532-2149.2013.00425.x.
722. Wilson, M.B.; Kallogjeri, D.; Joplin, C.N.; Gorman, M.D.; Krings, J.G.; Lenze, E.J.; Nicklaus, J.E.; Spitznagel, Jr, E.E.; Piccirillo, J.F. Ecological momentary assessment of tinnitus using smartphone technology: a pilot study. *Otolaryngology–head and neck surgery : official journal of American Academy of Otolaryngology-Head and Neck Surgery* **2015**, *152*, 897–903. Place: England Publisher: Sage, https://doi.org/10.1177/0194599815569692.
723. Blödt, S.; Pach, D.; Roll, S.; Witt, C.M. Effectiveness of app-based relaxation for patients with chronic low back pain (Relaxback) and chronic neck pain (Relaxneck): study protocol for two randomized pragmatic trials. *Trials* **2014**, *15*, 490. Place: England Publisher: BioMed Central, https://doi.org/10.1186/1745-6215-15-490.
724. Brodbeck, J.; Bachmann, M.S.; Brown, A.; Znoj, H.J. Effects of depressive symptoms on antecedents of lapses during a smoking cessation attempt: an ecological momentary assessment study. *Addiction (Abingdon, England)* **2014**, *109*, 1363–1370. Place: England Publisher: Wiley-Blackwell, https://doi.org/10.1111/add.12563.
725. Kovac, M.; Mosner, M.; Miller, S.; Hanna, E.K.; Dichter, G.S. Experience Sampling of Positive Affect in Adolescents with Autism: Feasibility and Preliminary Findings. *Research in autism spectrum disorders* **2016**, *29-30*, 57–65. Place: Netherlands Publisher: Elsevier, https://doi.org/10.1016/j.rasd.2016.06.003.
726. Wenze, S.J.; Armey, M.F.; Miller, I.W. Feasibility and Acceptability of a Mobile Intervention to Improve Treatment Adherence in Bipolar Disorder: A Pilot Study. *Behavior modification* **2014**, *38*, 497–515. Place: United States Publisher: Sage Publications, https://doi.org/10.1177/0145445513518421.
727. Yang, C.; Linas, B.; Kirk, G.; Bollinger, R.; Chang, L.; Chander, G.; Siconolfi, D.; Braxton, S.; Rudolph, A.; Latkin, C. Feasibility and Acceptability of Smartphone-Based Ecological Momentary Assessment of Alcohol Use Among African American Men Who Have Sex With Men in Baltimore. *JMIR mHealth and uHealth* **2015**, *3*, e67. Place: Canada Publisher: JMIR Publications Inc, https://doi.org/10.2196/mhealth.4344.
728. Husky, M.; Olié, E.; Guillaume, S.; Genty, C.; Swendsen, J.; Courtet, P. Feasibility and validity of ecological momentary assessment in the investigation of suicide risk. *Psychiatry research* **2014**, *220*, 564–570. Place: Ireland Publisher: Elsevier/North-Holland Biomedical Press, https://doi.org/10.1016/j.psychres.2014.08.019.
729. Mundi, M.S.; Lorentz, P.A.; Grothe, K.; Kellogg, T.A.; Collazo-Clavell, M.L. Feasibility of Smartphone-Based Education Modules and Ecological Momentary Assessment/Intervention in Pre-bariatric Surgery Patients. *Obesity surgery* **2015**, *25*, 1875–1881. Place: United States Publisher: Springer Science + Business Media, https://doi.org/10.1007/s11695-015-1617-7.
730. Gidlow, C.J.; Randall, J.; Gillman, J.; Silk, S.; Jones, M.V. Hair cortisol and self-reported stress in healthy, working adults. *Psychoneuroendocrinology* **2016**, *63*, 163–169. Place: England Publisher: Pergamon Press, https://doi.org/10.1016/j.psyneuen.2015.09.022.
731. Fernández-Castro, J.; Martínez-Zaragoza, F.; Rovira, T.; Edo, S.; Solanes-Puchol, ; Martín-Del-Río, B.; García-Sierra, R.; Benavides-Gil, G.; Doval, E. How does emotional exhaustion influence work stress? Relationships between stressor appraisals, hedonic tone, and fatigue in nurses' daily tasks: A longitudinal cohort study. *International journal of nursing studies* **2017**, *75*, 43–50. Place: England Publisher: Pergamon Press, https://doi.org/10.1016/j.ijnurstu.2017.07.002.
732. Monk, R.L.; Heim, D.; Qureshi, A.; Price, A. "I have no clue what I drunk last night" using Smartphone technology to compare in-vivo and retrospective self-reports of alcohol consumption. *PloS one* **2015**, *10*, e0126209. Place: United States Publisher: Public Library of Science, https://doi.org/10.1371/journal.pone.0126209.
733. Hallensleben, N.; Spangenberg, L.; Forkmann, T.; Rath, D.; Hegerl, U.; Kersting, A.; Kallert, T.W.; Glaesmer, H. Investigating the Dynamics of Suicidal Ideation. *Crisis* **2018**, *39*, 65–69. Place: Canada Publisher: Hogrefe, https://doi.org/10.1027/0227-5910/a000464.
734. Shrier, L.A.; Spalding, A. "Just Take a Moment and Breathe and Think": Young Women with Depression Talk about the Development of an Ecological Momentary Intervention to Reduce Their Sexual Risk. *Journal of pediatric and adolescent gynecology* **2017**, *30*, 116–122. Place: United States Publisher: Elsevier Science, https://doi.org/10.1016/j.jpag.2016.08.009.
735. Bickham, D.S.; Hswen, Y.; Rich, M. Media use and depression: exposure, household rules, and symptoms among young adolescents in the USA. *International journal of public health* **2015**, *60*, 147–155. Place: Switzerland Publisher: Frontiers Media S.A, https://doi.org/10.1007/s00038-014-0647-6.
736. Forman, E.M.; Shaw, J.A.; Goldstein, S.P.; Butryn, M.L.; Martin, L.M.; Meiran, N.; Crosby, R.D.; Manasse, S.M. Mindful decision making and inhibitory control training as complementary means to decrease snack consumption. *Appetite* **2016**, *103*, 176–183. Place: England Publisher: Academic Press, https://doi.org/10.1016/j.appet.2016.04.014.
737. Huh, J.; Shin, H.; Leventhal, A.M.; Spruijt-Metz, D.; Abramova, Z.; Cerrada, C.; Hedeker, D.; Dunton, G. Momentary negative moods and being with friends precede cigarette use among Korean American emerging adults. *Nicotine & tobacco research : official journal of the Society for Research on Nicotine and Tobacco* **2014**, *16*, 1248–1254. Place: England Publisher: Oxford University Press, https://doi.org/10.1093/ntr/ntu063.

738. Droit-Volet, S.; Trahanias, P.; Maniadakis, M. Passage of time judgments in everyday life are not related to duration judgments except for long durations of several minutes. *Acta psychologica* **2017**, *173*, 116–121. Place: Netherlands Publisher: North Holland Publishing, https://doi.org/10.1016/j.actpsy.2016.12.010.
739. Juengst, S.B.; Graham, K.M.; Pulantara, I.W.; McCue, M.; Whyte, E.M.; Dicianno, B.E.; Parmanto, B.; Arenth, P.M.; Skidmore, E.R.D.; Wagner, A.K. Pilot feasibility of an mHealth system for conducting ecological momentary assessment of mood-related symptoms following traumatic brain injury. *Brain injury* **2015**, *29*, 1351–1361. Place: England Publisher: Informa Healthcare, https://doi.org/10.3109/02699052.2015.1045031.
740. Bandiera, F.C.; Atem, F.; Ma, P.; Businelle, M.S.; Kendzor, D.E. Post-quit stress mediates the relation between social support and smoking cessation among socioeconomically disadvantaged adults. *Drug and alcohol dependence* **2016**, *163*, 71–76. Place: Ireland Publisher: Elsevier, https://doi.org/10.1016/j.drugalcdep.2016.03.023.
741. Dedert, E.A.; Hicks, T.A.; Dennis, P.A.; Calhoun, P.S.; Beckham, J.C. Roles of inter-individual differences and intra-individual acute elevations in early smoking lapse in people with posttraumatic stress disorder. *Addictive behaviors* **2016**, *60*, 171–176. Place: England Publisher: Elsevier Science, https://doi.org/10.1016/j.addbeh.2016.04.007.
742. Hung, S.; Li, M.S.; Chen, Y.L.; Chiang, J.H.; Chen, Y.Y.; Hung, G.C.L. Smartphone-based ecological momentary assessment for Chinese patients with depression: An exploratory study in Taiwan. *Asian journal of psychiatry* **2016**, *23*, 131–136. Place: Netherlands Publisher: Elsevier, https://doi.org/10.1016/j.ajp.2016.08.003.
743. Perry, T.T.; Marshall, A.; Berlinski, A.; Rettiganti, M.; Brown, R.H.; Randle, S.M.; Luo, C.; Bian, J. Smartphone-based vs paper-based asthma action plans for adolescents. *Annals of allergy, asthma & immunology : official publication of the American College of Allergy, Asthma, & Immunology* **2017**, *118*, 298–303. Place: United States Publisher: American College of Allergy, Asthma, and Immunology, https://doi.org/10.1016/j.anai.2016.11.028.
744. Furnari, M.; Epstein, D.H.; Phillips, K.A.; Jobes, M.L.; Kowalczyk, W.J.; Vahabzadeh, M.; Lin, J.L.; Preston, K.L. Some of the people, some of the time: field evidence for associations and dissociations between stress and drug use. *Psychopharmacology* **2015**, *232*, 3529–3537. Place: Germany Publisher: Springer-Verlag, https://doi.org/10.1007/s00213-015-3998-7.
745. Jasik, C.B.; Berna, M.; Martin, M.; Ozer, E.M. Teen Preferences for Clinic-Based Behavior Screens: Who, Where, When, and How? *The Journal of adolescent health : official publication of the Society for Adolescent Medicine* **2016**, *59*, 722–724. Place: United States Publisher: Elsevier, https://doi.org/10.1016/j.jadohealth.2016.08.009.
746. Brookie, K.L.; Mainvil, L.A.; Carr, A.C.; Vissers, M.C.M.; Conner, T.S. The development and effectiveness of an ecological momentary intervention to increase daily fruit and vegetable consumption in low-consuming young adults. *Appetite* **2017**, *108*, 32–41. Place: England Publisher: Academic Press, https://doi.org/10.1016/j.appet.2016.09.015.
747. Könen, T.; Dirk, J.; Leonhardt, A.; Schmiedek, F. The interplay between sleep behavior and affect in elementary school children's daily life. *Journal of experimental child psychology* **2016**, *150*, 1–15. Place: United States Publisher: Academic Press, https://doi.org/10.1016/j.jecp.2016.04.003.
748. Greene, T.; Gelkopf, M.; Grinapol, S.; Werbeloff, N.; Carlson, E.; Lapid, L. Trajectories of traumatic stress symptoms during conflict: A latent class growth analysis. *Journal of affective disorders* **2017**, *220*, 24–30. Place: Netherlands Publisher: Elsevier/North-Holland Biomedical Press, https://doi.org/10.1016/j.jad.2017.05.036.
749. Soong, A.; Chen, J.C.; Borzekowski, D.L. Using Ecological Momentary Assessment to Study Tobacco Behavior in Urban India: There's an App for That. *JMIR research protocols* **2015**, *4*, e76. Place: Canada Publisher: JMIR Publications, https://doi.org/10.2196/resprot.4408.
750. Linas, B.S.; Latkin, C.; Genz, A.; Westergaard, R.P.; Chang, L.W.; Bollinger, R.C.; Kirk, G.D. Utilizing mHealth methods to identify patterns of high risk illicit drug use. *Drug and alcohol dependence* **2015**, *151*, 250–257. Place: Ireland Publisher: Elsevier, https://doi.org/10.1016/j.drugalcdep.2015.03.031.
751. Ainsworth, J.; Palmier-Claus, J.E.; Machin, M.; Barrowclough, C.; Dunn, G.; Rogers, A.; Buchan, I.; Barkus, E.; Kapur, S.; Wykes, T.; et al. A comparison of two delivery modalities of a mobile phone-based assessment for serious mental illness: native smartphone application vs text-messaging only implementations. *Journal of medical Internet research* **2013**, *15*, e60. Place: Canada Publisher: JMIR Publications, https://doi.org/10.2196/jmir.2328.
752. Wetter, D.W.; McClure, J.B.; Cofta-Woerpel, L.; Costello, T.J.; Reitzel, L.R.; Businelle, M.S.; Cinciripini, P.M. A randomized clinical trial of a palmtop computer-delivered treatment for smoking relapse prevention among women. *Psychology of addictive behaviors : journal of the Society of Psychologists in Addictive Behaviors* **2011**, *25*, 365–371. Place: United States Publisher: American Psychological Association, https://doi.org/10.1037/a0022797.
753. Bernhardt, J.M.; Usdan, S.; Mays, D.; Martin, R.; Cremeens, J.; Arriola, K.J. Alcohol assessment among college students using wireless mobile technology. *Journal of studies on alcohol and drugs* **2009**, *70*, 771–775. Place: United States Publisher: Alcohol Research Documentation, Inc., Rutgers, The State University of New Jersey, https://doi.org/10.15288/jsad.2009.70.771.
754. Weaver, A.; Young, A.M.; Rowntree, J.; Townsend, N.; Pearson, S.; Smith, J.; Gibson, O.; Cobern, W.; Larsen, M.; Tarassenko, L. Application of mobile phone technology for managing chemotherapy-associated side-effects. *Annals of oncology : official journal of the European Society for Medical Oncology* **2007**, *18*, 1887–1892. Place: England Publisher: Elsevier, https://doi.org/10.1093/annonc/mdm354.
755. Mulvaney, S.A.; Ho, Y.X.; Cala, C.M.; Chen, Q.; Nian, H.; Patterson, B.L.; Johnson, K.B. Assessing adolescent asthma symptoms and adherence using mobile phones. *Journal of medical Internet research* **2013**, *15*, e141. Place: Canada Publisher: JMIR Publications, https://doi.org/10.2196/jmir.2413.

756. Dunton, G.F.; Kawabata, K.; Intille, S.; Wolch, J.; Pentz, M.A. Assessing the social and physical contexts of children's leisure-time physical activity: an ecological momentary assessment study. *American journal of health promotion : AJHP* **2012**, *26*, 135–142. Place: United States Publisher: SAGE Publishing, https://doi.org/10.4278/ajhp.100211-QUAN-43.
757. Moore, E.; Williams, A.; Bell, I.; Thomas, N. Client experiences of blending a coping-focused therapy for auditory verbal hallucinations with smartphone-based ecological momentary assessment and intervention. *Internet interventions* **2019**, *19*, 100299. Place: Netherlands Publisher: Elsevier B.V, https://doi.org/10.1016/j.invent.2019.100299.
758. Christie, A.; Dagfinrud, H.; Dale, ; Schulz, T.; Hagen, K.B. Collection of patient-reported outcomes;–text messages on mobile phones provide valid scores and high response rates. *BMC medical research methodology* **2014**, *14*, 52. Place: England Publisher: BioMed Central, https://doi.org/10.1186/1471-2288-14-52.
759. Schüz, N.; Walters, J.A.E.; Frandsen, M.; Bower, J.; Ferguson, S.G. Compliance with an EMA monitoring protocol and its relationship with participant and smoking characteristics. *Nicotine & tobacco research : official journal of the Society for Research on Nicotine and Tobacco* **2014**, *16 Suppl 2*, S88–S92. Place: England Publisher: Oxford University Press, https://doi.org/10.1093/ntr/ntt142.
760. Jean, F.A.M.; Swendsen, J.D.; Sibon, I.; Fehér, K.; Husky, M. Daily life behaviors and depression risk following stroke: a preliminary study using ecological momentary assessment. *Journal of geriatric psychiatry and neurology* **2013**, *26*, 138–143. Place: United States Publisher: Sage Publications, https://doi.org/10.1177/0891988713484193.
761. So, S.H.w.; Peters, E.R.; Swendsen, J.; Garety, P.A.; Kapur, S. Detecting improvements in acute psychotic symptoms using experience sampling methodology. *Psychiatry research* **2013**, *210*, 82–88. Place: Ireland Publisher: Elsevier/North-Holland Biomedical Press, https://doi.org/10.1016/j.psychres.2013.05.010.
762. Daniëls, N.E.M.; Bartels, S.L.; Verhagen, S.J.W.; Van Knippenberg, R.J.M.; De Vugt, M.E.; Delespaul, P.A.E.G. Digital assessment of working memory and processing speed in everyday life: Feasibility, validation, and lessons-learned. *Internet interventions* **2019**, *19*, 100300. Place: Netherlands Publisher: Elsevier B.V, https://doi.org/10.1016/j.invent.2019.100300.
763. Brodbeck, J.; Bachmann, M.S.; Znoj, H. Distinct coping strategies differentially predict urge levels and lapses in a smoking cessation attempt. *Addictive behaviors* **2013**, *38*, 2224–2229. Place: England Publisher: Elsevier Science, https://doi.org/10.1016/j.addbeh.2013.02.001.
764. Wolpin, S.; Nguyen, H.Q.; Donesky-Cuenco, D.; Carrieri-Kohlman, V.; Doorenbos, A. Effects of automated prompts for logging symptom and exercise data on mobile devices in patients with chronic obstructive pulmonary disease. *Computers, informatics, nursing : CIN* **2011**, *29*, TC3–TC8. Place: United States Publisher: Lippincott Williams & Wilkins, https://doi.org/10.1097/NCN.0b013e3182155203.
765. Kimhy, D.; Vakhrusheva, J.; Khan, S.; Chang, R.W.; Hansen, M.C.; Ballon, J.S.; Malaspina, D.; Gross, J.J. Emotional granularity and social functioning in individuals with schizophrenia: an experience sampling study. *Journal of psychiatric research* **2014**, *53*, 141–148. Place: England Publisher: Pergamon Press, https://doi.org/10.1016/j.jpsychires.2014.01.020.
766. Smith, B.; Harms, W.D.; Burres, S.; Korda, H.; Rosen, H.; Davis, J. Enhancing behavioral health treatment and crisis management through mobile ecological momentary assessment and SMS messaging. *Health informatics journal* **2012**, *18*, 294–308. Place: England Publisher: SAGE Publications, https://doi.org/10.1177/1460458212445349.
767. Shadel, W.G.; Martino, S.C.; Setodji, C.; Scharf, D. Exposure to pro-smoking media in college students: does type of media channel differentially contribute to smoking risk? *Annals of behavioral medicine : a publication of the Society of Behavioral Medicine* **2013**, *45*, 387–392. Place: England Publisher: Oxford University Press, https://doi.org/10.1007/s12160-012-9461-7.
768. Khor, A.S.; Gray, K.M.; Reid, S.C.; Melvin, G.A. Feasibility and validity of ecological momentary assessment in adolescents with high-functioning autism and Asperger's disorder. *Journal of adolescence* **2014**, *37*, 37–46. Place: England Publisher: Elsevier, https://doi.org/10.1016/j.adolescence.2013.10.005.
769. Mazure, C.M.; Weinberger, A.H.; Pittman, B.; Sibon, I.; Swendsen, J. Gender and stress in predicting depressive symptoms following stroke. *Cerebrovascular diseases (Basel, Switzerland)* **2014**, *38*, 240–246. Place: Switzerland Publisher: Karger, https://doi.org/10.1159/000365838.
770. Baggott, C.; Gibson, F.; Coll, B.; Kletter, R.; Zeltzer, P.; Miaskowski, C. Initial evaluation of an electronic symptom diary for adolescents with cancer. *JMIR research protocols* **2012**, *1*, e23. Place: Canada Publisher: JMIR Publications, https://doi.org/10.2196/resprot.2175.
771. Palmier-Claus, J.E.; Rogers, A.; Ainsworth, J.; Machin, M.; Barrowclough, C.; Laverty, L.; Barkus, E.; Kapur, S.; Wykes, T.; Lewis, S.W. Integrating mobile-phone based assessment for psychosis into people's everyday lives and clinical care: a qualitative study. *BMC psychiatry* **2013**, *13*, 34. Place: England Publisher: BioMed Central, https://doi.org/10.1186/1471-244X-13-34.
772. Dunton, G.F.; Intille, S.S.; Wolch, J.; Pentz, M.A. Investigating the impact of a smart growth community on the contexts of children's physical activity using Ecological Momentary Assessment. *Health & place* **2012**, *18*, 76–84. Place: England Publisher: Elsevier, https://doi.org/10.1016/j.healthplace.2011.07.007.
773. Ribu, L.; Holmen, H.; Torbjørnsen, A.; Wahl, A.K.; Grøttland, A.; Småstuen, M.C.; Elind, E.; Bergmo, T.S.; Breivik, E.; Arsand, E. Low-intensity self-management intervention for persons with type 2 diabetes using a mobile phone-based diabetes diary, with and without health counseling and motivational interviewing: protocol for a randomized controlled trial. *JMIR research protocols* **2013**, *2*, e34. Place: Canada Publisher: JMIR Publications, https://doi.org/10.2196/resprot.2768.

774. Lange, S.; Süß, H.M. Measuring slips and lapses when they occur - ambulatory assessment in application to cognitive failures. *Consciousness and cognition* **2014**, *24*, 1–11. Place: United States Publisher: Academic Press, https://doi.org/10.1016/j.concog.2013.12.008.
775. Sorbi, M.J.; Mak, S.B.; Houtveen, J.H.; Kleiboer, A.M.; van Doornen, L.J.P. Mobile Web-based monitoring and coaching: feasibility in chronic migraine. *Journal of medical Internet research* **2007**, *9*, e38. Place: Canada Publisher: JMIR Publications, https://doi.org/10.2196/jmir.9.5.e38.
776. Dunton, G.F.; Liao, Y.; Kawabata, K.; Intille, S. Momentary assessment of adults' physical activity and sedentary behavior: feasibility and validity. *Frontiers in psychology* **2012**, *3*, 260. Place: Switzerland Publisher: Frontiers Research Foundation, https://doi.org/10.3389/fpsyg.2012.00260.
777. Spook, J.E.; Paulussen, T.; Kok, G.; Van Empelen, P. Monitoring dietary intake and physical activity electronically: feasibility, usability, and ecological validity of a mobile-based Ecological Momentary Assessment tool. *Journal of medical Internet research* **2013**, *15*, e214. Place: Canada Publisher: JMIR Publications, https://doi.org/10.2196/jmir.2617.
778. Tabak, M.; op den Akker, H.; Hermens, H. Motivational cues as real-time feedback for changing daily activity behavior of patients with COPD. *Patient education and counseling* **2014**, *94*, 372–378. Place: Ireland Publisher: Elsevier, https://doi.org/10.1016/j.pec.2013.10.014.
779. Dunton, G.F.; Liao, Y.; Intille, S.; Wolch, J.; Pentz, M.A. Physical and social contextual influences on children's leisure-time physical activity: an ecological momentary assessment study. *Journal of physical activity & health* **2011**, *8 Suppl 1*, S103–S108. Place: United States Publisher: Human Kinetics Publishers, https://doi.org/10.1123/jpah.8.s1.s103.
780. Houtveen, J.H.; Sorbi, M.J. Prodromal functioning of migraine patients relative to their interictal state–an ecological momentary assessment study. *PloS one* **2013**, *8*, e72827. Place: United States Publisher: Public Library of Science, https://doi.org/10.1371/journal.pone.0072827.
781. Cohn, A.M.; Hunter-Reel, D.; Hagman, B.T.; Mitchell, J. Promoting behavior change from alcohol use through mobile technology: the future of ecological momentary assessment. *Alcoholism, clinical and experimental research* **2011**, *35*, 2209–2215. Place: England Publisher: Wiley-Blackwell, https://doi.org/10.1111/j.1530-0277.2011.01571.x.
782. Courvoisier, D.S.; Eid, M.; Lischetzke, T.; Schreiber, W.H. Psychometric properties of a computerized mobile phone method for assessing mood in daily life. *Emotion (Washington, D.C.)* **2010**, *10*, 115–124. Place: United States Publisher: American Psychological Association, https://doi.org/10.1037/a0017813.
783. Meltzer, E.O.; Kelley, N.; Hovell, M.F. Randomized, cross-over evaluation of mobile phone vs paper diary in subjects with mild to moderate persistent asthma. *The open respiratory medicine journal* **2008**, *2*, 72–79. Place: United Arab Emirates Publisher: Bentham Open, https://doi.org/10.2174/1874306400802010072.
784. Pärkkä, J.; Merilahti, J.; Mattila, E.M.; Malm, E.; Antila, K.; Tuomisto, M.T.; Saarinen, A.V.; van Gils, M.; Korhonen, I. Relationship of psychological and physiological variables in long-term self-monitored data during work ability rehabilitation program. *IEEE transactions on information technology in biomedicine : a publication of the IEEE Engineering in Medicine and Biology Society* **2009**, *13*, 141–151. Place: United States Publisher: Institute of Electrical and Electronics Engineers, https://doi.org/10.1109/TITB.2008.2007078.
785. Bromberg, M.H.; Connelly, M.; Anthony, K.K.; Gil, K.M.; Schanberg, L.E. Self-reported pain and disease symptoms persist in juvenile idiopathic arthritis despite treatment advances: an electronic diary study. *Arthritis & rheumatology (Hoboken, N.J.)* **2014**, *66*, 462–469. Place: United States Publisher: Wiley, https://doi.org/10.1002/art.38223.
786. Thrul, J.; Bühler, A.; Ferguson, S.G. Situational and mood factors associated with smoking in young adult light and heavy smokers. *Drug and alcohol review* **2014**, *33*, 420–427. Place: Australia Publisher: Wiley-Blackwell, https://doi.org/10.1111/dar.12164.
787. Hareva, D.H.; Okada, H.; Kitawaki, T.; Oka, H. Supportive intervention using a mobile phone in behavior modification. *Acta medica Okayama* **2009**, *63*, 113–120. Place: Japan Publisher: Okayama University Medical School, https://doi.org/10.18926/AMO/31830.
788. Garcia, C.; Hardeman, R.R.; Kwon, G.; Lando-King, E.; Zhang, L.; Genis, T.; Brady, S.S.; Kinder, E. Teenagers and texting: use of a youth ecological momentary assessment system in trajectory health research with latina adolescents. *JMIR mHealth and uHealth* **2014**, *2*, e3. Place: Canada Publisher: JMIR Publications Inc, https://doi.org/10.2196/mhealth.2576.
789. Kirk, G.D.; Linas, B.S.; Westergaard, R.P.; Piggott, D.; Bollinger, R.C.; Chang, L.W.; Genz, A. The exposure assessment in current time study: implementation, feasibility, and acceptability of real-time data collection in a community cohort of illicit drug users. *AIDS research and treatment* **2013**, *2013*, 594671. Place: United States Publisher: Hindawi Pub. Corp, https://doi.org/10.1155/2013/594671.
790. Palmier-Claus, J.E.; Ainsworth, J.; Machin, M.; Barrowclough, C.; Dunn, G.; Barkus, E.; Rogers, A.; Wykes, T.; Kapur, S.; Buchan, I.; et al. The feasibility and validity of ambulatory self-report of psychotic symptoms using a smartphone software application. *BMC psychiatry* **2012**, *12*, 172. Place: England Publisher: BioMed Central, https://doi.org/10.1186/1471-244X-12-172.
791. Pramana, G.; Parmanto, B.; Kendall, P.C.; Silk, J.S. The SmartCAT: an m-health platform for ecological momentary intervention in child anxiety treatment. *Telemedicine journal and e-health : the official journal of the American Telemedicine Association* **2014**, *20*, 419–427. Place: United States Publisher: Mary Ann Liebert, Inc, https://doi.org/10.1089/tmj.2013.0214.
792. Doherty, S.T.; Lemieux, C.J.; Canally, C. Tracking human activity and well-being in natural environments using wearable sensors and experience sampling. *Social science & medicine (1982)* **2014**, *106*, 83–92. Place: England Publisher: Pergamon, https://doi.org/10.1016/j.socscimed.2014.01.048.

793. Mulvaney, S.A.; Rothman, R.L.; Dietrich, M.S.; Wallston, K.A.; Grove, E.; Elasy, T.A.; Johnson, K.B. Using mobile phones to measure adolescent diabetes adherence. *Health psychology : official journal of the Division of Health Psychology, American Psychological Association* **2012**, *31*, 43–50. Place: United States Publisher: American Psychological Association, Division of Health Psychology, https://doi.org/10.1037/a0025543.
794. Schnall, R.; Okoniewski, A.; Tiase, V.; Low, A.; Rodriguez, M.; Kaplan, S. Using text messaging to assess adolescents' health information needs: an ecological momentary assessment. *Journal of medical Internet research* **2013**, *15*, e54. Place: Canada Publisher: JMIR Publications, https://doi.org/10.2196/jmir.2395.
795. Jean, F.A.M.; Sibon, I.; Husky, M.; Couffinhal, T.; Swendsen, J. Feasibility and validity of Ecological Momentary Assessment in patients with acute coronary syndrome. *BMC Cardiovascular Disorders* **2020**, *20*, 499. https://doi.org/10.1186/s12872-020-01774-w.
796. Burns, M.N.; Begale, M.; Duffecy, J.; Gergle, D.; Karr, C.J.; Giangrande, E.; Mohr, D.C. Harnessing Context Sensing to Develop a Mobile Intervention for Depression. *Journal of Medical Internet Research* **2011**, *13*, e55. https://doi.org/10.2196/jmir.1838.

Disclaimer/Publisher's Note: The statements, opinions and data contained in all publications are solely those of the individual author(s) and contributor(s) and not of MDPI and/or the editor(s). MDPI and/or the editor(s) disclaim responsibility for any injury to people or property resulting from any ideas, methods, instructions or products referred to in the content.
